# Locating Undocumented Wells Using Historical Oil and Gas Exploration Maps: A Case Study in Osage County, Oklahoma

**DOI:** 10.1021/acs.est.6c03981

**Published:** 2026-07-16

**Authors:** Jaisree Iyer, Janice Yang, Whitney Kirkendall, Samuel O’Connor, Benjamin Le, Hewei Tang, Andre Santos, Sébastien C. Biraud, Fabio Ciulla, Charuleka Varadharajan

**Affiliations:** 1 4578 Lawrence Livermore National Laboratory, 7000 East Ave., Livermore, California 94550, United States; 2 1666 Lawrence Berkeley National Laboratory, 1 Cyclotron Road, Berkeley, California 94720, United States

**Keywords:** forgotten, lost, unknown or undocumented wells, automated symbol detection, YOLO, georeferencing, dry holes, orphaned wells, oil and gas maps

## Abstract

Undocumented oil and gas wells lack reliable information about their locations and characteristics, making them difficult to identify. These wells can result in unanticipated delays and costs in the development of nearby surface and subsurface resources, and, if improperly plugged, can cause contamination. This study leverages historical petroleum exploration maps to locate such wells, focusing on Osage County, Oklahoma. Two sets of early 20th century oil and gas exploration maps by the United States Geological Survey were georeferenced and analyzed using a computer vision model to detect well symbols. The locations of detected wells were compared to the location of known wells in the database from the Bureau of Indian Affairs Osage Agency to identify potential undocumented wells. The analysis yielded over 500 potential undocumented wells, with dry holes constituting the largest fraction. Field verification confirmed the presence of some undocumented wells. Comparison with prior work revealed limited overlap, underscoring the complementary value of historical oil and gas maps for locating undocumented wells. This approach demonstrates the utility of integrating historical cartographic resources with modern geospatial and machine learning techniques to improve the identification and management of undocumented wells.

## Graphical abstract

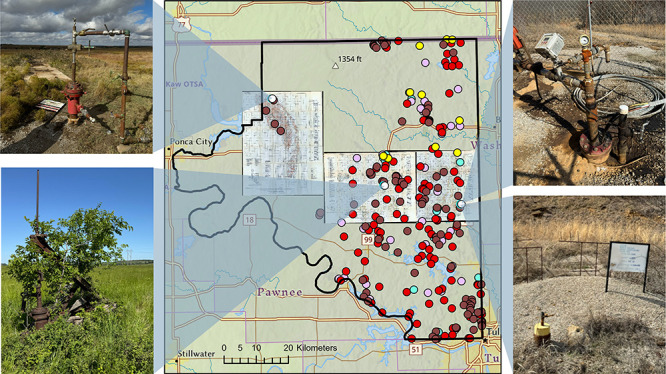

## 1 Introduction

The use of wells to produce oil and gas in the United States (US) dates back to the mid-1800s.^1^ Since then, we know of more than four million wells that have been drilled as part of oil and gas production activities in the US.^2,3^ Records for these wells are typically maintained by various regulatory agencies^4,5^ and commercial well databases.^6,7^ For example, a database of wells for Osage county, Oklahoma provided by the Bureau of Indian Affairs (BIA) Osage Agency is available through Osage Minerals Council^4^ and that for California is available through California Geologic Energy Management Division.^5^ Some examples of commercial databases include those offered by WellDatabase^7^ and S&P Global.^6^

Over the long history of oil and gas exploration in the US, a large number of wells are believed to be undocumented. An undocumented well refers to an oil or gas well that is either entirely unknown to the regulatory agency or requires verification to confirm its existence.^8^ Once discovered, these wells will likely become orphaned wells – wells without a responsible, solvent owner. Estimates for the number of undocumented wells vary widely. In 2020, the Interstate Oil & Gas Compact Commission (IOGCC) estimated the number of undocumented wells in the US to be between 310,000 and 800,000.^8^ In 2023, this estimate was revised to between 250,000 and 740,000.^9^ The IOGCC report notes that these estimates are imprecise as some states report a wide range while others are unable to provide reliable figures. State regulators that do provide an estimate typically rely on historical records, extrapolation based on discovered wells, and studies of orphaned wells.^8^ This uncertainty is further illustrated by a recent study estimating the number of undocumented wells based on oil production in Pennsylvania and Oklahoma at about 340,000 and 300,000, respectively.^10^ While the estimate for Pennsylvania falls within IOGCC’s range of 100,000 to 560,000, the estimate for Oklahoma is about five times higher than the most recent IOGCC number of 61,049.^9^

The presence of undocumented wells can result in unanticipated delays and costs, hindering the use of nearby surface and subsurface resources. For example, an examination of the well records with unknown lease name in the oil and gas wells database^5^ maintained by the California Geologic Energy Management Division revealed that the existence of several wells, previously unknown to the agency, was established during construction of buildings, offices, public transportation and utilities (Section S1 in the Supporting Information (SI) provides additional information on these wells). Additional work was then required to properly plug and abandon these wells before planned construction could proceed. If not plugged to modern regulatory standards, these wells can also emit pollutants (Section S2 in the SI provides examples of two such wells).^11,12^ To reduce the uncertainty surrounding undocumented wells, recent efforts have used various methods to locate them. Methods tested so far include analysis of historical photographs^13^ and topographical maps,^14^ as well as aeromagnetic^15–17^ and lidar^13^ surveys. These examples illustrate the range of approaches that have been explored for locating undocumented wells.

Of these methods, aerial magnetometer surveys have been used to locate wells for several decades.^18^ They are very effective in locating cased wells and can survey areas ranging from a few to tens of square kilometers. Their effectiveness for finding undocumented wells, which may be scattered across large areas, can be improved significantly by first identifying smaller target areas using historical records such as maps, as demonstrated by Ciulla et al.^14^ Ciulla et al. identified the locations of 1301 potential undocumented wells across over 40,000 km^2^ by applying a U-Net deep learning model to quadrangles in the United States Geological Survey (USGS) Historical Topographical Map Collection (HTMC). These results were subsequently used by ground-based teams that confirmed the location of some undocumented wells.^14^

Here we extend the method developed by Ciulla et al.^14^ to a different set of historical maps developed specifically by governmental agencies and industry operators for oil and gas exploration. The primary goal of this study is to determine if this different source of data can provide additional useful information about undocumented wells that is not available through the USGS HTMC quadrangles. There are several differences between the USGS HTMC quadrangles and historical oil and gas exploration maps. The USGS HTMC quadrangles are a well-curated collection of georeferenced maps available for most of the US and were developed to provide a snapshot of the nation’s physical and cultural features.^19^ These maps document the locations of wells along with other natural and engineered structures such as vegetation, streams, roads, buildings, and mines. Furthermore, Ciulla et al.^14^ focused on maps from 1947 to 1992 due to consistency in colors and symbols. In contrast, historical oil and gas maps are not available as a well-maintained data set from a single agency, are typically not georeferenced, and lack consistency, as they were drawn by cartographers from different organizations including private companies, local regulators and state or federal geological surveys. However, they are a promising resource for locating undocumented wells because they were developed specifically for oil and gas exploration and only document relevant geological features and well information such as location, status and depth. As a result, they may include information about wells that have been permanently taken out of service (abandoned wells) or exploratory boreholes that were not economically viable (dry holes). Such wells may lack surface features and therefore, may not be documented in the USGS HTMC. In addition, historical oil and gas exploration maps date back as far as the early 1900s and thus can potentially cover a time period missing from the analysis by Ciulla et al.^14^

To place our study in the context of other research on locating undocumented wells, we focused this proof of concept work on Osage county, Oklahoma, which has been a common area for the studies by Jahan et al.^10^ and Ciulla et al.^14^ Additionally, our team had access to conduct field work in this county to verify the results of this study. Osage County in Oklahoma was purchased by the United States Government from the Cherokee Nation in 1872 for use as a reservation for the Osage Tribe.^20^ The drilling of the first well in Osage County began in 1896, with oil production peaking in the 1920s.^20^ Several reports, primarily by the USGS^20–25^ and some by the Oklahoma Geological Survey^26–28^ were published to provide information on subsurface geology and to support oil and gas production. Some of these reports included detailed maps documenting well locations and local geology. In particular, the USGS Bulletins 686 ^24^ and 900 ^20^ from 1922 and 1942, respectively, contained detailed maps covering large portions of Osage county. Bulletin 686 contained 60 plates (full page illustrations that are separate from the main body of the text) and Bulletin 900 contained 20 plates. The maps in these two sets of plates form the basis of this study. They contain information about well location, well type, lease ownership, subsurface lithology, structure contours and location of faults (see example maps in Figure 1 and Section S3 in the SI). In addition to their extensive coverage of Osage county, these maps predate the USGS HTMC quadrangles used by Ciulla et al.^14^ Comparing maps from three different time points allows us to assess whether these data sets provide complementary information for finding undocumented wells.

**Figure 1:**
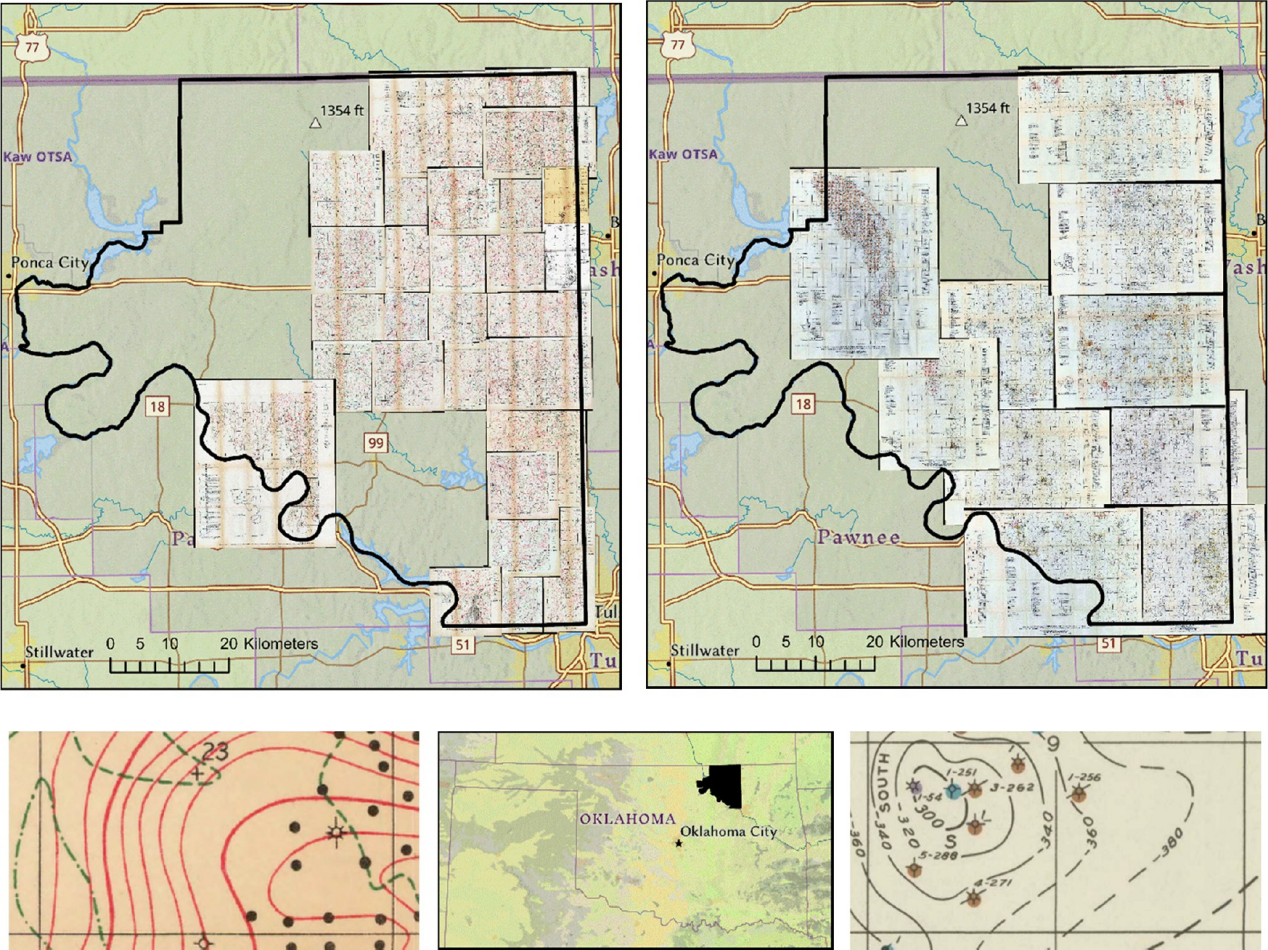
Location of georeferenced maps from Bulletins 686 (top left) and 900 (top right). The boundary of Osage county is outlined in black. Neither set of maps covers parts of western and northern Osage county. For context, the bottom panel has small portions of one of the Bulletin 686 (bottom left) and 900 (bottom right) maps showing the geological contour lines and the different well symbols. In addition, the location of Osage county within Oklahoma is shown in black in the bottom center panel.

## 2 Methods

### 2.1 Georeferencing Procedure

The historical maps were georeferenced using a semiautomated workflow that leveraged the georeferencing tool in ArcGIS Pro.^29^ Each historical map used in this study had an underlying Public Land Survey System (PLSS) grid drawn on the map (Figure S1 in the SI). The georeferencing method involved identifying four control points on the historical map and aligning them with their corresponding real-world coordinates derived from the standardized Bureau of Land Management (BLM) PLSS data layer.^30^ The four control points were selected as the corners of four distinct PLSS sections distributed across the historical map to ensure spatial coverage.

Once the control points were established, ArcGIS Pro’s georeferencing tool was used to align the historical map so that it accurately overlaid the BLM PLSS grid. Since the historical maps were scanned images, the projective transformation was used to align the maps, and the cubic resampling method was used to interpolate pixel values. Projective transformation with four control points forms an exactly determined system. Hence, the root-mean-square error associated with the georeferencing process is zero. The resulting georeferenced maps were reviewed for alignment accuracy by comparing the map PLSS grid lines with those in the BLM PLSS data layer. Some discrepancies are expected due to refinements in the modern PLSS grid not reflected in historical maps. To estimate the error we can anticipate due to this discrepancy, for each georeferenced map, the maximum georeferencing error was estimated by visually comparing the PLSS township-range grid on the georeferenced map to the BLM PLSS grid (Section S4 in the SI). The measure tool in ArcGIS Pro was used to estimate the physical distance between the two grids at the point of largest discrepancy. The same tool was used to also estimate the size of the symbols in the maps.

### 2.2 Automated Well Symbol Detection

Each of the historical oil and gas maps had between tens and thousands of wells located on them. To facilitate their identification, we developed a computer vision (CV) model. CV methods are a class of machine learning methods that enable automated extraction of information from images and were used here to identify well symbols in historical maps. Typical CV approaches include semantic segmentation,^31^ wherein individual pixels within an image are assigned classes and then aggregated to identify objects, and object detection,^32^ wherein entire objects are classified using bounding boxes. The maps in this study substantially differ from the HTMC in background, scale, and symbol representation. Hence, we developed a different model tailored to the maps for this study. Specifically, a YOLO (You Only Look Once)-based object detection model was developed to identify well symbols, as the object detection framework was well suited for identifying multiple discrete well symbol types embedded within the Bulletin maps.

Several maps exhibiting diverse characteristics such as map color and well symbol appearance were chosen and manually labeled. Well symbols corresponding to all types of drilled wells were included (active and abandoned wells; dry holes, oil and gas wells). In total, four coauthors annotated 7113 wells from five Bulletin 900 maps, and 630 wells from 12 Bulletin 686 maps. A significantly larger number of Bulletin 900 wells were annotated because this work originally started with the Bulletin 900 maps and was later extended to Bulletin 686 maps. Additionally, a typical Bulletin 900 map has significantly more wells than a Bulletin 686 map.

The YOLOv5x model,^33^ commonly used for its fast single-shot object detection capabilities, was used to localize the well symbols. For training the CV model, all georeferenced maps were preprocessed by creating smaller patches to provide better resolution for the CV model. Larger maps were split into a 30 × 30 grid to create 900 smaller patches while smaller maps were split to maintain a minimal patch size of 1000 × 1000 pixels. 20% of labeled patches were used for validation, and the rest were used for training. The model was fine-tuned on the labeled map patches for 100 epochs with the low-augmentation default hyperparameters and achieved a mean average precision for an intersection over union threshold of 0.5, mAP@0.5, of 0.9325 on the validation set. This indicates that the model correctly identified and localized features in the validation images with an average accuracy of 93.25%, where a detection was considered correct only if it spatially overlapped with the true feature boundary by at least 50%. The model was then used to provide well detections for all unlabeled maps using a confidence threshold of 0.3 to ensure as many potential well detections were kept as possible. Even so, almost all false positives are contained in the legends of maps and are easily manually removed. Detections in the legends of maps are considered false positives because they are markers for the maps and not actual location of wells. They were also excluded during manual annotation for creating a training data set.

### 2.3 Identification Criteria for Potential Undocumented Wells

The location of each well present on a historical map was compared to the locations of all known wells in Osage county. For the purpose of this comparison, the well locations provided by the Bureau of Indian Affairs (BIA) Osage Agency, available through Osage Minerals Council^4^ was used as the reference data set. This database contains the locations of 43,822 wells within Osage county. None of these wells are present in the well database maintained by the Oklahoma State regulator, Oklahoma Corporation Commission.^34^ A well from a historical map was classified as a potential undocumented well if its distance to the nearest well in the BIA Osage Agency database exceeded a threshold. This threshold distance was separately determined for each set by quantifying the size of the well symbol on the map, the georeferencing errors, and the typical nearest-neighbor distance among known wells in the BIA Osage Agency database.

### 2.4 Field Verification

To verify the presence of potential undocumented wells, field verification was conducted using the same tiered visual and magnetic method described by Ciulla et al.^14^ Oil and gas wells typically have a steel pipe, known as casing, to provide structural integrity and to prevent surface and subsurface contamination. The presence of casing can be detected using a magnetometer. For wells lacking surface expression, we used a Geometrics G-864 total-field cesium vapor magnetometer^35^ paired with GPS (Global Positioning System) to collect total magnetic field measurements while walking a gridded pattern. This instrument enables the detection of distinct, localized magnetic anomalies associated with buried ferromagnetic well casings. Such anomalies exhibit strong dipolar magnetic signatures generated by vertical steel casings. While often approximated as monopolar in detection-focused workflows, they are treated here as dipolar sources, consistent with the physics of finite-length, vertically magnetized casing. This representation was also used in related characterization studies conducted by our team. These signatures are readily distinguishable from the background magnetic field obtained from the NOAA (National Oceanic and Atmospheric Administration) World Magnetic Field Model.

## 3 Results and Discussion

### 3.1 Georeferenced Map Coverage and Positional Uncertainty

Of the 60 plates in USGS Bulletin 686, 28 were georeferenced using the method described in Section 2.2. The remaining plates were excluded as they were not maps, did not contain wells, or were duplicates. Similarly, of the 20 plates in USGS Bulletin 900, 10 maps were georeferenced. These georeferenced maps are available for download (Section S3 in the SI), and their spatial extent is shown in Figure 1. All map-based figures were drawn using basemaps provided by ArcGIS Pro.^36^

For each of the 38 georeferenced maps, the maximum georeferencing error was estimated (Section S4 in the SI). The average of the maximum georeferencing error across all maps was 65 m. This is significantly larger than the average ground sampling distance (GSD) for the maps, which is 0.8 m for the Bulletin 686 maps and 2 m for the Bulletin 900 maps (Section S5 in the SI). The GSD reflects both the original map scale and the resolution used when converting the PDF maps to raster format. An additional source of error in the well location arises from the size of the symbols used to denote wells. Bulletin 686 maps use circular symbols with a diameter of 60 m while Bulletin 900 maps use circular symbols with a diameter of 30 m. Therefore, the expected error due to symbol size is approximately 30 m for Bulletin 686 maps and 15 m for Bulletin 900 maps.

### 3.2 Well Detection

The CV model described in Section 2.3 was applied to all 38 georeferenced maps, shown in Figure 1, to detect symbols used to locate wells on the maps. For the 28 maps in Bulletin 686, the model identified 5,210 well-symbols. Of these, 153 detections corresponded to well symbols in the map legends and were excluded from the analysis as they do not represent the actual physical location of wells and serve as example markers for the rest of the map. An additional 222 detections were located in overlapping map areas or in the map margins and were also excluded. The remaining 4,988 wells are shown in Figure 2.

**Figure 2:**
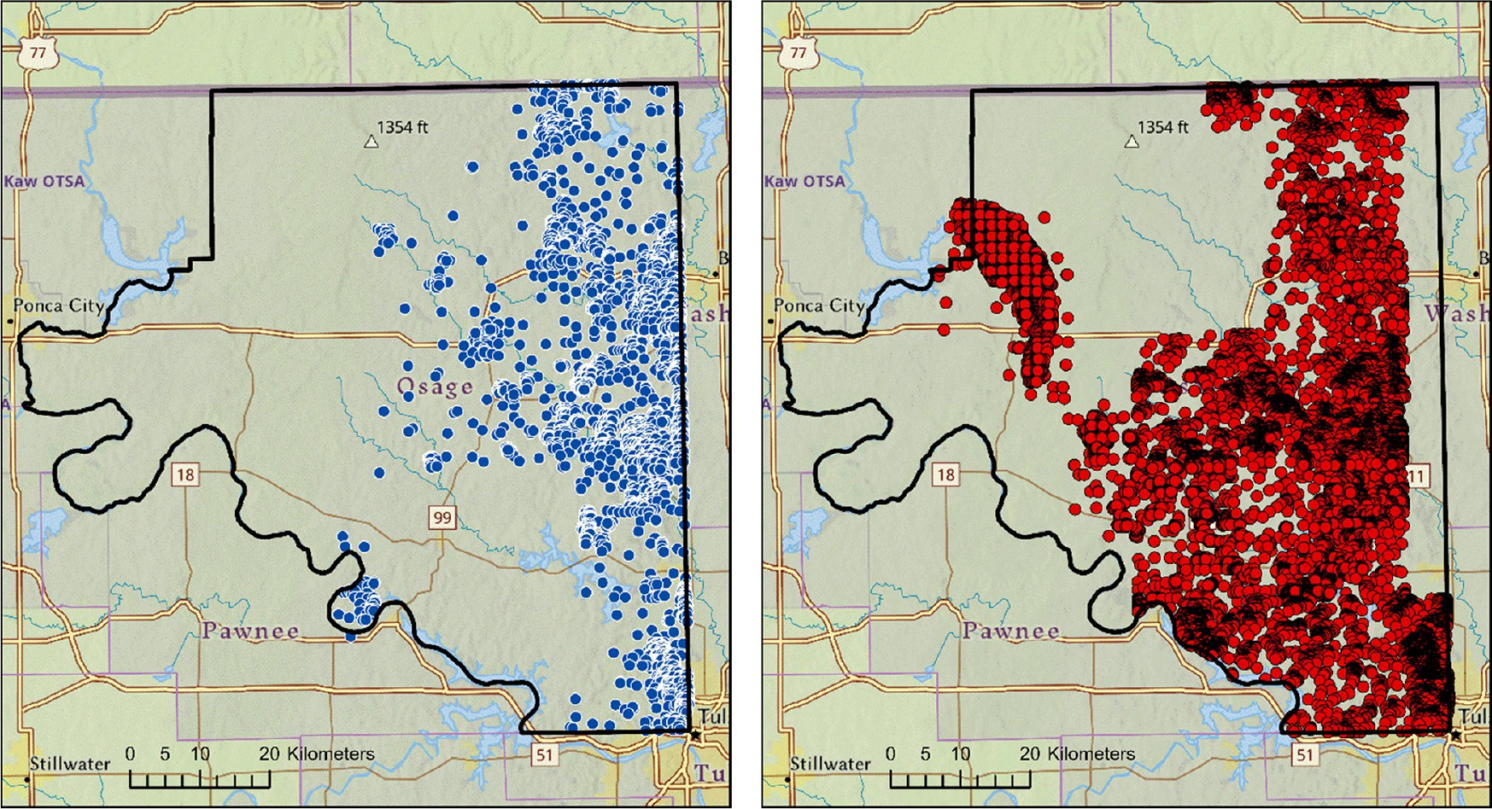
Location of wells detected using the CV model on the maps in Bulletins 686 (left) and 900 (right).

Not all detections were manually verified. An analysis of the distance to the nearest neighbor revealed that 364 detected wells (7%) had a neighboring well within 35 m. A spot check of 10 such wells confirmed that all were duplicates, i.e., a second detection for an already detected well, indicating that some fraction of the 364 detections are duplicates (false positives). Without comprehensive manual verification, the exact number of false positives remains uncertain as some wells on the maps may have more than two detections. A visual inspection of the maps and the CV model detections identified 3 additional wells that were incorrectly detected (false positives) and 75 wells (1.5%) that were missed by the CV model (false negatives). There may be more, as this inspection was not thorough and manually verifying 4988 wells is extremely tedious. Instead, comprehensive manual verification was done for all detected wells that were identified as undocumented (Section 3.3) as that is the primary result of this work.

For the 10 maps in Bulletin 900, the CV model detected 15,214 wells shown in Figure 2. As with Bulletin 686 maps, not all detections were manually verified. Similar to Bulletin 686 maps, a distance analysis showed that 262 detected wells (2%) had a neighbor within 35 m. Visual inspection identified at least 47 wells (<1%) on the map that were not detected by the CV model. Most of these missing detections were present in dense clusters of wells. Overall, the CV model performed better on the 10 Bulletin 900 maps than on the 28 Bulletin 686 maps. This could be because the CV model was trained using more labeled wells from the Bulletin 900 maps. Moreover Bulletin 900 has more consistency in maps and well symbol appearance compared to Bulletin 686 maps.

Well counts from each of our maps showed good agreement with the well counts listed in the associated volume in Bulletin 900 (Section S5 in the SI). According to Bulletin 900, Osage Agency records showed 17,604 wells in Osage county on December 1, 1939.^20^ This is 2390 more than our detected wells from the Bulletin 900 maps. This discrepancy is likely because the maps do not cover all of Osage county and wells in the areas not covered by the maps are not accounted for in our well count of 15,214.

### 3.3 Identification of Potential Undocumented Wells

We identified potential undocumented wells from the well symbols recorded in Figure 2 by selecting those from the Bulletin maps with no corresponding BIA Osage Agency database well within a specified threshold distance. This threshold distance should be larger than the errors associated with georeferencing and symbol size because these determine the accuracy with which the method described in Section 2 can locate wells. This corresponds to 95 and 80 m for maps in Bulletins 686 and 900, respectively. These distances are similar in magnitude to the 100 m threshold distance used by Ciulla et al.^14^

The threshold distance should also be small enough to not conflate two distinct wells. To determine an upper limit on the threshold distance, the nearest neighbor distance in the BIA Osage Agency database is analyzed in Figure 3. It shows that almost 80% of the wells have another well within 200 m. Choosing a cutoff distance of >200 m increases the risk of associating a well identified from the Bulletin maps to an incorrect well in the BIA Osage Agency database.

**Figure 3:**
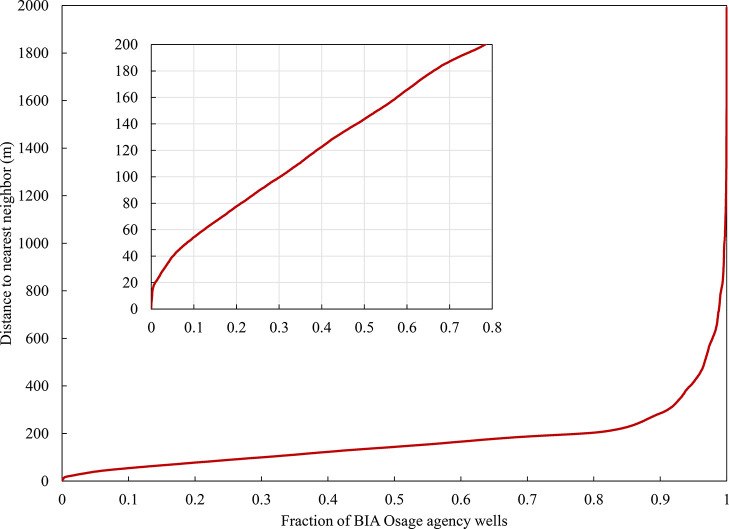
Nearest neighbor distance for wells in the BIA Osage Agency database. The inset zooms into the portion of the curve that has a nearest neighbor distance less than 200 m.

Given the uncertainty associated with the choice of the threshold distance, Figure 4 plots the fraction of wells found in the Bulletin maps that would be identified as potential undocumented wells as a function of the threshold distance. This fraction varied between 3–23% for Bulletin 686 wells and 1–6% for Bulletin 900 wells when the threshold distance is decreased from 200 to 80 m. Across all threshold distances, a larger fraction of the Bulletin 686 wells is classified as potential undocumented wells compared to Bulletin 900 wells. Part of this systematic difference could be due to 11% of the Bulletin 686 wells being within 100 m of the county boundary compared to only 5% for the Bulletin 900 wells. Wells closer to the county boundary could be misidentified as undocumented because a portion of the surrounding area within the threshold distance falls outside the county boundary and this external area is not covered by the BIA Osage Agency database.

**Figure 4:**
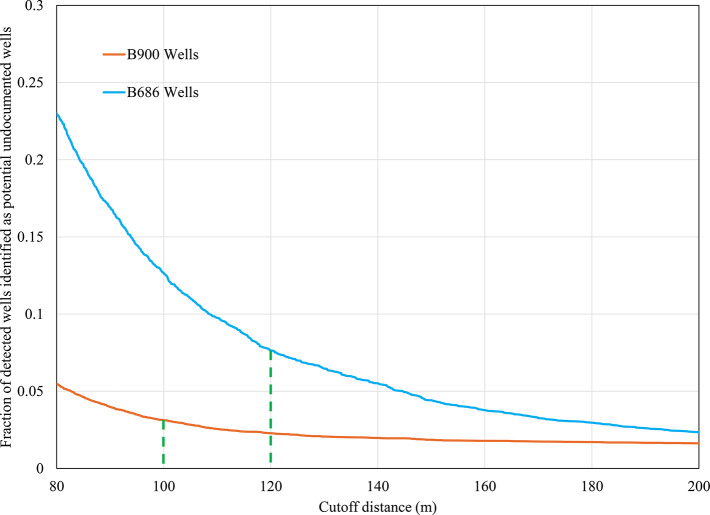
Fraction of wells detected from Bulletins 686 (blue) and 900 (orange) that are identified as potential undocumented wells based on the choice of threshold distance. The total well counts for Bulletins 686 and 900 are 4988 and 15,214, respectively. The two vertical green dashed lines at 100 and 120 m indicate the threshold distance used in this study for maps in Bulletins 900 and 686, respectively.

To further analyze the location and characteristics of the potential undocumented wells identified from these two bulletins, we chose a threshold distance of 120 and 100 m for Bulletin 686 and 900 maps, respectively. These threshold distances are about 25% more than the minimum threshold distance determined using the error estimates described before. These threshold distances assume that the locations of all wells in the BIA Osage Agency database are accurate. A 120 m threshold distance identified 382 potential undocumented wells in the Bulletin 686 maps. Manual verification of all 382 wells led to the removal of 8 false positives (2%) and 9 duplicates (2%). This reduced the count for the potential undocumented wells to 365 wells shown in Figure 5 and Section S6 in the SI. A 100 m cutoff distance yielded 475 potential undocumented wells in Bulletin 900 maps. This included 211 in the Burbank oil field near Kaw Lake outside Osage county. This cluster is on the top left corner in Figure 2. These wells were excluded because the BIA Osage Agency database only covers Osage county. Figure 5 shows the remaining 264 wells, all of which were manually confirmed as unique true positives.

**Figure 5:**
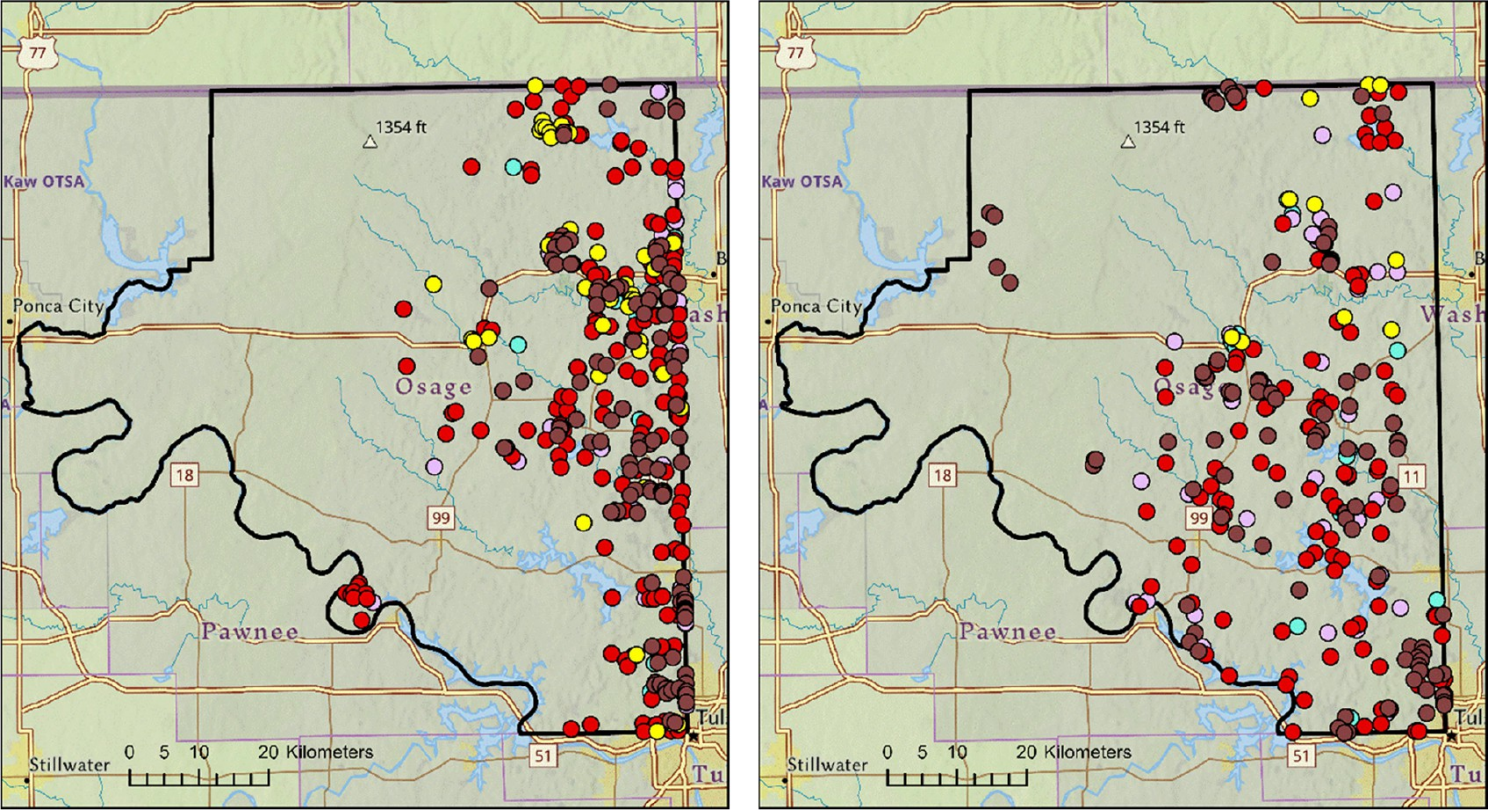
Location of 365 and 264 potential undocumented wells identified from maps in Bulletins 686 (left) and 900 (right), respectively. The symbols are colored based on the well type described in Section 3.7: blue - abandoned gas; purple - abandoned oil; red - dry holes, yellow – gas; and brown - oil.

### 3.4 Field Verification

Field verification was conducted using the method described in Section 2.4 to verify the presence of eight potential undocumented wells identified in Section 3.3. Visual inspection confirmed the presence of wellheads at four potential undocumented well locations within the Osage county: well 86 in Section 33 of T25N-R9E (distance from CV model detection, D = 93.4 m), wells 121 and 123 in Section 24 of T24N-R11E (D = 18.7 and 19.43 m, respectively), and well 323 in Section 1 of T27N-R5E (D = 21.2 m). The well numbers correspond to the IDs described in Section S6 in the SI. None of these wells are listed in the BIA Osage agency database. However, these wells are present in the IHS (now S&P Global) database provided by Osage Mineral Council.^37^ Three of these four locations have signage plates on the ground. We could not use the IHS database to identify potential undocumented wells in this study as the authors of this paper do not have access to this commercial database.

Magnetometry also revealed a buried well (4.6 μ T enhanced anomaly) near the location of well 114 in Section 12 of T24N-R10E (D = 11.5 m), where neither the BIA nor IHS databases indicated the presence of a well. The anomaly reached 4.6 μ T (4,600 nT) above the survey background, defined here as the median of the total field measurements across the survey area. In the northern portion of Osage County, we investigated three potential undocumented well locations, well 170 in Section 17 and wells 173 and 174 in Section 20 of T29N-R9E, but no visual or magnetic evidence of well infrastructure was found. This absence may reflect historical well-abandonment practices. During the early to mid-20th century, it was common to extract casing from depleted wells^38^ for economic reasons, particularly during wartime. The Plugging Record form used by the Department of Interior in Osage county has a field to document the details of the casings that were pulled out suggesting this was common in Osage county (Section S7 in the SI). This practice left insufficient ferrous material to generate detectable magnetic signatures, which could potentially explain the lack of magnetic signatures for some potential undocumented wells.

### 3.5 Overlap across Different Data Sources

We currently have three lists of potential undocumented wells from three sets of maps for Osage county, namely Bulletin 686, Bulletin 900, and HTMC. Given these maps are from different points in time, evaluating the overlap among these data sets would help determine whether older maps provide unique information not captured by newer sources. Using their respective cutoff distances, the three maps sets in Bulletin 686, 900 and HTMC, yielded 365, 264, and 261 potential undocumented wells, respectively, in the 3216, 3475, and 5967 km^2^ area they cover within Osage county. This corresponds to densities ranging between 4 and 11 potential undocumented wells per 100 km^2^ with higher densities in the older maps (Bulletin 686). These results indicate that historical oil and gas maps can reveal information not present in more recent data sets.

In comparison, the number of potential undocumented wells identified here is 2 orders of magnitude smaller than the estimate of over 30,000 undocumented wells in Osage county reported by Jahan et al.^10^ Our results in Figure 5 represent a lower bound as Bulletins 686 and 900 only cover wells drilled up to 1922 and 1942, respectively. Wells drilled after 1942 that became undocumented in the last 80 years are not included in this study. Moreover, the absence of spud date (the date drilling begins) information for all wells in the BIA Osage Agency database further restricts the number of wells that are identified as potential undocumented wells. The method described in Section 2.4 identifies a well as potential undocumented well if the closest database well is beyond a threshold distance, regardless of the spud date. Ideally, the distance analysis to identify undocumented wells should be limited to database wells that were spudded on or before the historical map date. In the absence of spud dates, we assume that all database wells were spudded before the historical map date which underestimates the number of wells identified as undocumented. However, given the extremely large difference, it is unclear if correcting for this limitation would fully resolve the discrepancy with Jahan et al.’s^10^ estimate.

Finally, validating the presence of hundreds of wells scattered across thousands of square kilometers is a time-consuming and expensive exercise. High confidence targets for validation can be obtained by identifying wells that are present in more than one data source. By comparing the distance between potential undocumented wells from multiple maps we found that there are about 32–33 wells that are common between the Bulletin 686 and Bulletin 900 maps (one Bulletin 686 well was close to two Bulletin 900 wells, hence the discrepancy of 1), 10 common between the Bulletin 686 and HTMC maps and 14–16 common between Bulletin 900 and HTMC maps. These ∼50 wells could be considered as high confidence targets for ground campaigns to find undocumented wells. These overlaps represent less than 10% of the total wells identified as potential undocumented wells in each map series, further suggesting that different sources of data from different time points have the potential of providing complementary information about the location of potential undocumented wells.

### 3.6 Temporal Comparison of Map Sets

Using the method described in Section 2.4 we also compared the documented status of wells across map sets to assess change with time. Chronologically, the oldest set of maps are from Bulletin 686 (1922), followed by Bulletin 900 (1942), the HTMC (1956–1978) and the BIA Osage Agency database (current). Given the maps from Bulletins 686 and 900 do not cover all of Osage county, understanding what fraction of the wells in the maps in Bulletin 686 remain documented in Bulletin 900 was limited to the area of Osage county that is common to both map sets. In addition, the threshold distance for this comparison was increased to 175 m to account for cumulative errors from both map sets (80 and 95 m). Similar to Figure 4, the red curve in Figure 6 shows the fraction of Bulletin 686 wells that is within a certain distance from a Bulletin 900 well. More than 95% of the 2,927 Bulletin 686 wells in the Bulletin 900 map area are within 175 m of a Bulletin 900 well. Manual verification of the remaining 133 wells identified 5 false positives (4%) and 2 sets of duplicates (1.5%). This suggests that 126 Bulletin 686 wells from 1922 likely became undocumented by 1942 in the context of Bulletin 900.

**Figure 6:**
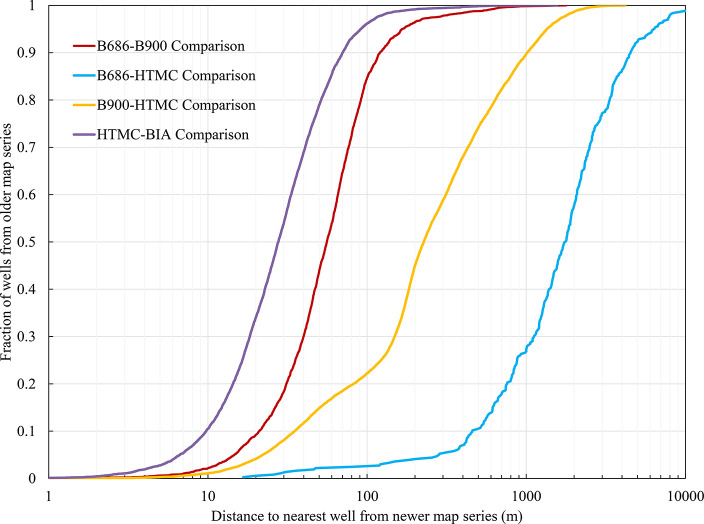
Fraction of wells detected from maps in older series (Bulletin 686, Bulletin 900 and HTMC) that are present within a threshold distance of a well from a newer maps series (Bulletin 900, HTMC and BIA Osage agency database).

A similar analysis on Bulletin 900 wells in the context of the HTMC shows that only 22% of the Bulletin 900 wells had an HTMC well within 100 m (assuming no error in HTMC well location). Manual verification of the 11,672 unmatched wells was not feasible. Similar comparisons between each pair of map sets are shown in Figure 7. Majority of the comparisons between the older and newer maps showed that 2–8% of the wells became undocumented in the context of the newer maps. The outliers include the two comparisons with the HTMC. The large number of wells that potentially became undocumented in the context of the HTMC as shown in Figures 6 and 7 cannot be only attributed to the accuracy of the two machine learning models that yielded these well counts. It is likely that these large discrepancies are due to difference in mapping methods. Wells in the HTMC were likely drawn based on their topographical features, and thus, may have omitted plugged and abandoned wells that lacked surface features. In fact, comparing total well counts also clearly highlights this gap. Bulletin 900 maps, covering 58% of Osage county, contain 15,002 wells while the HTMC, covering the entire county, has only 7,146. This suggests that the HTMC well counts may be at least 50% lower than the cumulative number of wells drilled by 1942. Note that many of the 13,755 HTMC wells in the work by Ciulla et al.^14^ are from more than one map for the same geographic location and therefore likely duplicates. While Ciulla et al.^14^ removed these redundancies within the subset of wells identified as potential undocumented wells, such redundancies were not removed from the full set of 13,755 HTMC wells, the majority of which are documented wells. In the analysis with the Bulletin 686 and 900 map wells presented here, for every quadrangle, we chose the well detections only from the latest HTMC map if multiple were available. This resulted in 7,146 wells, a 48% reduction from the 13,755 wells reported by Ciulla et al.^14^

**Figure 7:**
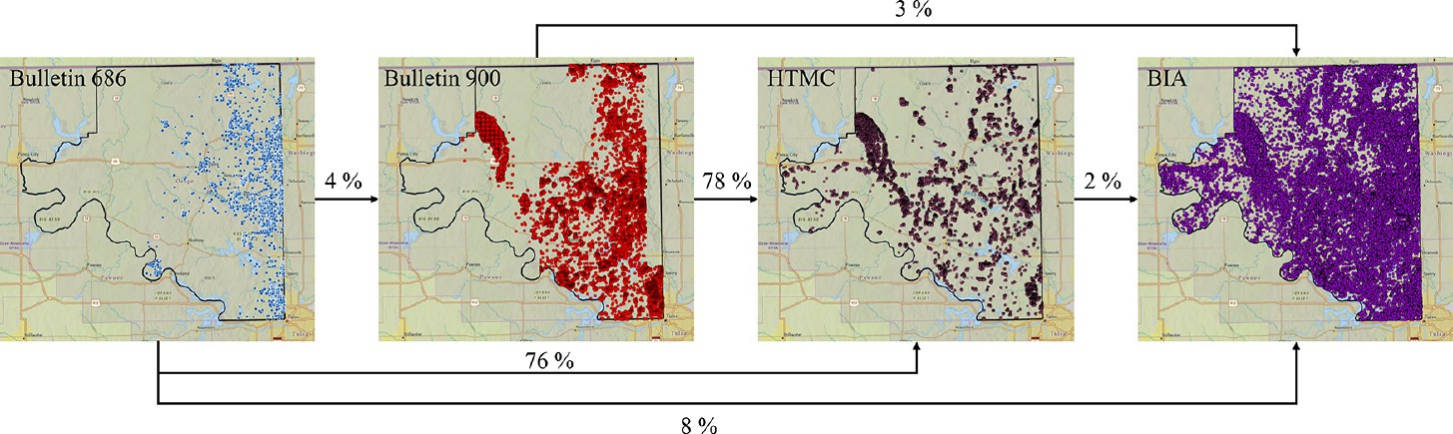
Fraction of wells that have gone missing between pairs of maps. The value for the HTMC-BIA comparison was taken from Ciulla et al.^14^

### 3.7 Well Types and Distribution

The maps associated with Bulletins 686 and 900 provide information about every well’s status in addition to its location. This information may shed light on which types of wells are more likely to become undocumented over time. Figure 8 summarizes the well types for the potential undocumented wells identified from these maps. Across both data sets, dry holes represented the largest fraction of potential undocumented wells, followed by active oil wells and then abandoned oil wells. A dry hole is an exploratory borehole that did not yield oil or gas in economically viable quantities to justify producing from it. The presence of a dry hole may also discourage others from undertaking further oil and gas exploration in nearby areas. This is indicated by the higher average distance to the nearest BIA Osage Agency well for a potential undocumented dry hole compared to that for a potential undocumented oil or gas well as shown in the labels in Figure 8. Dry holes accounted for more than half of the Bulletin 686 wells that were identified as potential undocumented wells in the context of Bulletin 900 in Figure 7. These results clearly indicate that dry holes form a large fraction of potential undocumented wells, and sources that have information about the location of exploratory boreholes may provide valuable information for finding potential undocumented wells.

**Figure 8:**
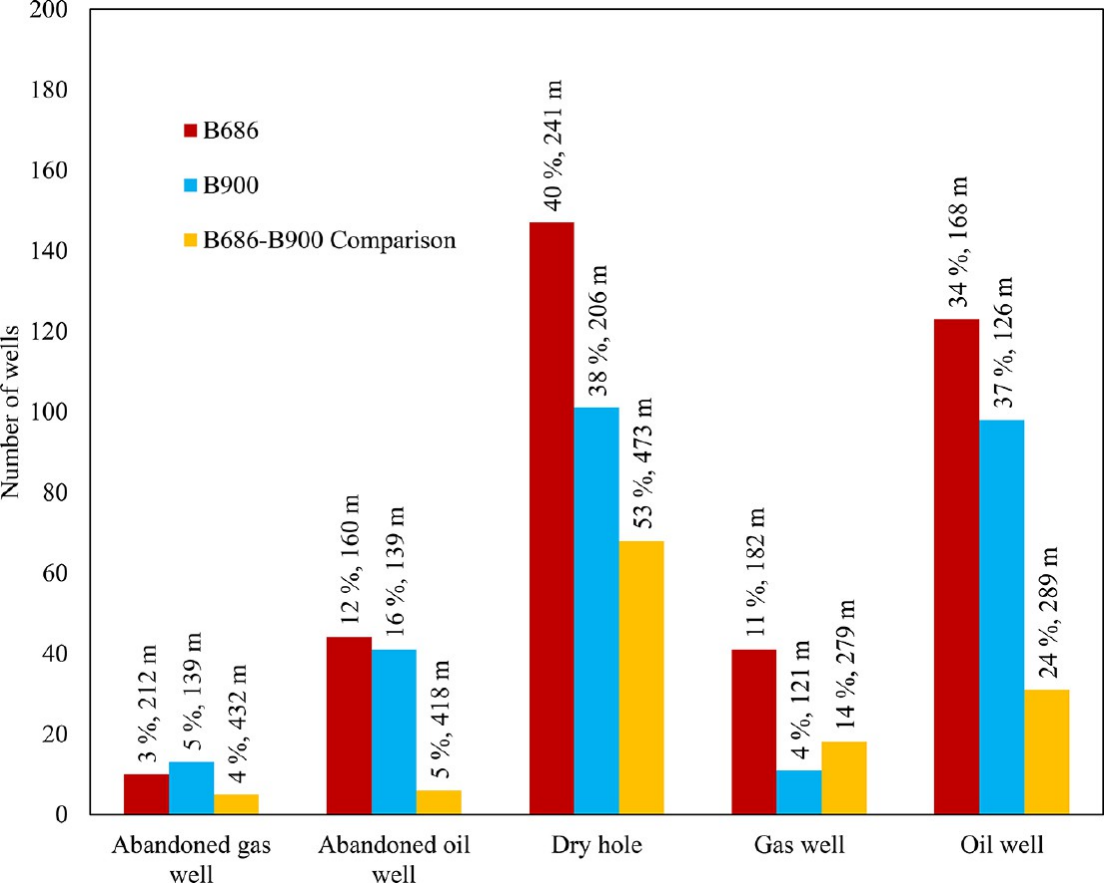
Distribution of potential undocumented wells from Bulletins 686 (red) and 900 (blue) by well type. The yellow bars show the distribution of potential undocumented wells from Bulletin 686 in the context of Bulletin 900. The fractional contribution of each well type and the average distance to the nearest BIA Osage Agency database well is indicated in the data label.

## 4 Implications and Recommendations

This study demonstrates that historical oil and gas exploration maps provide a valuable and complementary source of information for identifying potential undocumented wells, particularly when integrated with modern geospatial and machine learning approaches. This approach can be scaled up by improving accessibility of historical oil and gas records. While resources such as the USGS HTMC are well curated, they were not developed specifically for petroleum exploration and may omit wells otherwise documented in data sets for prospecting, as demonstrated in this study. For example, our results highlight that a large fraction of potential undocumented wells are dry holes and these wells may not be documented in the USGS HTMC. Additionally, they may not have a casing as they were likely not completed wells, and thus may not be detectable using magnetometry. Expanding historical data sets that include information on dry holes would be useful to find such undocumented wells. More importantly, establishing centralized, digitized, and searchable repositories of historical exploration maps and related records would significantly enhance the ability to apply automated detection techniques at scale and improve identification of undocumented wells.

Computer based identification of undocumented wells can also serve as high confidence targets for field verification. While there was limited overlap between wells identified from different map series in this study, combining historical maps from different periods can increase confidence in candidate locations and help prioritize targets for field verification. Such multisource integration, coupled with targeted validation using geophysical methods, offers a practical pathway for scaling efforts to identify undocumented wells over large geographic areas.

Current geospatial analyses used to determine whether a well detected from a historical data set is undocumented largely depends on information in regulatory well databases. Enhancing the completeness of regulatory well databases with spud-date information would substantially improve identification workflows. The current distance-based approach used to identify potential undocumented wells does not account for the timing of drilling activities due to lack of information. As a result, wells identified as “documented” may in fact postdate the historical maps, leading to systematic underestimation of undocumented wells. Incorporating accurate spud dates into regulatory data sets would enable spatiotemporal filtering and provide a more robust framework for identifying undocumented wells.

Additionally, methodological refinements are needed to address challenges associated with positional uncertainty when using map-based resources. Errors arising from georeferencing and symbol size introduce uncertainty in well locations, which becomes particularly problematic in areas with closely spaced wells. In such settings, purely distance-based criteria may be insufficient to distinguish between documented and undocumented wells. Future approaches should incorporate additional spatial features, such as local well density, clustering behavior, distribution patterns, and uncertainties in documented well locations, to improve classification accuracy in regions with high well density.

## Supplementary Material















## Notes

Supporting details about the methods and results (PDF)
Locations of potential undocumented wells and additional supporting data (XLSX)
The georeferenced maps are hosted on the Energy Data Exchange (EDX), the U.S. Department of Energy’s Hydrocarbons and Geothermal Energy Office’s virtual library and data laboratory. It is accessible from https://edx.netl.doe.gov/dataset/georeferenced-oil-and-gas-maps-of-osage-county-oklahoma-based-on-usgs-bulletins-686-and-900 or https://doi.org/10.18141/3376407. U.S. Geological Survey Bulletin 686 and 900 plates were used in compiling this information.
Well record for a well with API number 0401320381 (PDF)
Well record for well with API number 0403723863 (PDF)
First example of a plugging record (PDF)
Second example of a plugging record (PDF)
Third example of a plugging record (PDF)

## †

Present address: Hildebrand Department of Petroleum and Geosystems Engineering, The University of Texas at Austin, Austin, Texas 78712, USA
