# Supplementary material for: Locating Undocumented Wells Using Historical Oil and Gas Exploration Maps: A Case Study in Osage County, Oklahoma
Source: Environ Sci Technol. 2026 Jul 16;60(30):20913–24. doi: 10.1021/acs.est.6c03981 (PMC13449896; doi:10.1021/acs.est.6c03981)
Supplement: Supplementary file 1 [file es6c03981_si_001.pdf]

Supplementary Information for "*Locating Undocumented Wells Using Historical Oil and Gas Exploration Maps: A Case Study in Osage County, Oklahoma*"

Jaisree Iyer[1*], Janice Yang[1], Whitney Kirkendall[1], Samuel O'Connor[1], Benjamin Le[1], Hewei Tang[1], Andre Santos[2], Sebastien C. Biraud[2], Fabio Ciulla[2], and Charuleka Varadharajan[2]

[1]Lawrence Livermore National Laboratory, 7000 East Ave., Livermore, CA 94550, USA

[2]Lawrence Berkeley National Laboratory, 1 Cyclotron Road Berkeley, CA 94720, USA

*Corresponding author: Jaisree Iyer (jaisree@llnl.gov)

†Currently at Hildebrand Department of Petroleum and Geosystems Engineering, The University of Texas at Austin, Austin, TX 78712, USA.

Summary: 6 pages, 1 figure

S1. UNKNOWN WELLS IN CALIFORNIA

To find examples of undocumented wells that were discovered by regulators long after they were drilled, we evaluated all wells with "Unknown" in the "Lease Name" in the "All Wells" database[1] maintained by the California Geologic Energy Management (CalGEM) Division. A summary of these wells is provided in the spreadsheet "UnknownLeaseName" in the workbook es6c03981_si_002.xlsx. The columns specify the following information:

A. Serial No.: Serial number assigned by the authors
B. API: Unique ID for each oil and gas well. API stands for American Petroleum Institute.
C. LeaseName: Name of the Lease in the "All Wells" database.
D. Undocumented: The authors' evaluation whether the well was undocumented at some point in time. This field takes values: Yes, Maybe yes, No idea, Maybe no, and No indicating the authors' certainty in making this determination.
E. Notes: Short notes summarizing the authors' findings from the well records.
F. Identification method: The authors' determination of how the well was discovered.

While the primary subject of this paper is Osage county, Oklahoma, this type of analysis could not be performed with Bureau of Indian Affairs (BIA) Osage Agency database[2] due to unavailability of information

S2. EXAMPLES OF POLLUTING WELLS

Well records for two wells present in the spreadsheet described above, with API numbers 401320381 and 403723863, have been included here to show examples of undocumented wells that were releasing fluids into their surrounding at the time of discovery. The well records can be found in the files es6c03981_si_003.pdf and es6c03981_si_004.pdf which correspond to the files 01320381_DATA_11-19-2013.pdf and 03723863_1988-10-13_DATA.pdf obtained from CalGEM's online service.[3]

S3. GEOREFERENCED MAPS

B686.zip and B900.zip contain the 28 and 10 maps from United States Geological Survey (USGS) Bulletins 686[4] and 900[5], respectively, that were georeferenced as part of this study. These maps are hosted on the Energy Data Exchange (EDX), the US Department of Energy's,

Hydrocarbons and Geothermal Energy Office's virtual library and data laboratory. It is accessible from https://edx.netl.doe.gov/dataset/georeferenced-oil-and-gas-maps-of-osage-county-oklahoma-based-on-usgs-bulletins-686-and-900 or https://doi.org/10.18141/3376407. U.S. Geological Survey Bulletins 686 and 900 plates were used in compiling this information. The 38 maps were georeferenced based on the method described in Section 2.2. Each map has its own folder which include the georeferenced map image in tif or jpg format, a world file with spatial referencing information, and other files with metadata and auxiliary information for rendering the georeferenced maps in geographic information system software like ArcGIS Pro.[6]

S4. GEOREFERENCING ERROR

Figure S1 shows the typical georeferencing error as seen by the slight misalignment of the BLM (Bureau of Land Management) PLSS (Public Land Survey System) township-range grid lines[7] in red and the historical map PLSS grid lines in black. The maximum georeferencing error for each map evaluated based on visual inspection is documented in Column C of the spreadsheet "MapSummary" in es6c03981_si_002.xlsx.

S5. SUMMARY OF WELL DETECTIONS

Additional information about the wells detected from the 38 georeferenced maps is summarized in the spreadsheet "MapSummary" in the workbook es6c03981_si_002.xlsx. The columns in this spreadsheet specify the following information:

A. Report name: Name of the report from which the map originated. The field takes two values, B686 and B900, for Bulletins 686 and 900, respectively.
B. Plate No.: Plate ID identifying the map in a specific Bulletin.

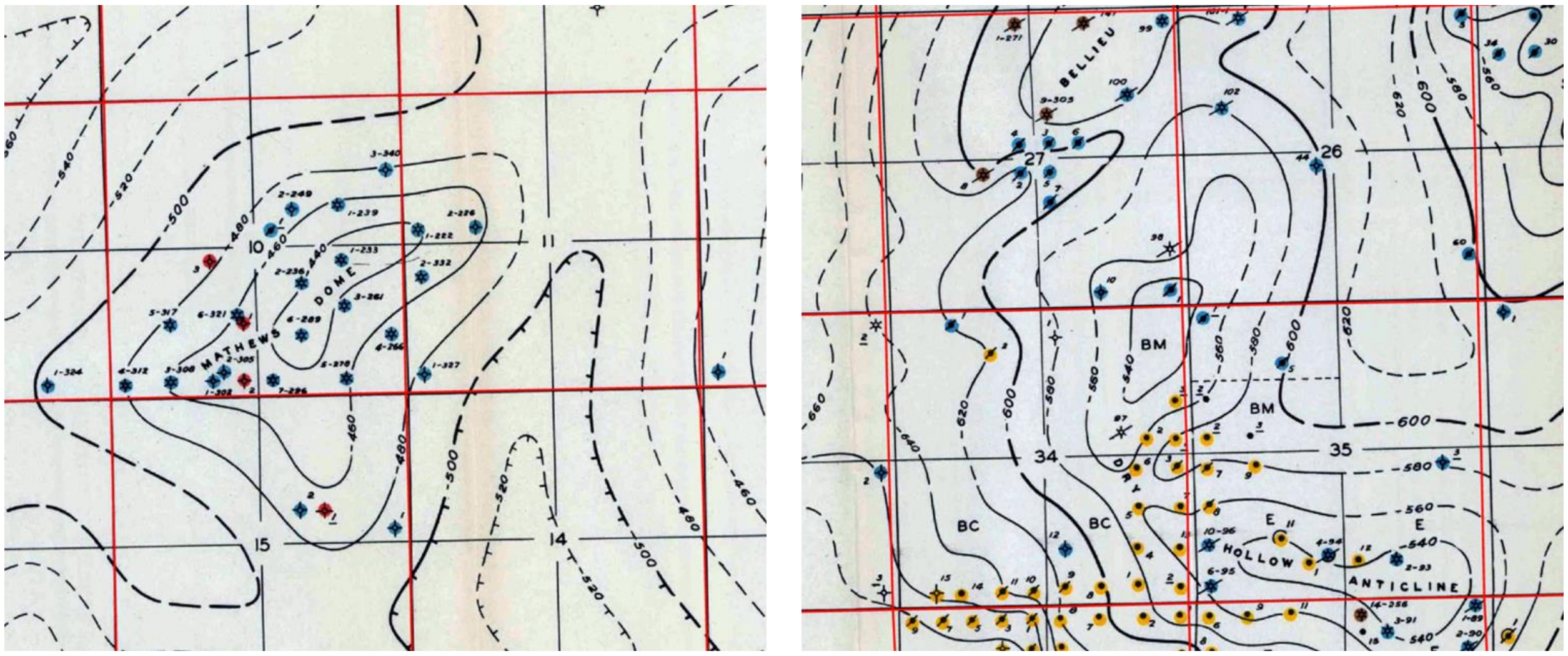

Figure S1: Representative regions from Plate 5 in Bulletin 900 showing errors in georeferencing. The errors are small in the region shown in the left panel and larger in the region in the right panel as shown by the difference in alignment of the red BLM-PLSS grid and the historical map's black PLSS grid.

C. Average ground sampling distance (m): The average size of a pixel in meters. This value reflects both the original map scale and the resolution used when converting the PDF maps to raster format.
D. Maximum error (m): Maximum georeferencing error in meters estimated based on visual inspection.
E. No. of detected wells: Total number of wells detected by the computer vision (CV) model for each map.
F. Legend detections: Number of CV-model based well detections that were part of the legend.
G. Final no. of wells after quality checks: Total number of wells from each map after removing incorrect detections
H. Estimate of missed wells based on visual inspection: Number of wells that were not detected by the CV-model but were found in the map upon visual inspection.
I. No. of expected wells from report: Number of wells mentioned in the report associated with the bulletin for each of the map. Note that the report associated with Bulletin 686 did not have much information about the total number of wells in each map.
J. Notes: Additional information about each of the maps.

S6. SUMMARY OF POTENTIAL UNDOCUMENTED WELLS

Additional information about the potential undocumented wells identified from Bulletins 686 and 900 is summarized in the spreadsheet “B686” and “B900” in the workbook es6c03981_si_002.xlsx. The columns in both spreadsheets specify the following information:

A. Well ID: Unique identifier assigned by the authors for potential undocumented wells identified in this study
B. Latitude: The latitude of the potential undocumented well detected by the CV model in the WGS 84 coordinate system
C. Longitude: The longitude of the potential undocumented well detected by the CV model in the WGS 84 coordinate system.
D. Map Name: Name of the map from which the potential undocumented well was identified. The name is a combination of the Report Name and Plate No. fields defined in section SS5.
E. Well Type: The status of the well based on the map legend.

S7. EXAMPLES OF PLUGGING RECORDS IN OSAGE COUNTY

Plugging records for three wells can be found in the files es6c03981_si_005.pdf, es6c03981_si_006.pdf, and es6c03981_si_007.pdf. The form template has a field for recording the details of the casing that was pulled out.

S8. ACKNOWLEDGEMENTS

This work was supported as part of the Consortium Advancing Technology for Assessment of Lost Oil & Gas Wells, funded by the Undocumented Orphan Well Program in the Office of Oil and Natural Gas within the Hydrocarbon and Geothermal Energy Office of the U.S. Department of Energy. This work was completed in part through funding from the Mickey Leland Energy Fellowship, Lawrence Livermore National Laboratory’s Data Science Summer Institute, and the Livermore Lab Foundation Fellowship Program. We also thank the people of the Osage Nation for providing access to their field sites. Parts of this work was performed under the auspices of the U.S. Department of Energy by Lawrence Livermore National Laboratory under Contract DE-AC52-07NA27344 and Lawrence Berkeley National Laboratory under Contract DE-AC02-05CH11231. The U.S. Government retains, and the publisher, by accepting the article for

publication, acknowledges, that the U.S. Government retains a nonexclusive, paid-up, irrevocable, worldwide license to publish or reproduce the published form of this manuscript, or allow others to do so, for U.S. Government purposes.

S9. DISCLAIMER

The geographical coordinates provided in this paper correspond to the locations of potential undocumented wells extracted from historical maps. The actual presence of wells need to be confirmed with on-the-ground investigations. For your safety, please do not attempt to visit or investigate these sites without appropriate safety training, proper equipment, and authorization from local authorities. Approaching these well sites without proper personal protective equipment (PPE) may pose significant health and safety risks. Oil and gas wells can emit hazardous gases including methane, which is flammable, odorless and colorless, as well as hydrogen sulfide, which can be fatal even at low concentrations. Additionally, there may be unstable ground near the wellhead that may collapse around the wellbore. This document was prepared as an account of work sponsored by the United States Government. While this document is believed to contain correct information, neither the United States Government nor any agency thereof, nor the Regents of the University of California, nor any of their employees, makes any warranty, express or implied, or assumes any legal responsibility for the accuracy, completeness, or usefulness of any information, apparatus, product, or process disclosed, or represents that its use would not infringe privately owned rights. Reference herein to any specific commercial product, process, or service by its trade name, trademark, manufacturer, or otherwise, does not necessarily constitute or imply its endorsement, recommendation, or favoring by the United States Government or any agency thereof, or the Regents of the University of California. The views and opinions of authors expressed herein do not necessarily state or reflect those of the United States Government or any agency thereof or the Regents of the University of California.

The authors declare no competing financial interest.

S10. REFERENCES

(1) *All Wells, GIS Mapping, California Department of Conservation*. https://www.conservation.ca.gov/calgem/maps (accessed 2025-08-08).

(2) *Well Database by Osage Minerals*. https://www.osageminerals.org/maps/f3e2c94e54d44b5bab8f591a02168c16 (accessed 2025-08-12).
(3) *Search - Well Records*. https://filerequest.conservation.ca.gov/wellrecord (accessed 2025-10-07).
(4) White, D.; Others. *Structure and Oil and Gas Resources of the Osage Reservation, Oklahoma*; U.S. Geological Survey Bulletin 686, 1922. https://doi.org/10.3133/b686.
(5) Bass, N. W. *Subsurface Geology and Oil and Gas Resources of Osage County, Oklahoma*; U.S. Geological Survey Bulletin 900, 1942. https://doi.org/10.3133/b900.
(6) ArcGIS Pro 3.4.0, 2024.
(7) *BLM Public Land Survey System (PLSS)*. https://hub.arcgis.com/maps/USGS::blm-public-land-survey-system-plss/about (accessed 2025-10-30).
