# Supplementary material for: Locating Undocumented Wells Using Historical Oil and Gas Exploration Maps: A Case Study in Osage County, Oklahoma
Source: Environ Sci Technol. 2026 Jul 16;60(30):20913–24. doi: 10.1021/acs.est.6c03981 (PMC13449896; doi:10.1021/acs.est.6c03981)
Supplement: Supplementary file 2 [file es6c03981_si_003.pdf]

DEPARTMENT OF CONSERVATION
DIVISION OF OIL, GAS & GEOTHERMAL RESOURCES
801 K Street, MS 20-22 Sacramento, CA 95814-3530
Phone:(916) 322-1110 Fax:(916) 322-1201

REPORT OF WELL ABANDONMENT

Sacramento, California
February 13, 2014

NOTE: To File.
(Unknown Operator)

,

Your report of abandonment of well **"Unknown" 1**, A.P.I. No. **013-20381**, Section **10**, T. **01N**, R. **04W**, **MD** B.&M., **--** field, **Contra Costa** County, dated **11/06/2013**, received 11/09/2013, has been examined in conjunction with records filed in this office. We have determined that all of the requirements of this Division have been fulfilled relative to plugging and abandonment of the well, removal of well equipment and junk, and filing of well records.

NOTE:

1. Site inspection made and approved on 11/18/2013.

Tim Kustic
State Oil and Gas Supervisor

Robert S. Habel
Robert S. Habel
District Deputy

MB:jc

OG159

DISTRICT 6 - WELL RECORDS CHECKLIST

API# 013-20381 **WELL ID:** Unknown 1

NOTICE FOR: ____drill ____ rd ____rw ✓ abd

P-number(s) 613-0081 & ________

RECORDS RECEIVED	Date Rec'd	Records Ok	NORD	121 entry	Remarks
Well Summary (OG100)					
History (OG103)	11/7/2013	✓			Entered in to CAIWims
Directional Survey					2/19/2014 JC
E-logs (2")					
E-logs (5")					
MUD Logs					
CBL					
Other					

____ **CONFIDENTIAL**

RELEASE DATE ____________

ABANDONMENT

✓ NEEDS Surface Inspection

____ MAP MADE for Surface Inspection

Surface Inspection COMPLETED by JAS/BGT

DATE 11/18/2013

✓ FINAL LETTER

____ FINAL LETTER NTS

Final Letter Completed by: _____ **Date** __________

STATUS

Producing-gas	____
Idle-gas	____
Abandoned-gas	____
Drilling-Idle	____
Abandoned-dry hole	____
Observation	____
Water Disposal	____
Other	✓

Date Work Completed 9/13/2013

Remarks Oil "well" in Wildcat Canyon Park

**RECORDS APPROVED**	**DIGITAL RECORDS APPROVED**
BY: JAS DATE: 11/19/2013	BY: bgt DATE: 12/10/2013

California Department of Conservation
Division of Oil, Gas & Geothermal Resources

Wildcat Canyon East Bay Regional Park
“Unknown” 1 Abandonment Report
API #013-20381

December 10, 2013

On Tuesday, June 21, 2011, Carol Victor, Assistant District Counsel for the East Bay Regional Park District (EBRPD or Park) contacted B.G. Tackett, Associate Oil and Gas Engineer for the District 6 office of the Division of Oil, Gas and Geothermal Resources (DOGGR) to follow up on an unknown well found by the Park in Wildcat Canyon Regional Park that was leaking an oily substance to the surface (Figure 1), Photo 1.

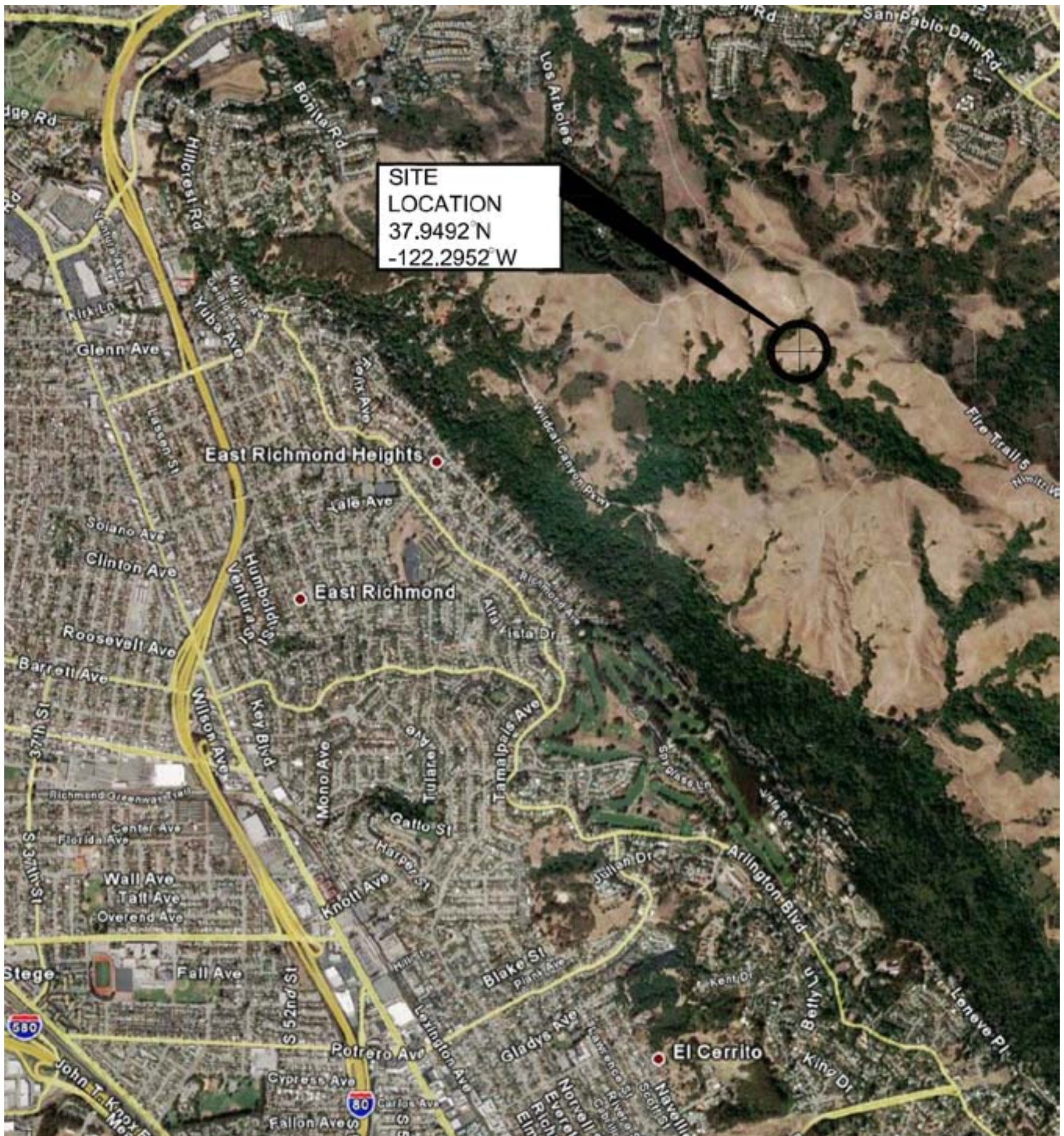

Figure 1. Site Location

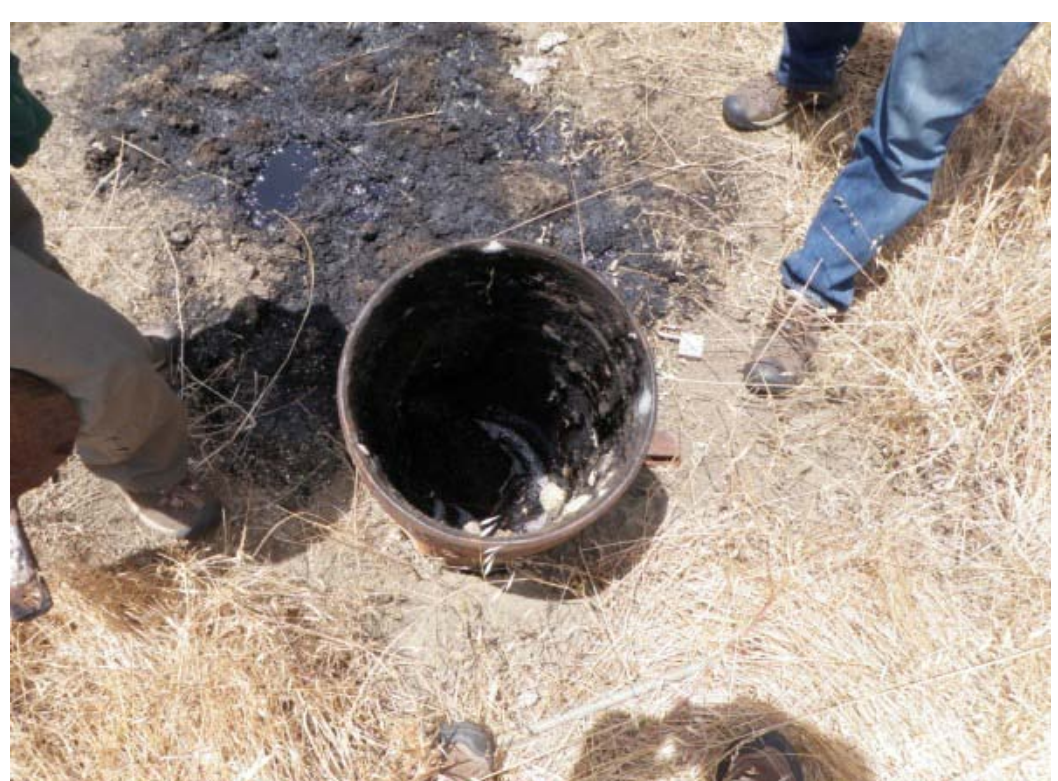

Photo 1. The leaking well

One year previously, and at the request of DOGGR District 6 staff, the Park hired the consulting firm of Fugro West, to assess the situation and pump out the standing liquid in order to help make a determination as to whether the oily substance was in fact crude oil. With no free water observed, 100 gallons of the substance was pumped from the well for analysis. In addition, there was localized staining and saturated soil extending 4 feet wide by 14 feet down slope (Photo 2). The Park District removed the most heavily saturated soil and placed a rubber seal around the presumed surface casing to prevent any further leaking over the winter of 2010-2011. The Park's Water Resource Manager, Matt Graul, visited the site in February and said the liquid had recharged to approximately 5 feet below the level first observed before any liquid was pumped out. Resulting from the recharge of liquids, the presence of the well casing at surface, and a full initial site investigation performed by Fugro West, Appendix 1, the consultant who helped with the preliminary investigation concluded that the well was in fact an oil well. DOGGR had no record of such a well ever having been drilled.

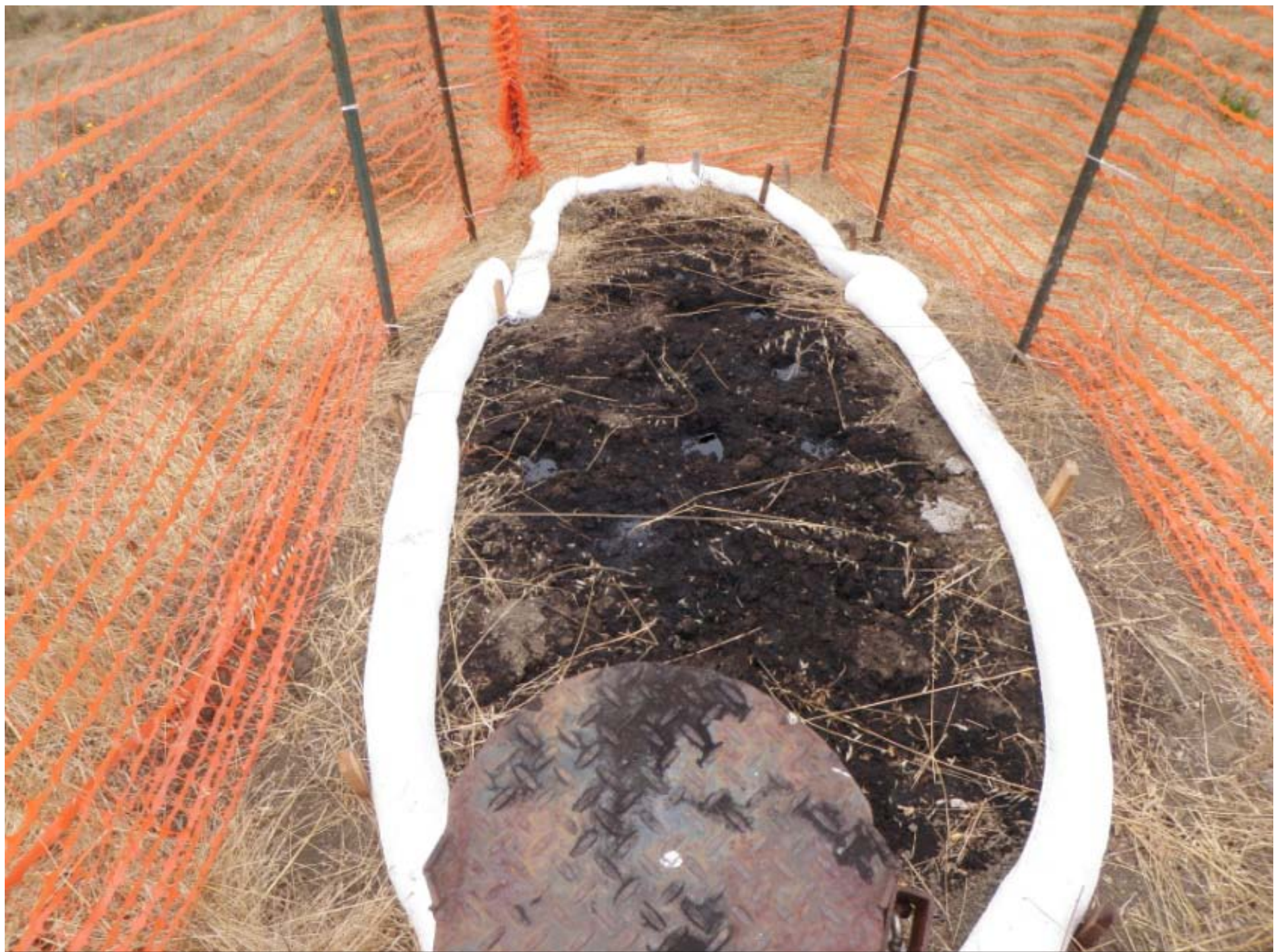

Photo 2. Oily substance and soil contamination below the surface casing of the unknown well. Note wildlife/cattle exclosure fencing and absorbent wattle in place.

Now that contact had been reestablished with DOGGR, Ms. Victor then requested DOGGRs assistance in determining next steps and requested funding assistance from DOGGRs orphan well program in order to properly abandon the well.

With winter approaching, and realizing that there was no way to move equipment in and out before the rains came, DOGGR staff performed an initial site investigation with staff from the Park, Photo 3.

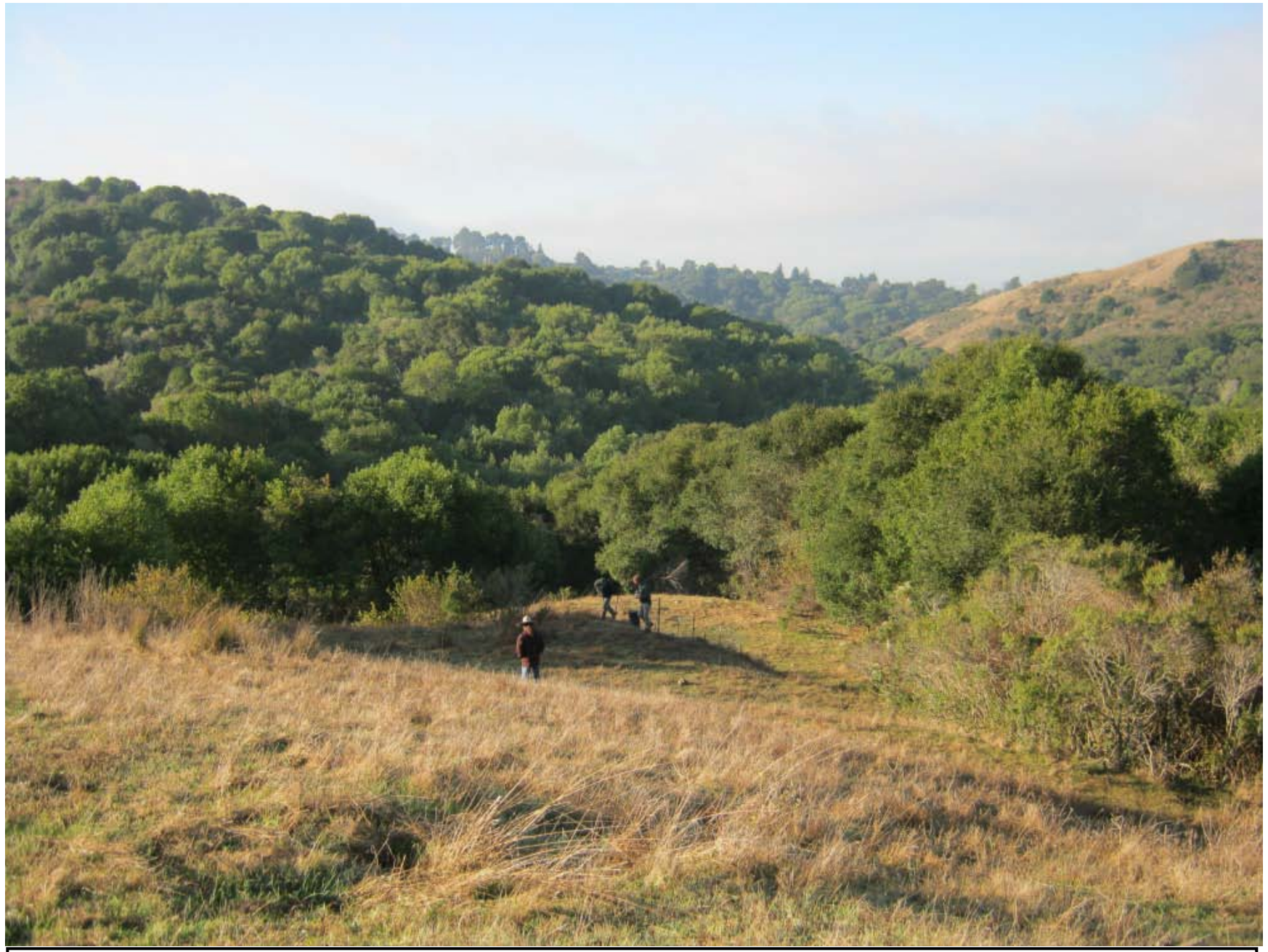

Photo 3. The "Unknown" 1 well location in beautiful Wildcat Canyon Regional Park

During winter 2012-2013 funds were made available through DOGGRs orphan well fund and on April 27, 2013, DOGGR staff and interested abandonment contractors walked the site. During this visit it became apparent, that while not inaccessible, moving rig equipment onto the location would present its own set of challenges including road grading, Photo 4.

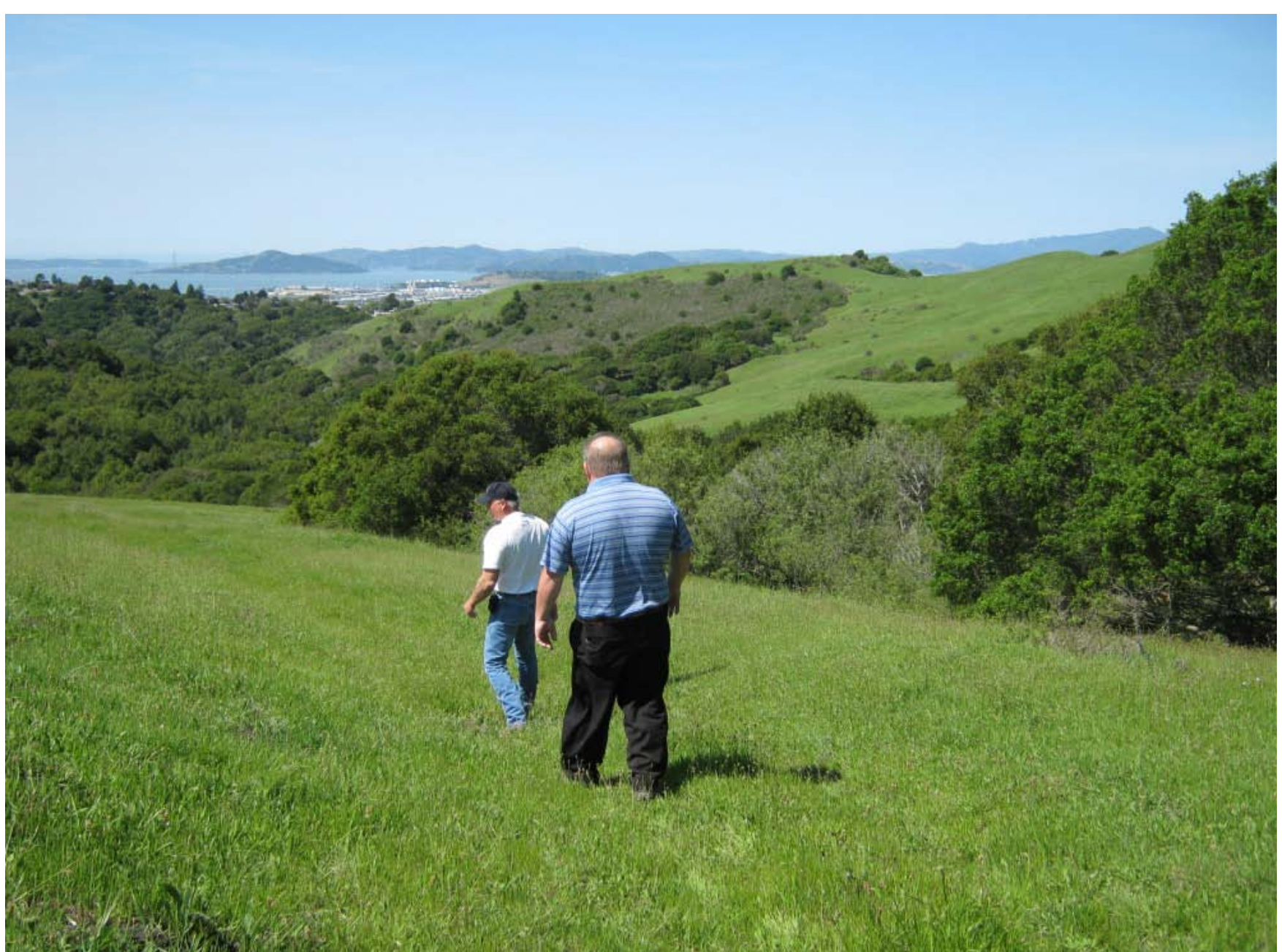

Photo 4. Contractor site inspection April 27, 2013

After contract issues between DOGGR and the Department of General Services were finalized work finally began on Thursday, August 22, 2013. District 6 Energy and Minerals Resources Engineer, Joy Silva, arrived at Wildcat Canyon Regional Park to begin the state-contracted abandonment work of "Unknown" 1, and met with Consulting Wildlife Biologist Amanda Murphy and Park fire crew at park headquarters located at 2500 Mc Bryde Avenue, Richmond, Contra Costa County, California. Rival Well Services crew and equipment arrived on site at 1400 hours, at which time biologist Murphy held an informational meeting with the crew regarding the endangered species that are known to be present in the area of the well. Fire crew discussed fire control protocols at and around the well site. Photos 5-6. Biologist and fire crew were present for the duration of the abandonment program.

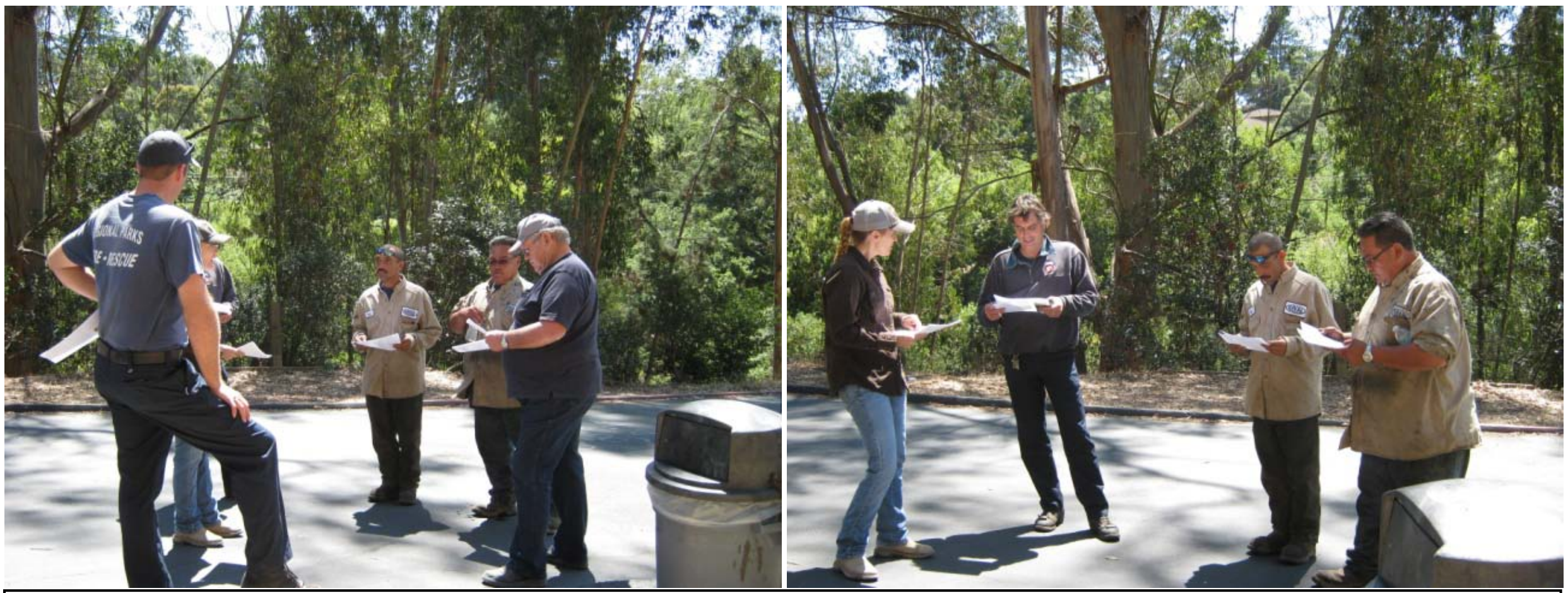

Photos 5-6. Rig crew, fire crew, and wildlife biologist Amanda Murphy at job pre meeting

After the meeting, work over rig and ancillary abandonment equipment was relocated to the well site, Photos 7-10.

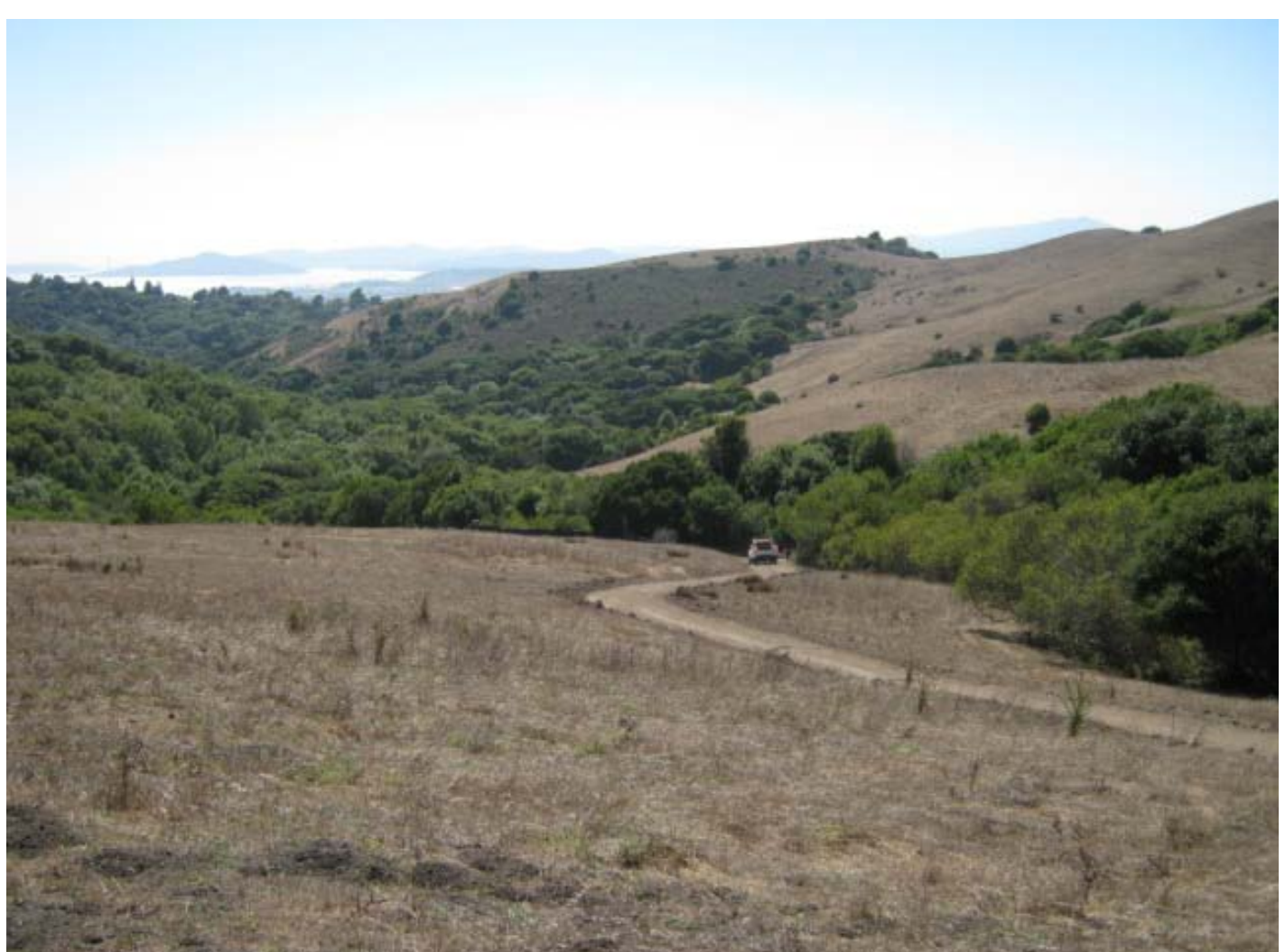

Photo 7. The newly graded road to "Unknown" 1

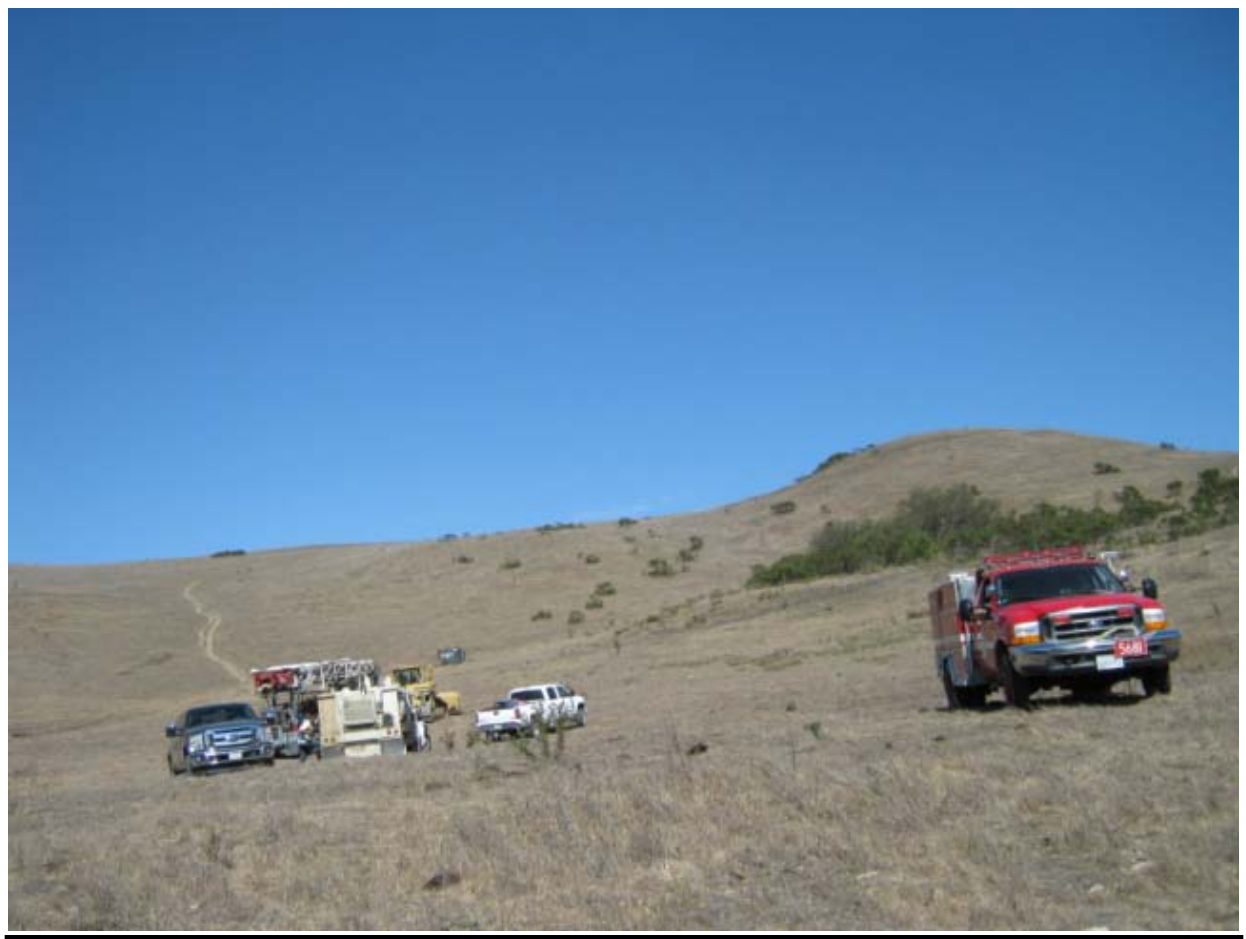

Photo 8. The ‘Parking Lot’ above the well site

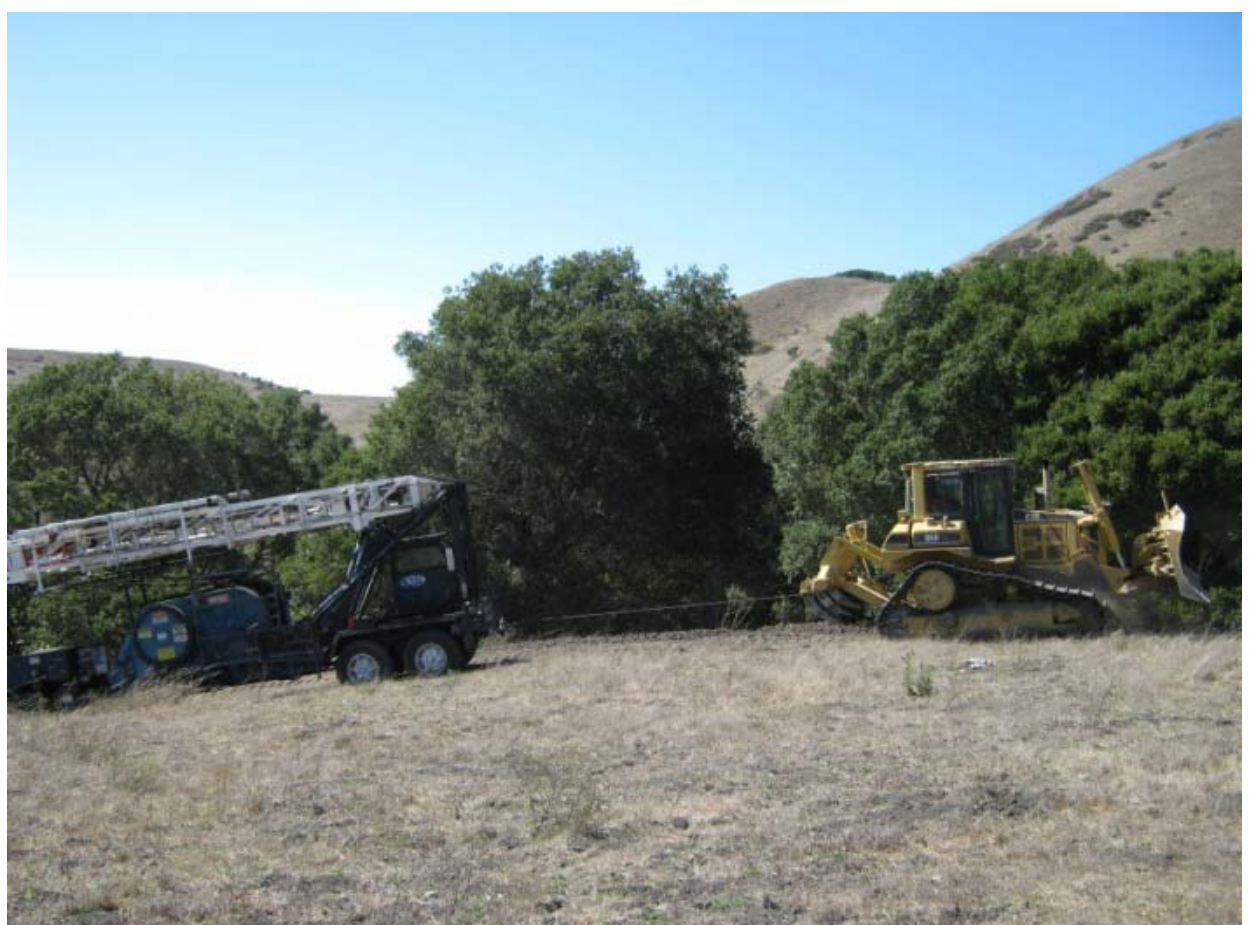

Photo 9, Dozer assisted rig move

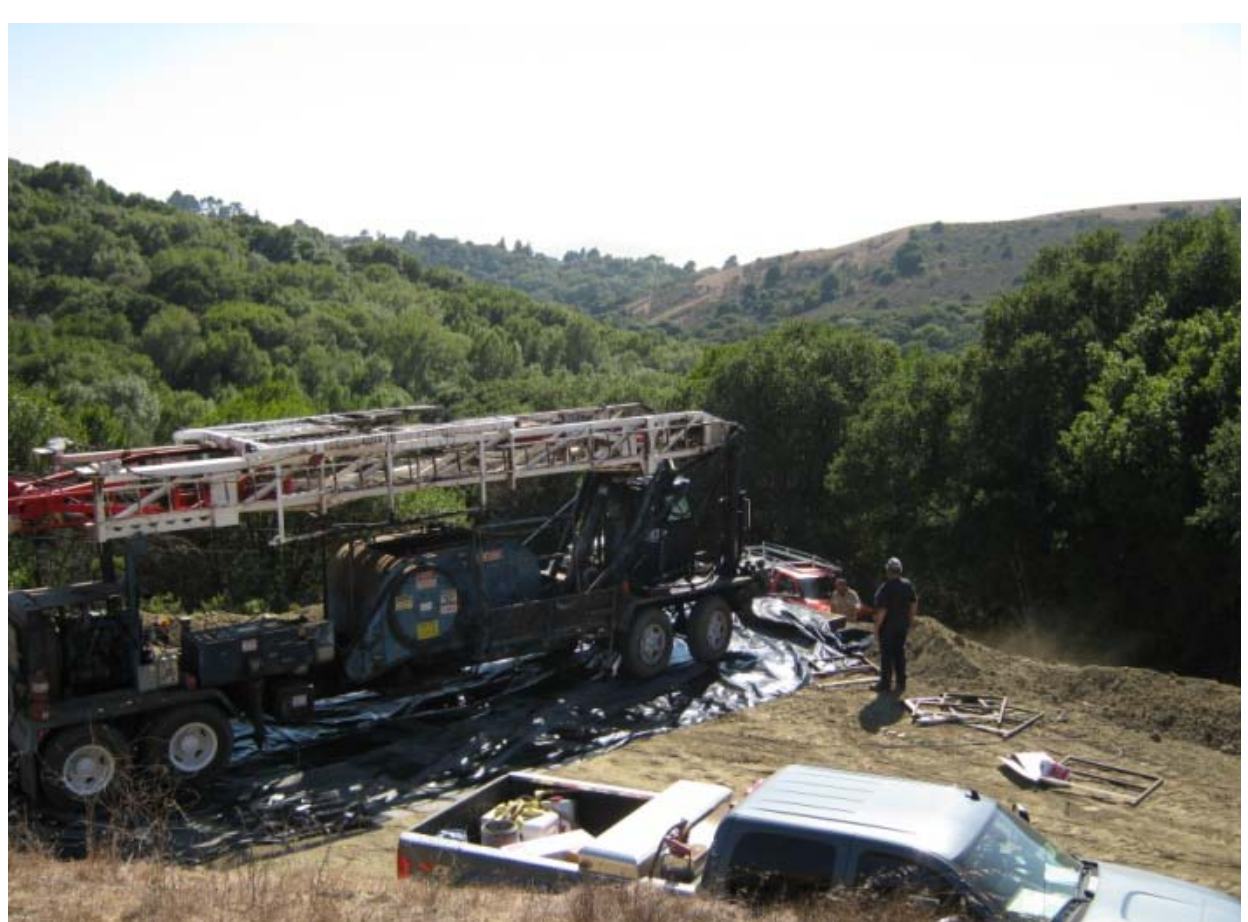

Photo 10. Rig in place. Note the heavy plastic sheeting placed below the rig to catch any leaks or spills from equipment.

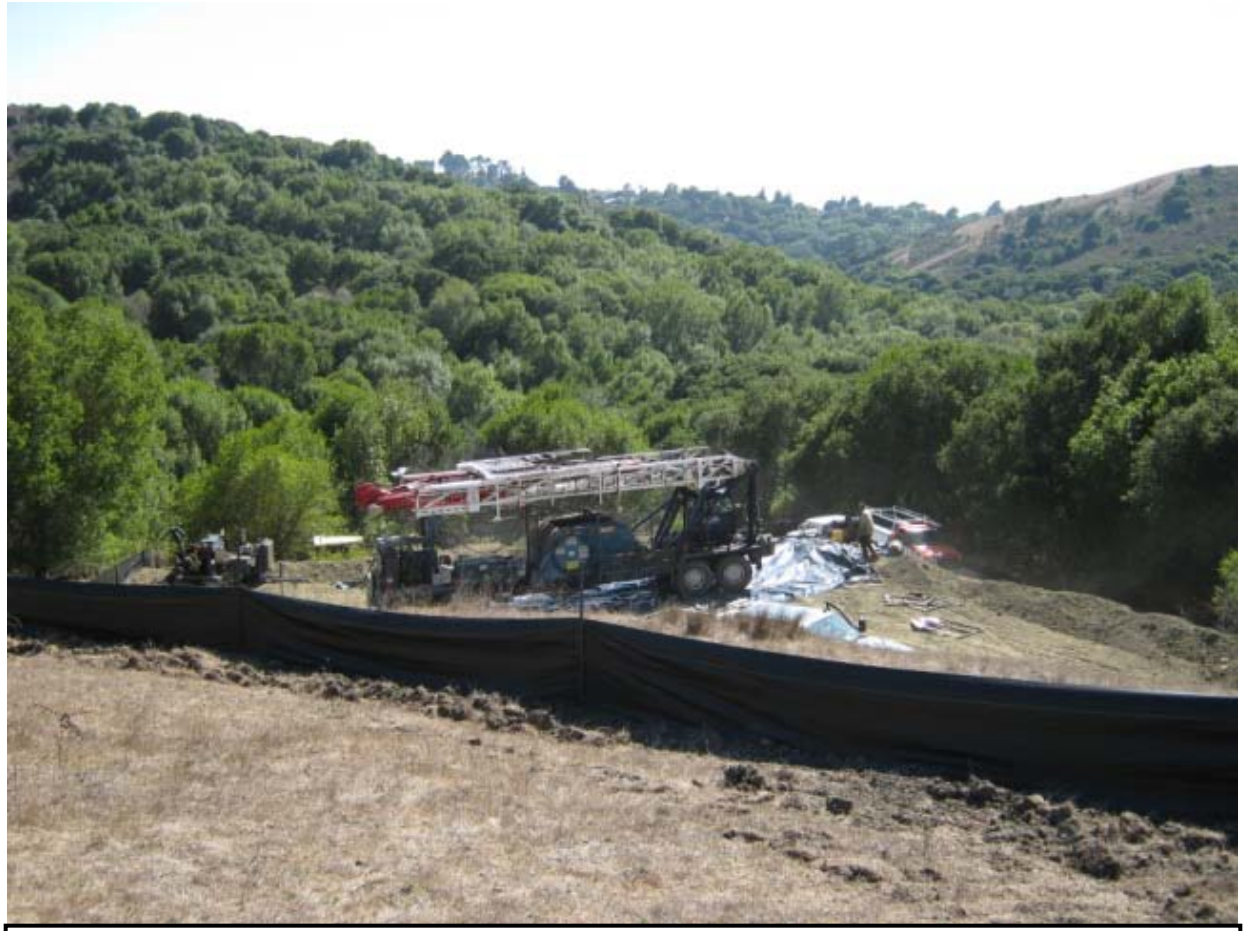

Photo 11, Rig on location. Note the black perimeter "snake fencing" installed for the protection of indiginous species.

Once the rig was stabilized on the well site, excavation of the "wellhead", a 12" conductor pipe wrapped in rubberized material secured with clamps, began, Photo 12. True to reports from the Park, oil was once again observed approximately 4' below the top of the conductor, Photos 11-14.

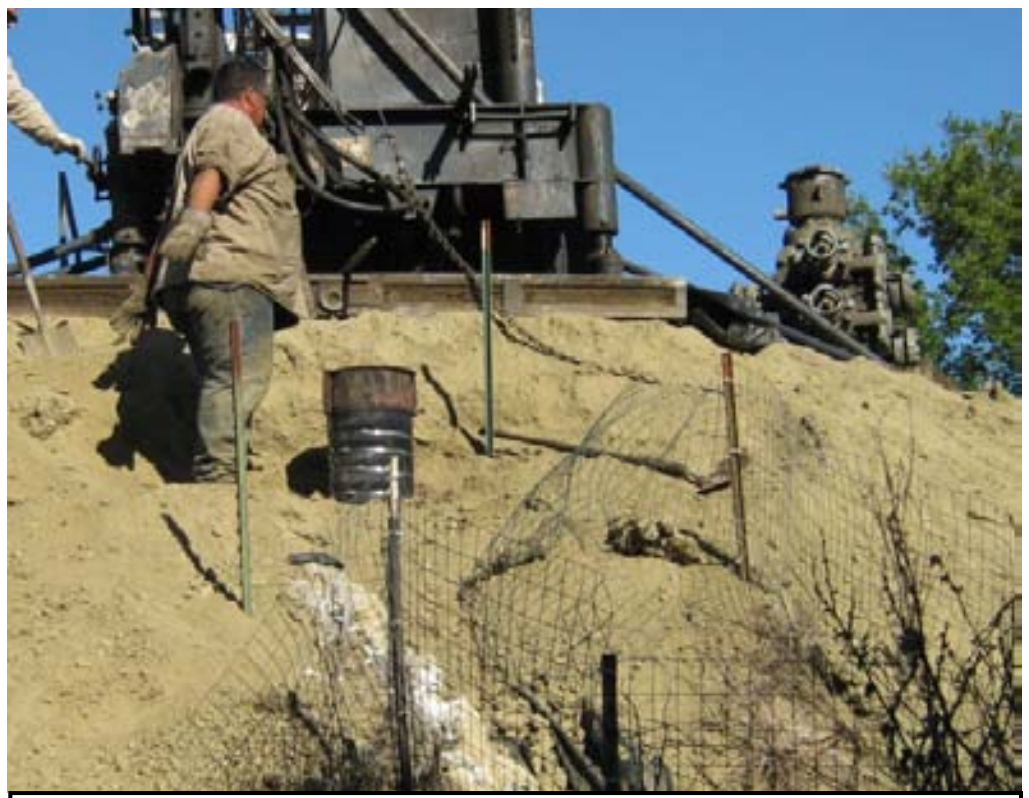

Photo 12. "Unknown" 1 well head

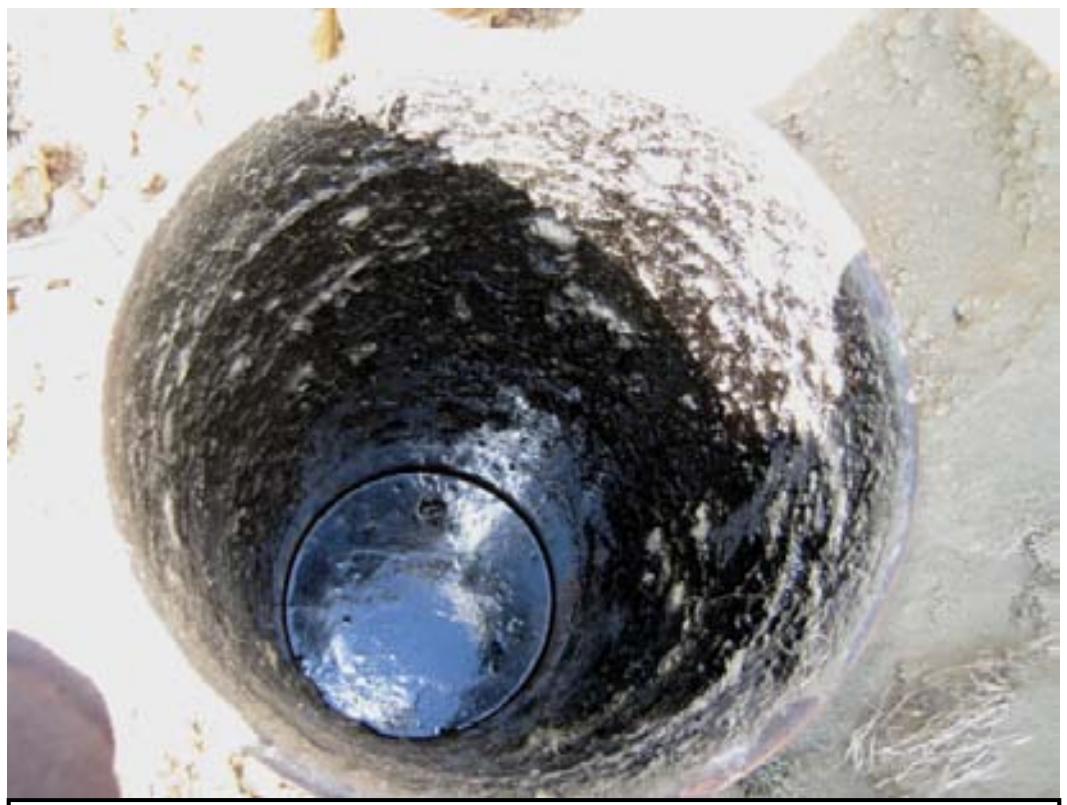

Photo 13. Oil in the well bore

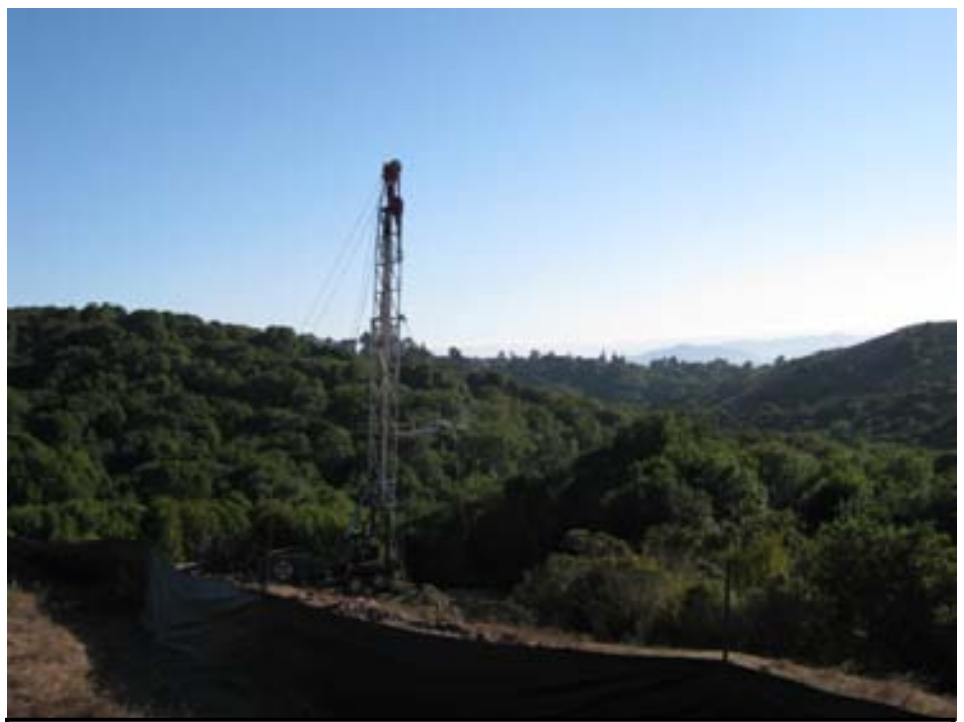

Photo 14. The derrick is finally up and ready to go to work

Friday, August 23, 2013

With a plan in place to excavate below the wellhead in order to collect any oil that might flow out during operations, Rival Well Services (Rival), the abandonment contractor, sent for a portable tank in which to collect oil from well bore. The rubber wrapping was removed from the conductor and it was noted that the pipe was shot through with bullet holes. Apparently the wrapping had been added in the past to prevent leakage. The crew hoped that a welder could weld the bullet holes closed, but there proved too many to repair. As such, the conductor was cut off and replaced with new pipe. Photos 15-18.

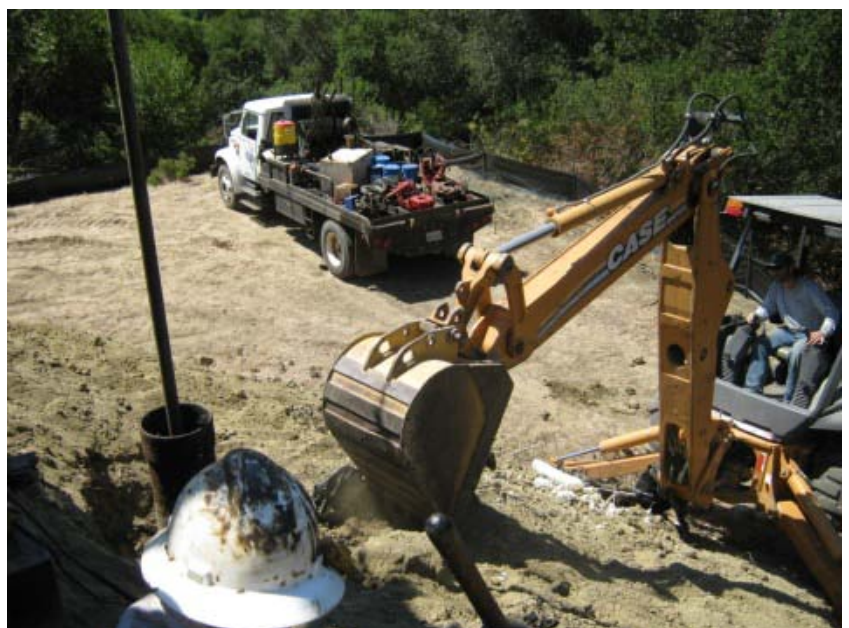

Photo 15. Excavating for oil collection

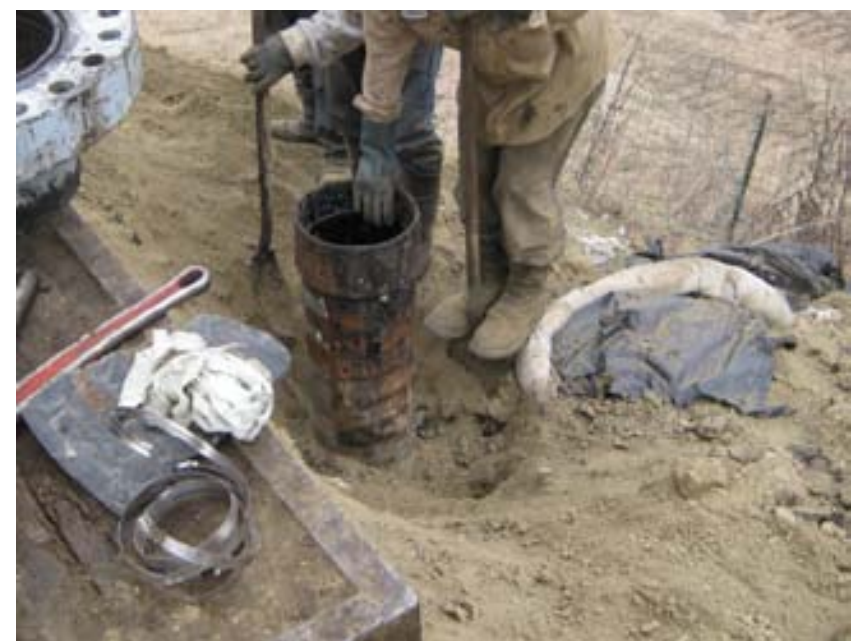

Photo 16. Assessing the conductor

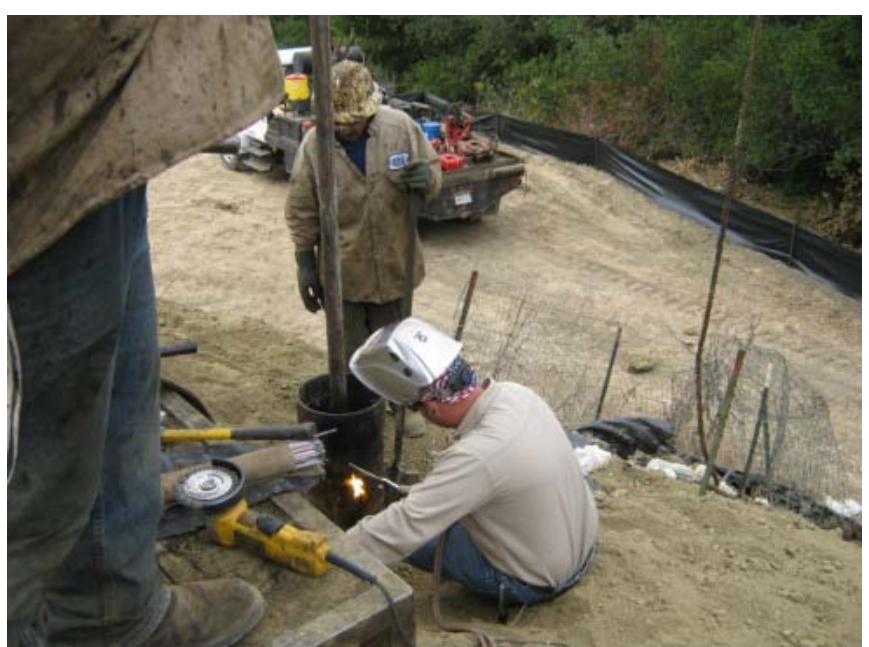

Photo 17. Attempt to repair bullet holes.

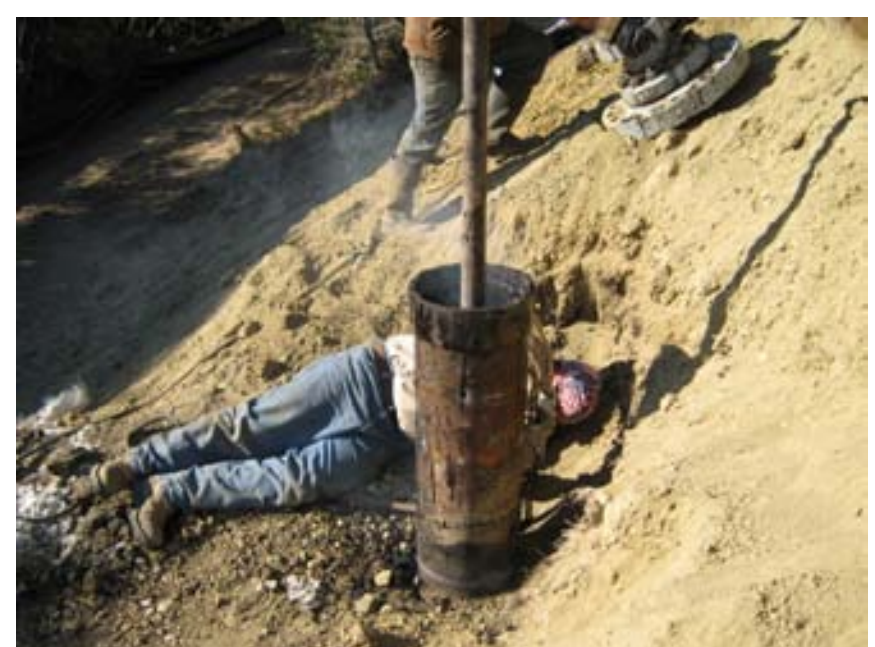

Photo 18. Cutting off the bullet riddled conductor

Saturday, August 24, 2013

Though a short work day for the crew, abandonment operations continued. The welder finished welding the conductor, the API flange and blow out prevention equipment (BOPE) were installed, and hydraulic control lines were connected. Once all of this was in place the rig floor was swung into final position, Photos 19-22.

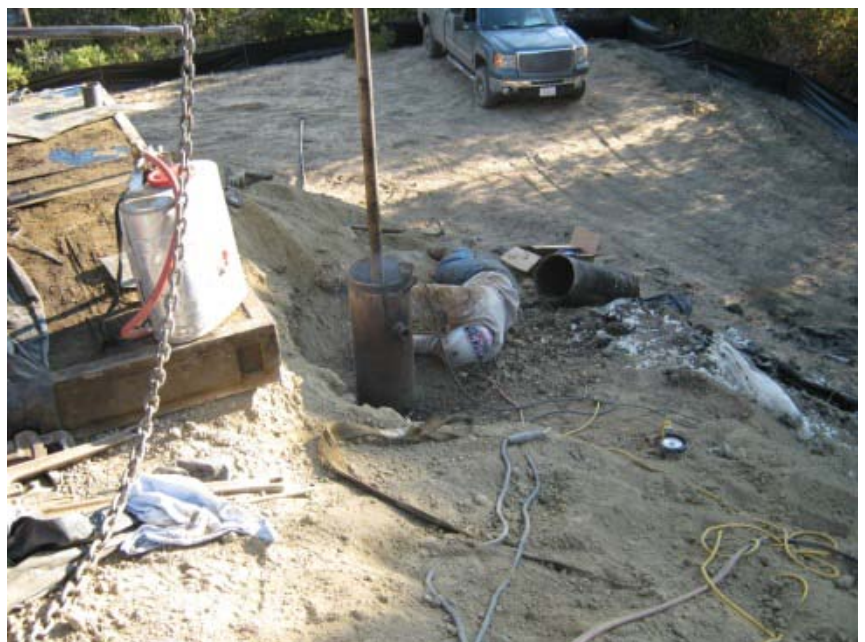

Photo 19. Installing the new conductor. Note the overflow nipple.

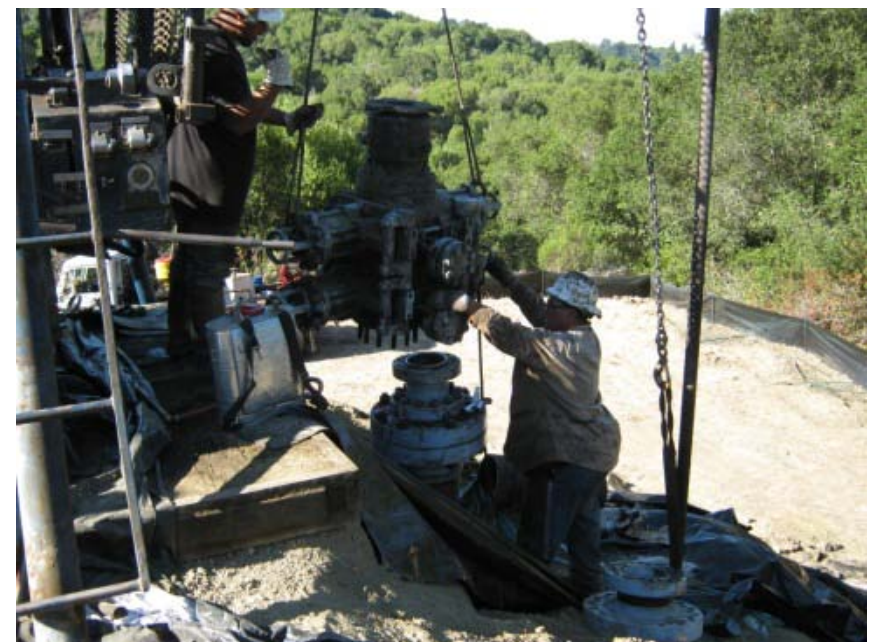

Photo 20. Installing the BOPE on the API flange.

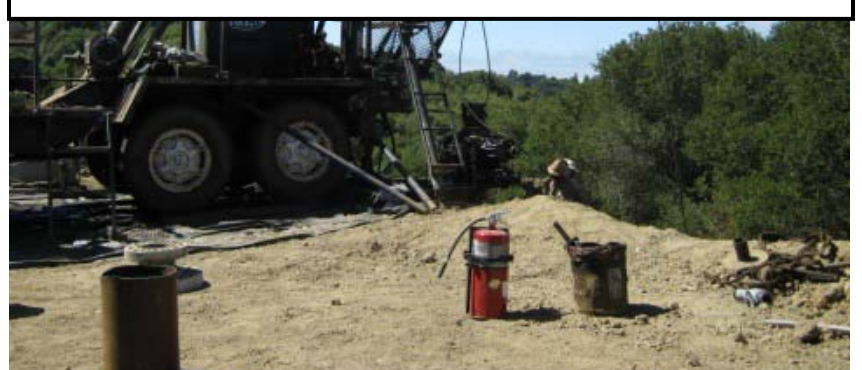

Photo 21. Placing the rig floor.

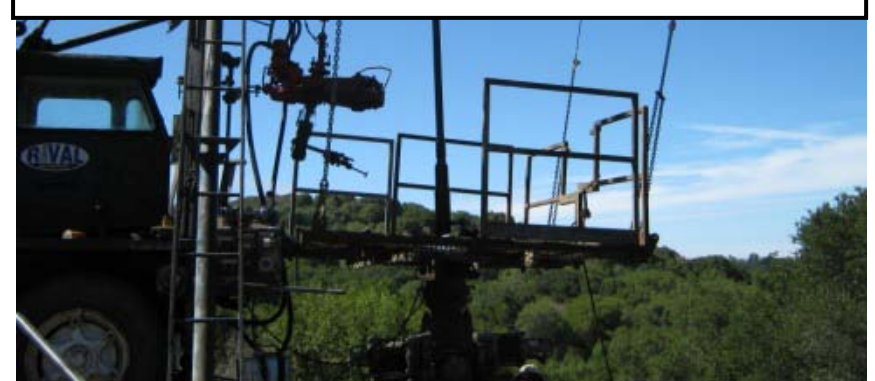

Photo 22. Rig floor in final position.

Monday, August 26, 2013

Back from days off in Bakersfield, the rig crew returned but unfortunately could not begin abandonment operations. Due to loosely compacted soil around the well head the crew had to rig down and excavate around conductor in order to install a 4' x 8' shoring box on the slope in order to properly support the rig and reset the rig support deck. Photos 23-25.

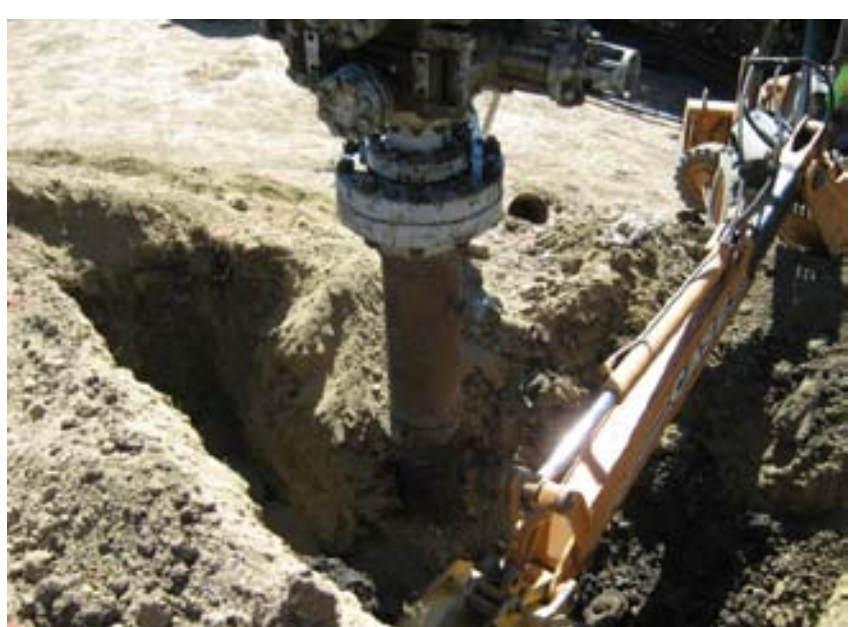

Photo 23. Backhoe excavating loose soils from the wellhead

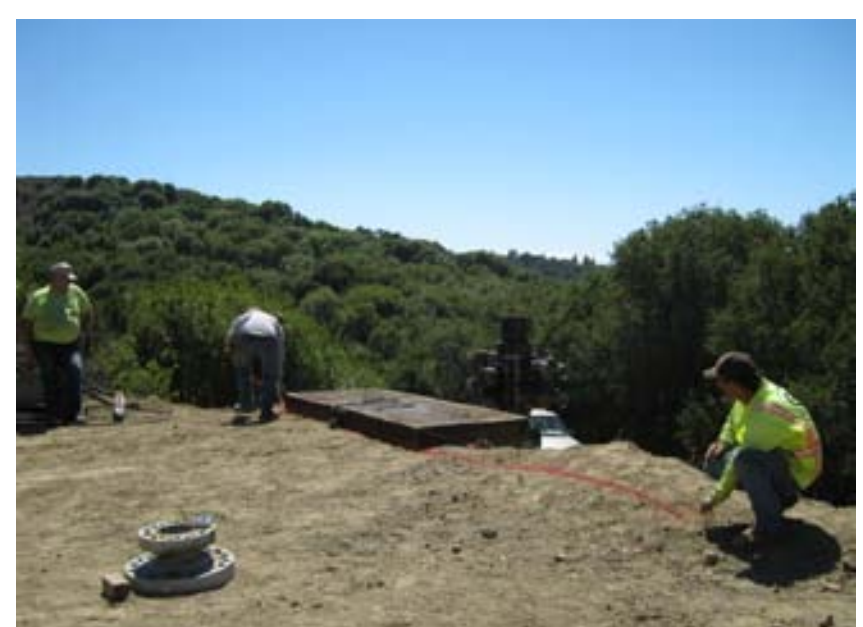

Photo 24. Crew resetting rig support deck

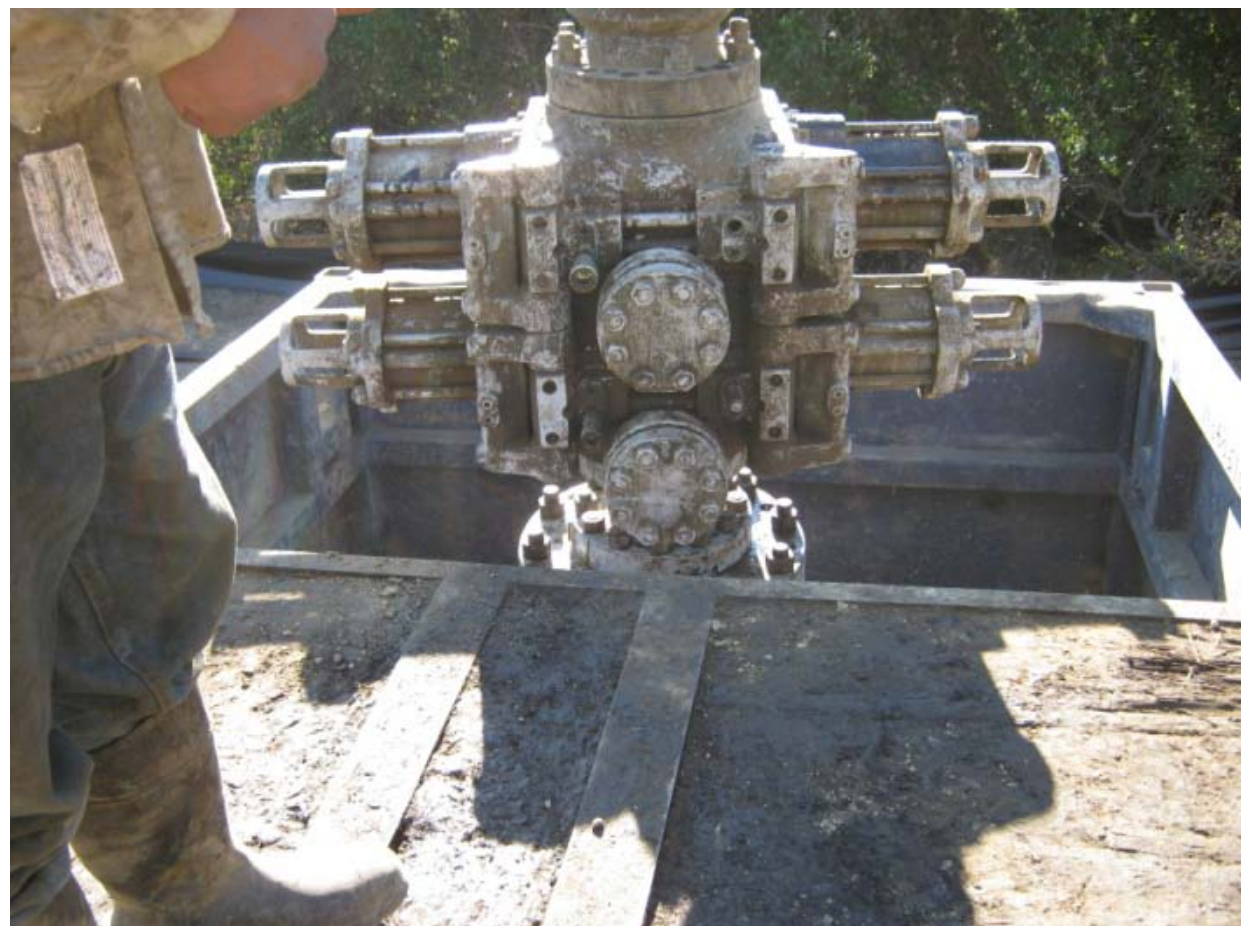

Photo 25. Support deck reinstalled with shoring box as support

Tuesday, August 27, 2013

After rigging back up over the hole, Rival ran in with a drill bit attached to two joints of tubing and began work when the tubing became plugged. Upon investigation, a 2” square x 4’ long piece of wood was discovered floating in the well bore, Photo 26. As drilling progressed more wood “findings” were circulated out of the well, Photo 27. Typical of old abandoned wells, this well was apparently plugged and abandoned with a redwood log driven into the well bore. This prompted Rival to order a larger power swivel and large mill bit to work through the wood. Total footage for the day was 101’.

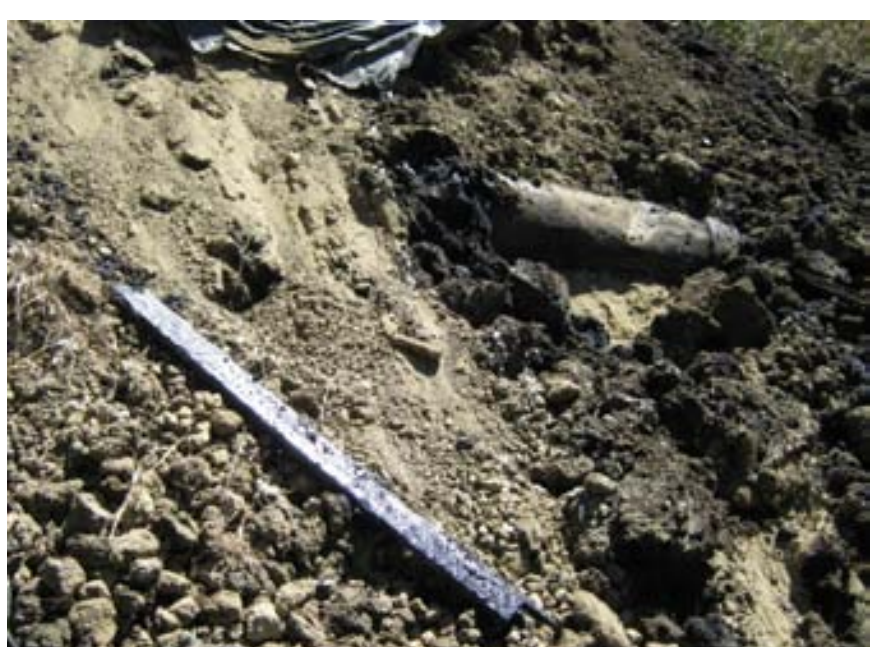

Photo 26. The initial stick that came from the well bore

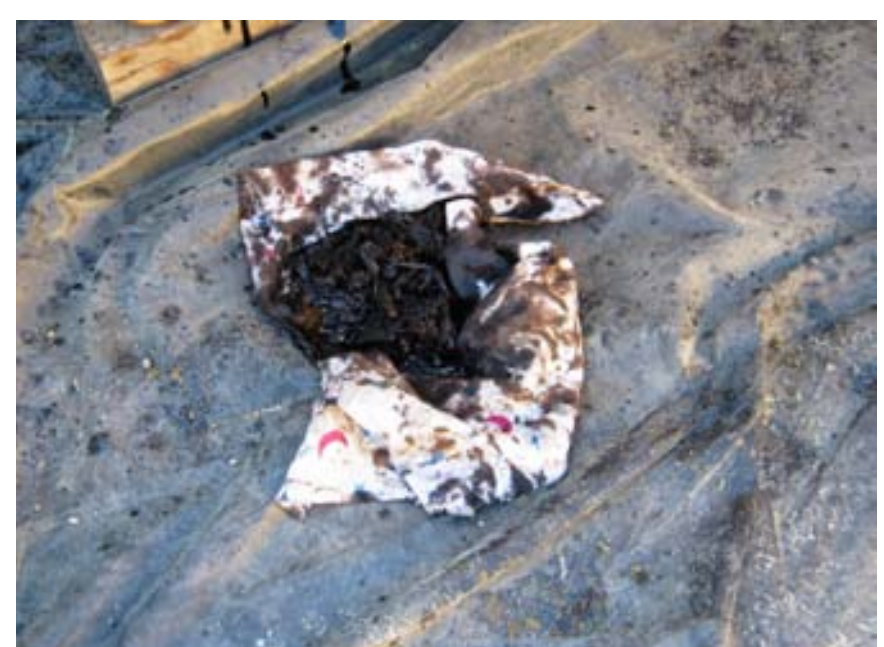

Photo 27. Oil soaked pulped wood “findings” circulated from the well

Wednesday, August 28, 2013

With Mary Bottoms, District 6 Energy and Minerals Resources Engineer, now on site, Rival continued operations at 101’ using a 4” mill; milling was going very slow. At 1500 hours the bit reached a depth of 115’-120’. Milling through wood, the wood fibers in the returns would occasionally plug the casing valve, interrupt circulation, and require cleaning of the valve, Photo 28. Using water to circulate the well bore, approximately 4-8 bbls had overflowed into the shoring box due to the clogged valve and the onsite holding tank was already approximately 2/3 full. Making the Rival job foreman aware that available storage tanks were getting critically full of evacuated fluids, an additional tank was ordered.

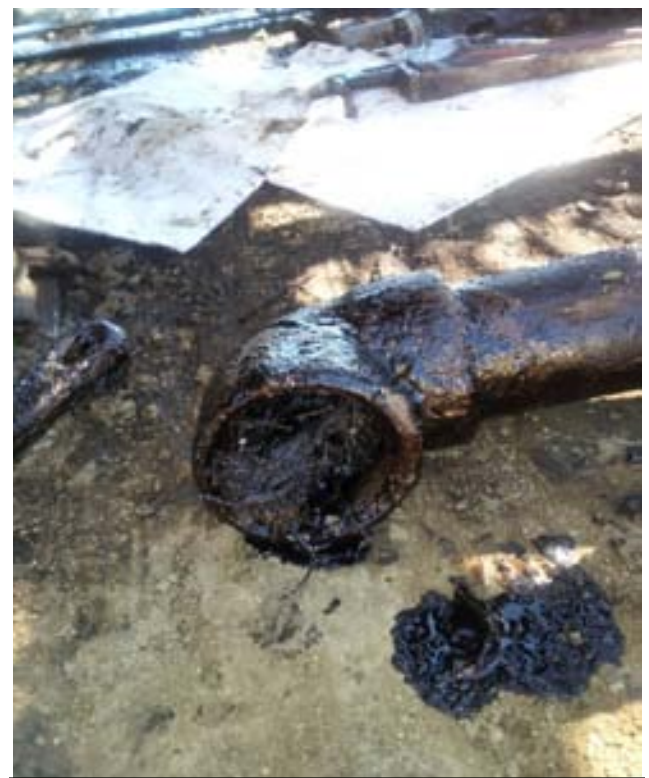

Photo 28. Wood clogged casing valve elbow

Thursday, August 29, 2013

Milling continued from 121' - 130' with little total hole made on the day. The casing valve & return line continued to clog with wood fiber and no progress was made in the afternoon due to a bit and drill pipe change in order to provide more weight in the drill string. DOGGR representative Alex Mirazi was on site.

Friday, August 30, 2013

Even though the casing valve and return line continued to clog with wood fiber, progress was made by drilling and by pushing the wood plug down hole from 131' to 300'. Through operations to date approximately 50 bbls of oil and water had accumulated in the mud pits and wash tank which proved challenging to remove as trucking companies with the necessary permits to haul crude oil were difficult to find in the local area. Ultimately, removal of the oil water mixture was arranged for following the Labor Day holiday weekend.

Saturday August 31, 2013 through Monday, September 2, 2013 – Labor Day – Holiday, No Work

Tuesday, September 3, 2013

Rival crew and DOGGR representative Mary Bottoms arrived at the Wildcat Canyon Regional Park at 1015 hours where they were met by biologist Mandy Murphy and were escorted to the well location. Waiting for the backup tank to be delivered until 1445 hours, the Rival foreman decided to move water from the holding tank to rig tank in order to begin milling operations, Photo 29. Milling from 1445 to 1645 hours, operations only made one foot of progress. However, upon delivery of the needed tank, Rival continued milling and reached a depth of 380' by 1800 hours.

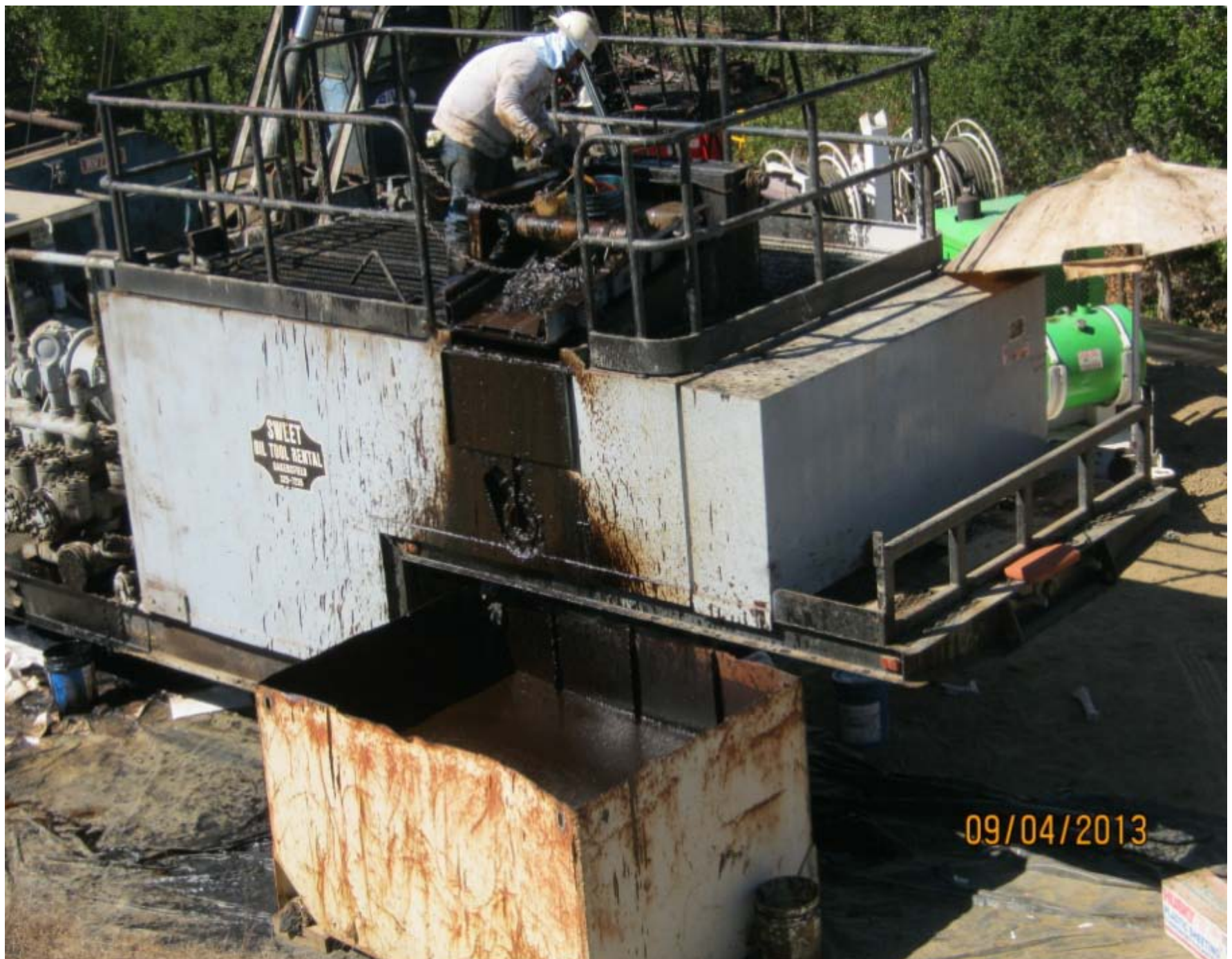

Photo 29. Rig and wash over tanks reaching capacity. Note the oil saturated wood drill cuttings on the shale shaker.

<u>Wednesday, September 4, 2013</u>

The Rival crew began milling at 365’ and made it to 443’ by 1115 hours, at which time they ran out of water. At 1400 hours water arrived, however progress stopped as the mill began spinning on the wooden plug. The mill was removed to find that it was rounded off, Photo 30. A new mill was installed, drill pipe run back in the hole, and milling was resumed at 1545 hours though at a reduced depth of 380’. A fluid storage tank was delivered and set on the lower pad before operations were shut down for night, Photo 31.

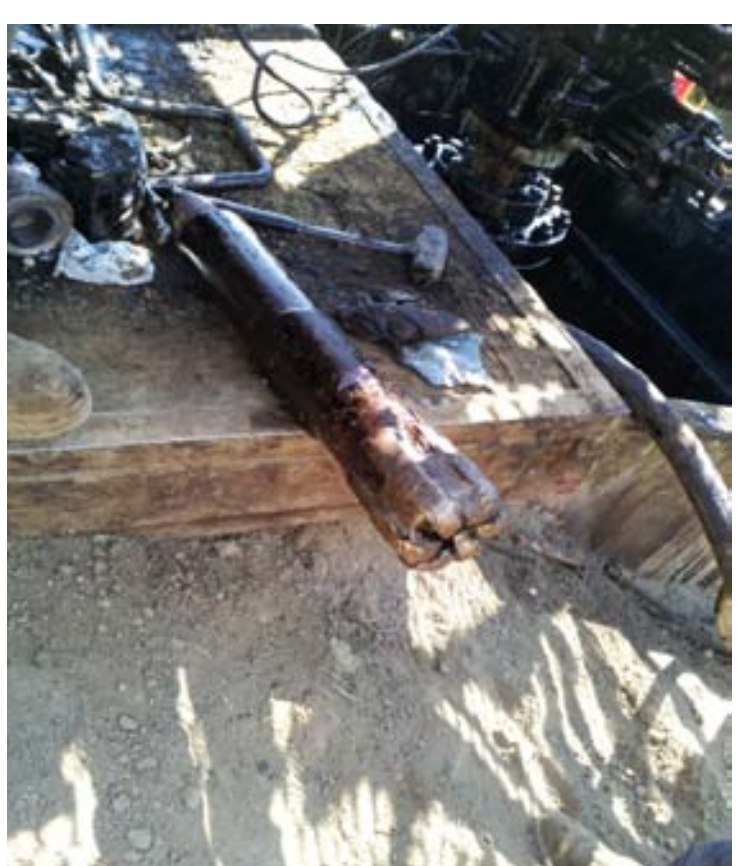

Photo 30. Rounded off mill

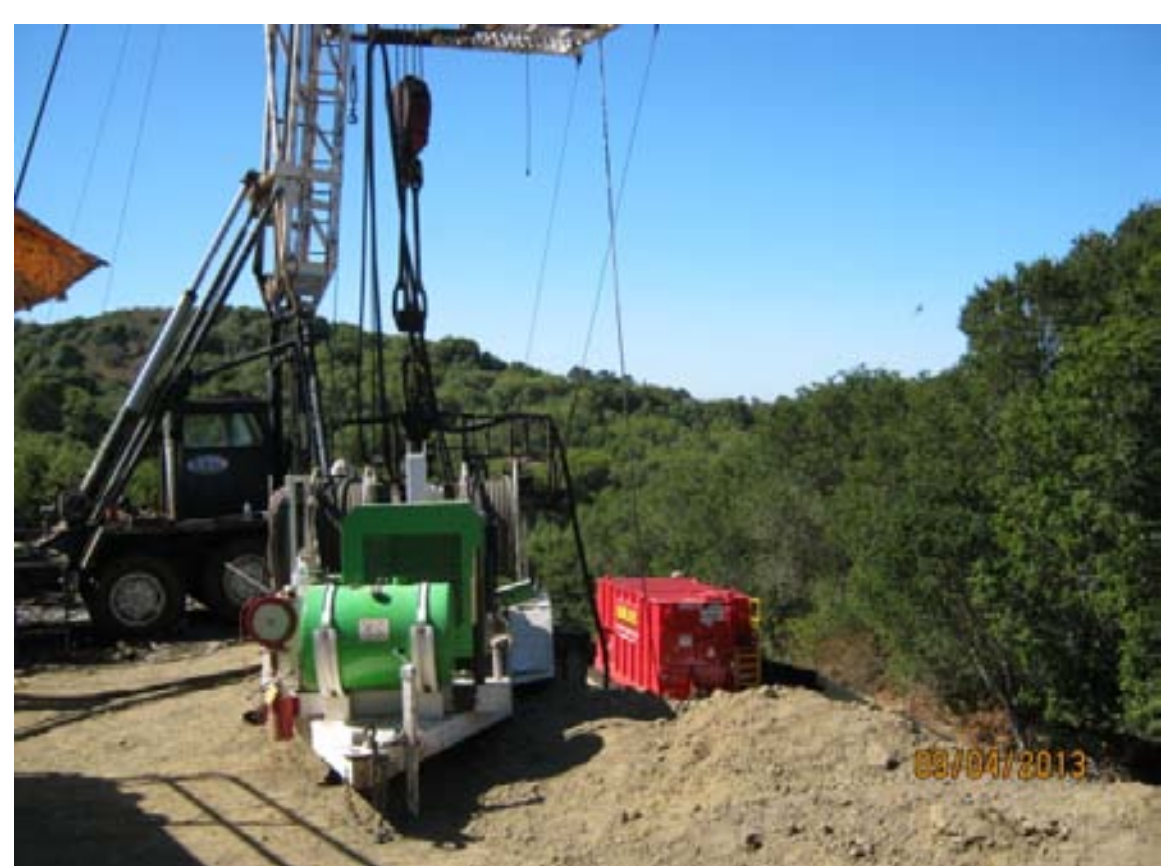

Photo 31. Fluid storage tank (red) delivered to well site, lower pad.

Thursday, September 5, 2013

Milling operations continued and a depth of 507' was achieved at 1115 hours. Continuing operations halted at 1400 hours due to lost circulation which was ultimately attributed to a clogged pump valve. The Rival crew pulled apart the pump valve and cleaned out wood cuttings, Photo 32. Returning to milling after repairs, the final depth reached on the day was 538'.

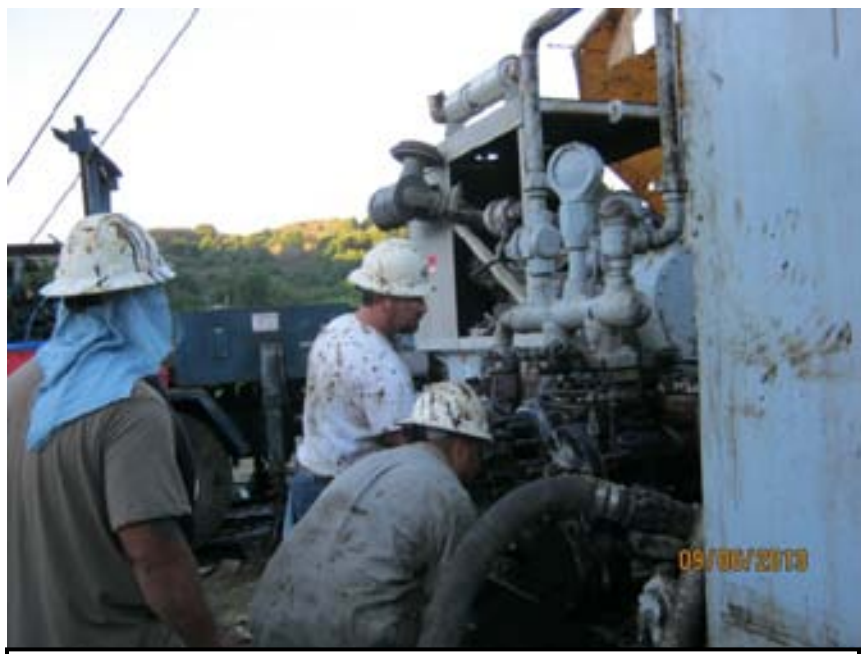

Photo 32. Rival crew working on the clogged pump valve.

Friday, September 6, 2013

Once again, the crew was having circulation problems and upon an internal check found wood cuttings lodged in the pump. Clearing the pump, milling began again at 1015 hours and depth of 522' was achieved by noon. Wood cuttings from the wood plug present from the original well abandonment continued to be a problem throughout operations, Photos 33-34.

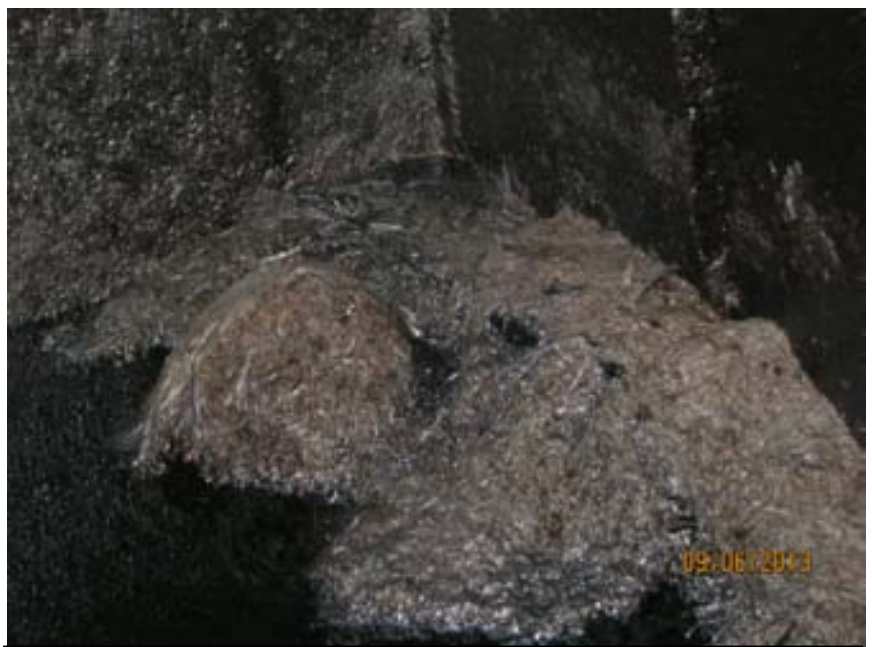

Photo 33. Wood cuttings settled in the holding tank. Mixed with oil, wood in the returns was an ongoing problem throughout the job.

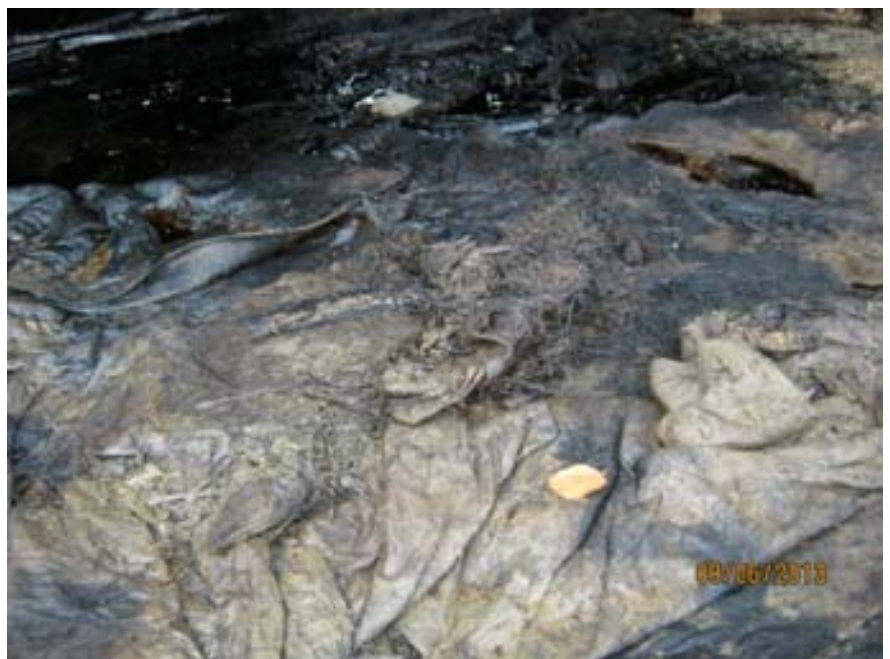

Photo 34. Wood cuttings removed from the pump.

Monday, September 9, 2013

DOGGR and all abandonment crew arrived at the park at 1030 hours for the daily biologist escort into the well site. Also present was Mike Woods, District Deputy – District 6, Bob Grayson, Rival Well Services, and Paul Williams, Rival site foreman. Per DOGGR staff, the water truck driver present was advised by Rival to bring only one half load of water which proved inadequate. Milling to a depth of 507', the saw tooth collar now being used plugged when water ran out at 1230 hours. Upon asking the Rival foreman if

there had been a misunderstanding regarding water volume to be delivered, DOGGR staff was told “no”. At 3:00pm, a replacement water truck arrived. At this time a discussion was had between, Mike Woods, Bobby Grayson, and Paul Williams, about setting a cement plug at the day’s lowest depth of 522’. Rival then began to move in equipment and condition the wellbore for cementing operations to take place the following morning, Photo 35. During the biologist’s final daily site check and species clearing process she discovered an oil release in an ephemeral creek located to the west and below the well. The spill was reported to the Office of Emergency Services that evening per Park protocol.

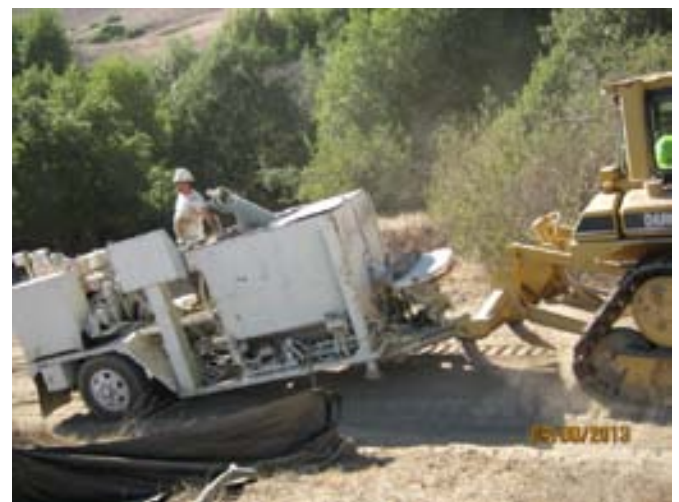

Photo 35. Cementing equipment on the move

Tuesday, September 10, 2013

DOGGR field engineer Joy Silva arrived on site for the morning escort to the well where she met with Wildlife Biologist Amanda Murphy and was informed of the aforementioned spill for the first time. Located just below grade of the lower access road and approximately 100’ northwest of the well bore, a mixture of mud and oil had migrated subsurface from the wellbore to a previously nonexistent surface expression which then flowed into a drainage that lead to Wild Cat Canyon Creek proper. After following the oil and mud downstream for about 400’ to the spill terminous, Murphy then contacted EBRPD steward Matt Graul and an OES Spill Report was issued. By the time Murphy reported the spill and followup activities to Silva, the flow had mostly dissipated but residual oil and wellbore fluids remained.

Next on site, EBRPD Biologist Steven Bobzien arrived and walked to the spill terminous where he was satisfied that spill did not reach Wildcat Canyon Creek. Graul then called for booms to be placed around the surface expression and in the creek and contacted a hazardous spill cleanup contractor, Photos 36-40.

Even with the extra activity generated by the spill, abandonment work progressed and an 82 cubic foot cement plug was placed at 1700 hours with tubing hanging at 500’.

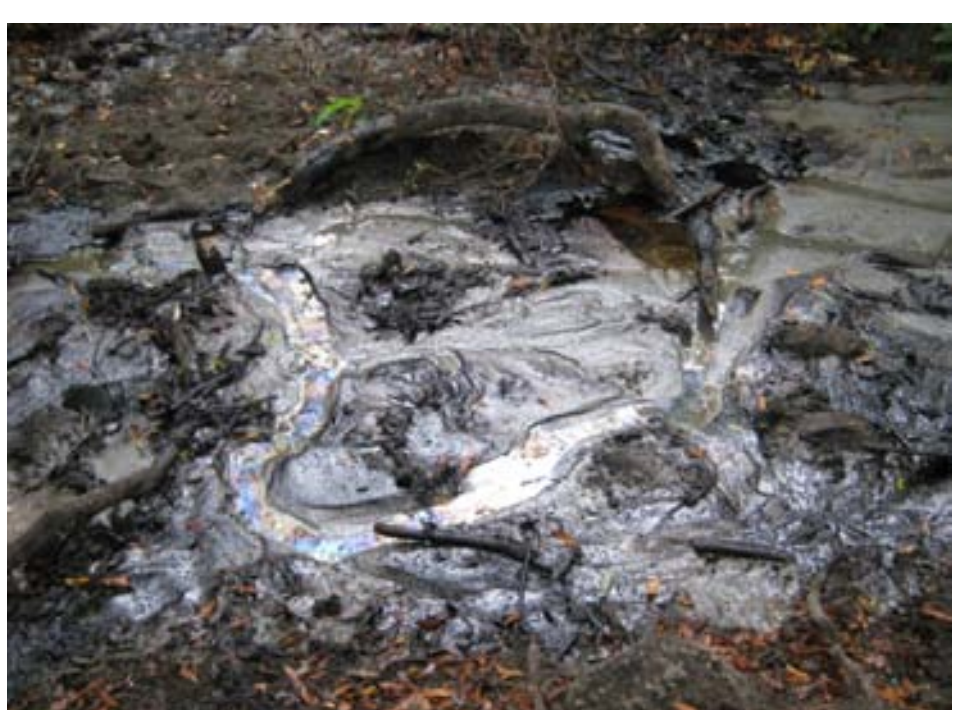

Photo 36. The surface expression resultant from well operations discovered by Murphy

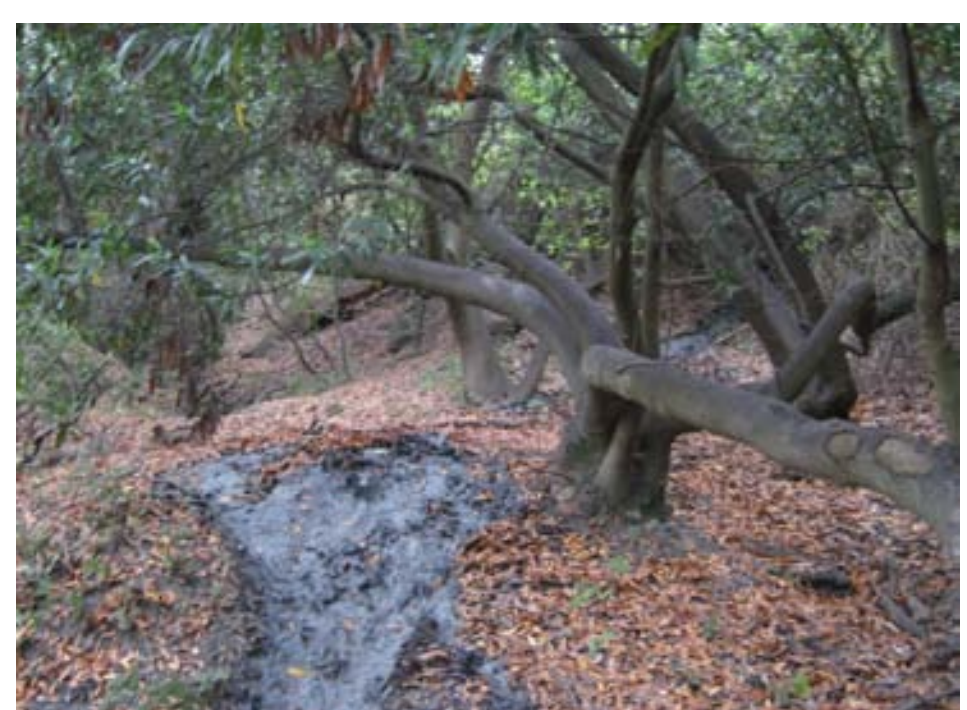

Photo 37. The flow path at the head of the spill

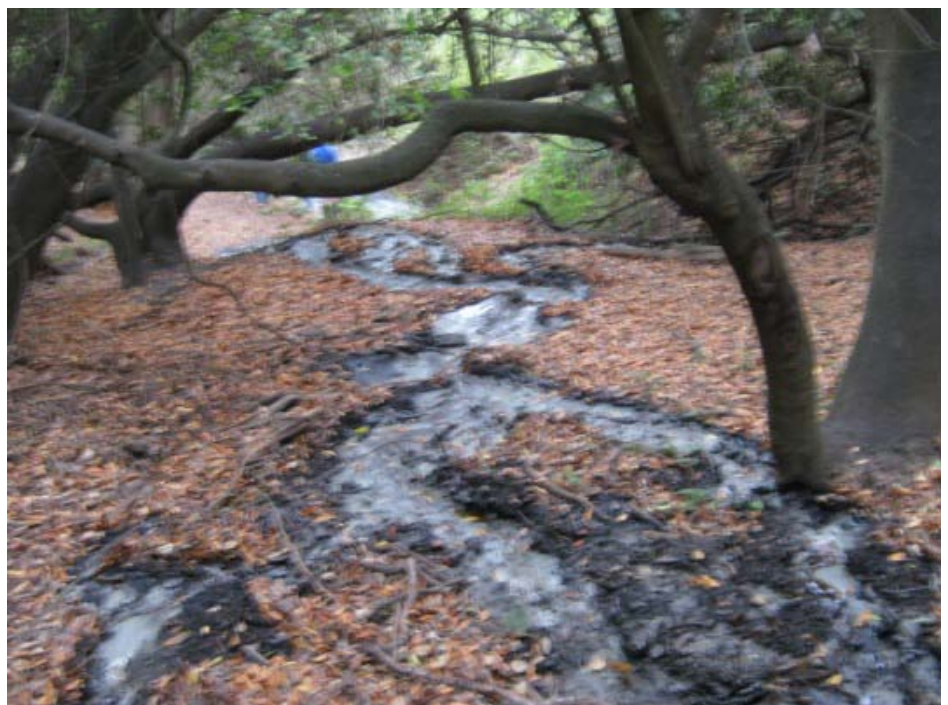

Photo 38. Flow path down hill

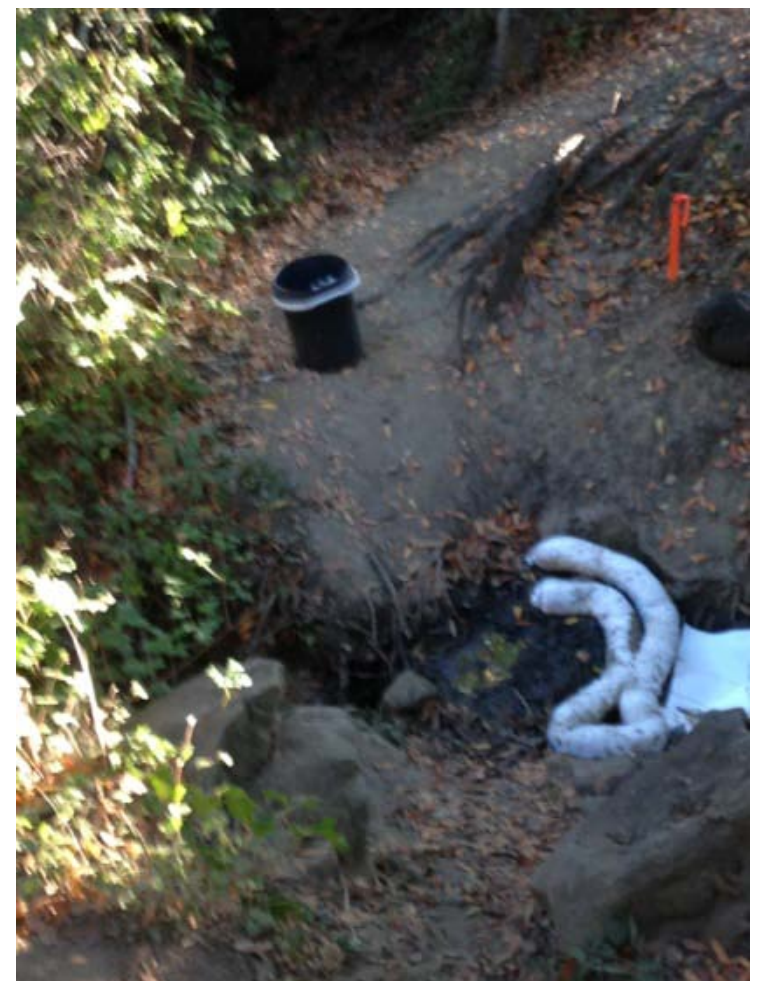

Photo 39. Absorbent booms in creek

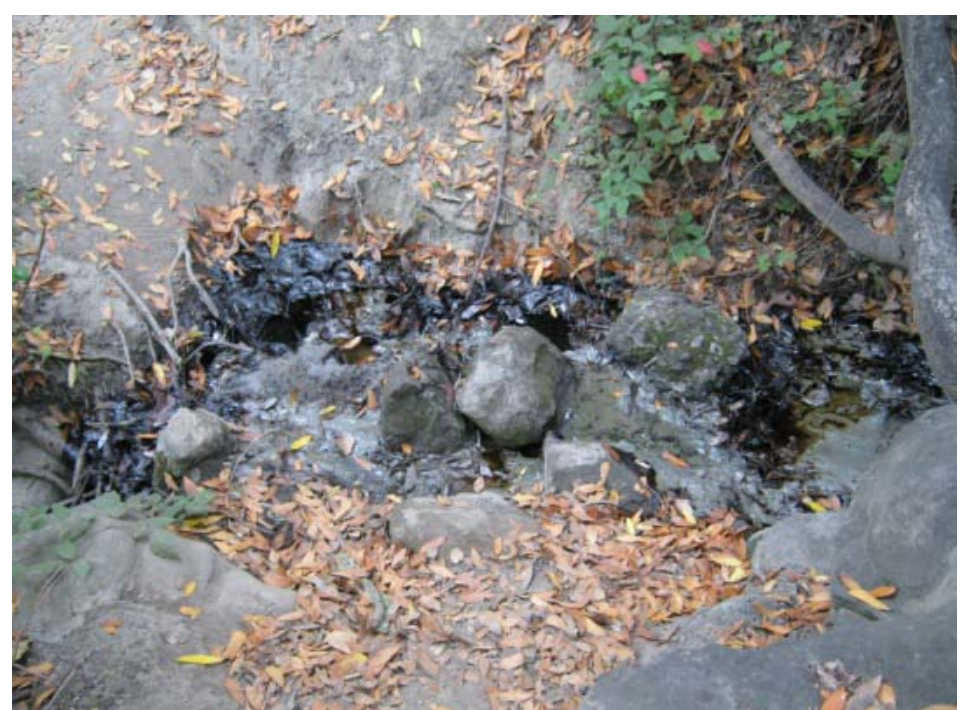

Photo 40. Post flow residual oil in creek

9/11/2013 – Wednesday

Abandonment operations continued along with clean up of oil water mixture in drainage, Photos 41-42. The bottom hole plug was tagged at 365'. An 8' interval from 360' – 352' was then perforated in order to squeeze cement into the annular space between supposed surface casing and open hole. At 1230 hours, Rival pumped a 92 cubic foot cement plug and the oil/water mixture continued to flow very slowly from its point of origin below the well pad into the ephemeral creek. At 1600 hours, Rival tagged with wireline at 346'. In this case 'no plug' was a good thing as a considerable amount of cement flowed out through perforations to the formation. Another cement plug of 92 cubic feet was then set at this depth.

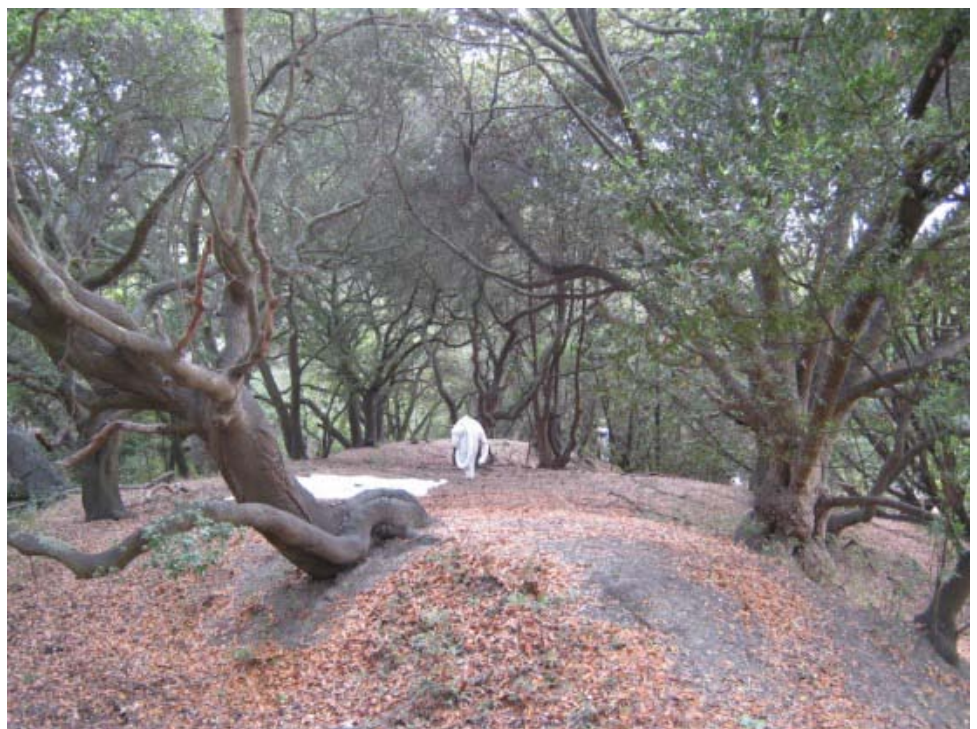 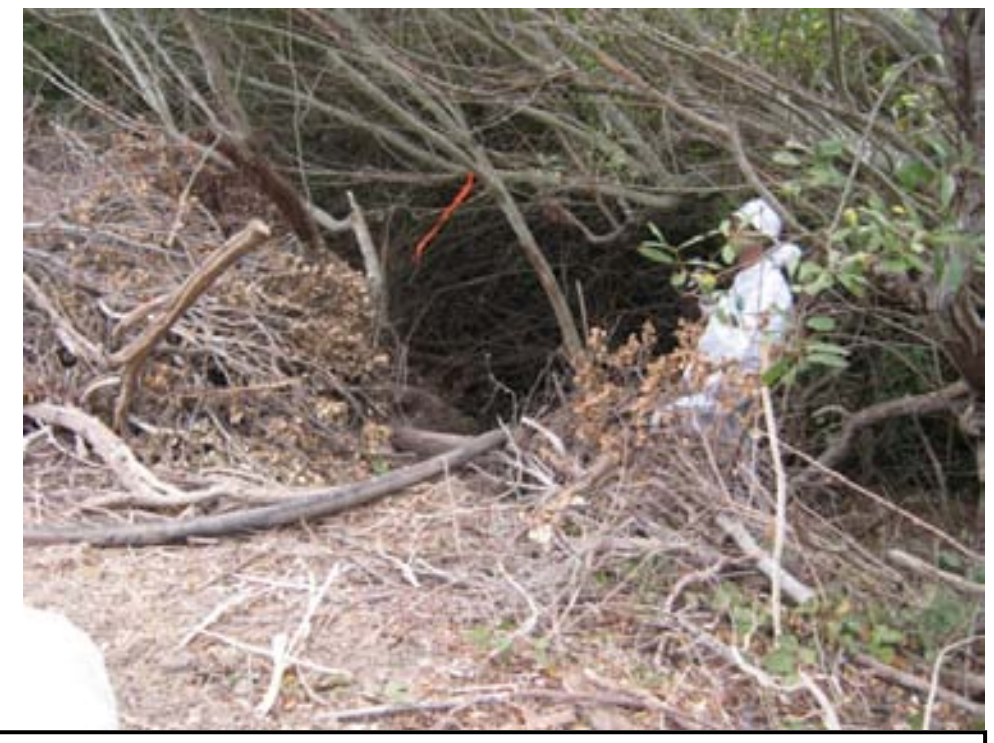

Photos 41-42. Cleanup work continues

9/12/2013 - Thursday

On Thursday morning DOGGR Associate Oil and Gas Engineer B.G. Tackett witnessed the bottom hole plug with 2 7/8" tubing at 147' but with the top ten feet being green, the hard tag was logged at 157'. Rival came out of the hole laying down and removed the BOPE while waiting for the next load of cement. At 1145 hours an additional 92 cubic feet was placed and the surface expression began flowing once again. There were returns to surface but no cement. Pumping an additional 20 sacks, there were still no cement returns. At 1250 hours Rival pulled out of hole and laid down tubing.

9/13/2013 – Friday

Upon arriving at the well site, DOGGR staff inspected the surface expression to find there was no longer any flow. The last cement job had finally plugged the annular space and stopped sub-surface communication. Spill cleanup was progressing and the plug was tagged at 31'. At this time Rival rigged down and moved off the well and any remaining fluid was vacuumed from the well bore and shoring box with the on site vacuum truck, Photo 43-44.

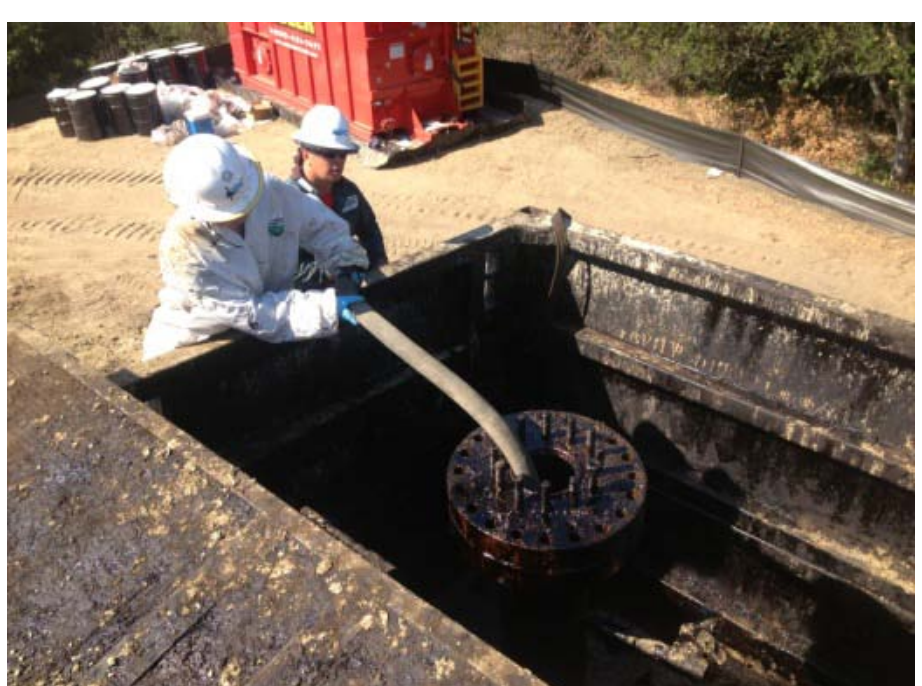 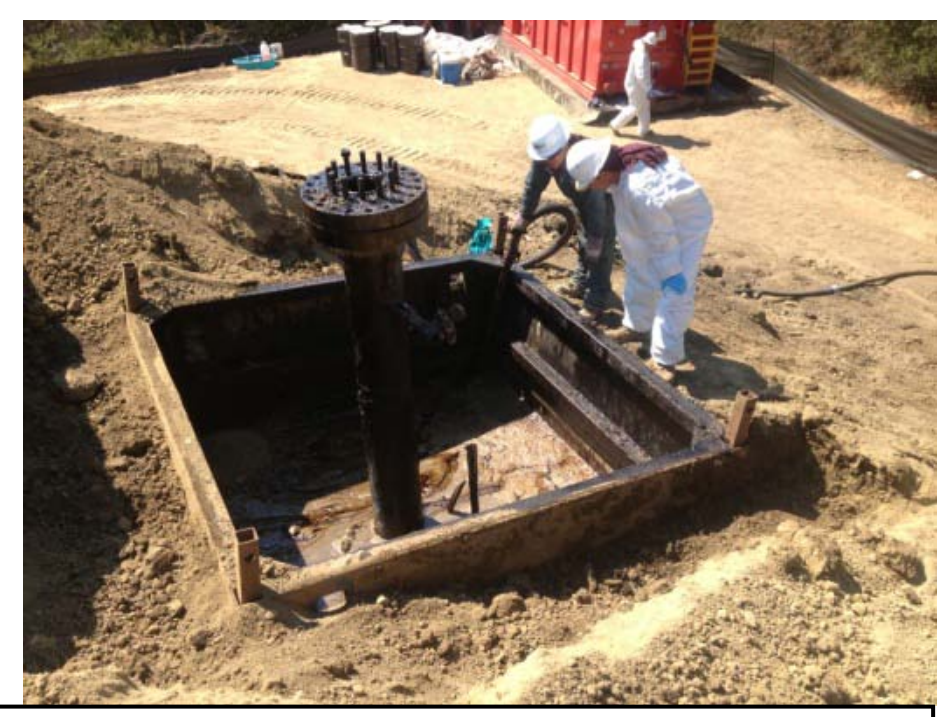

Photo 43-44. Vacuuming wastes from the well bore and shoring

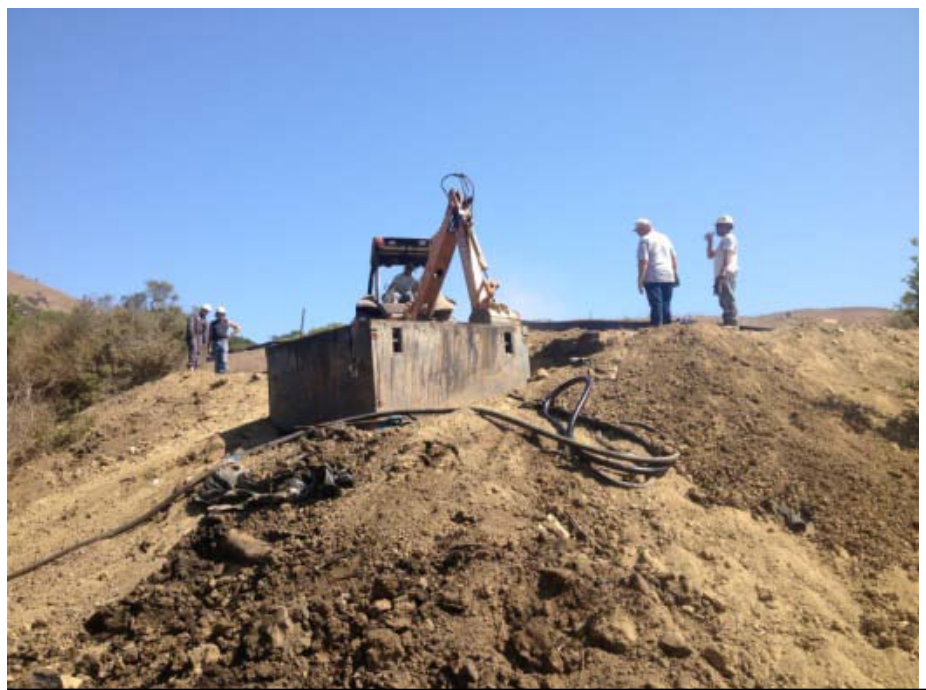
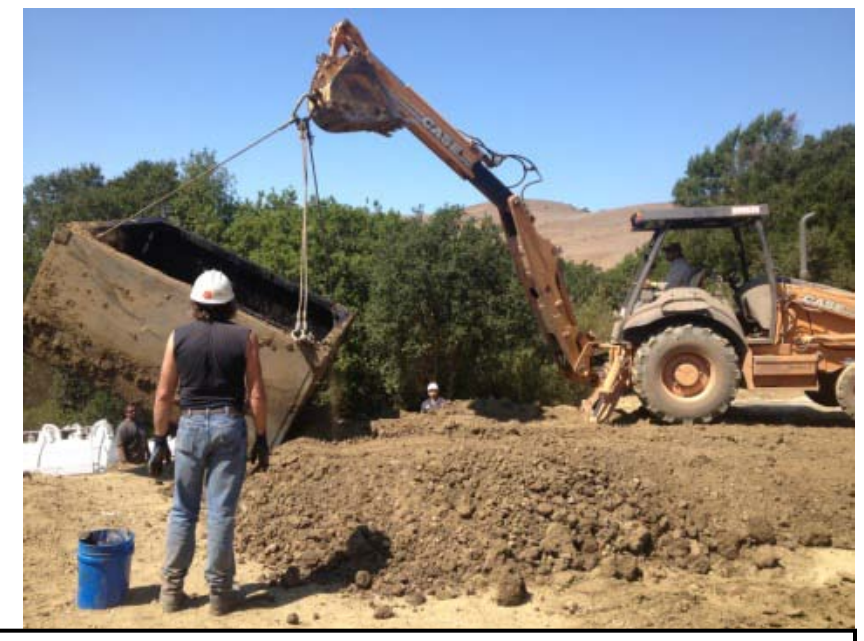

Photo 45-46. Shoring box being excavated and removed

Once the shoring box was removed contaminated soils were removed from the well leaving an approximately 12' casing stub. At this time a safety chain was installed from the backhoe shovel and the wellhead was cut off using an oxy-acetylene cutting torch. Prior to spotting the final surface plug, fluid was once again vacuumed from the well bore, and the metal plate was welded on. Ultimately, the leaking of the well over time contaminated approximately 40 cubic yards of soil, and at job's end, a total of 50 yards of material had to be removed. Too, 12 barrels of waste from the spill was generated in addition to a mixture of oil and water stored in a now full Baker Tank, Photos 45-52.

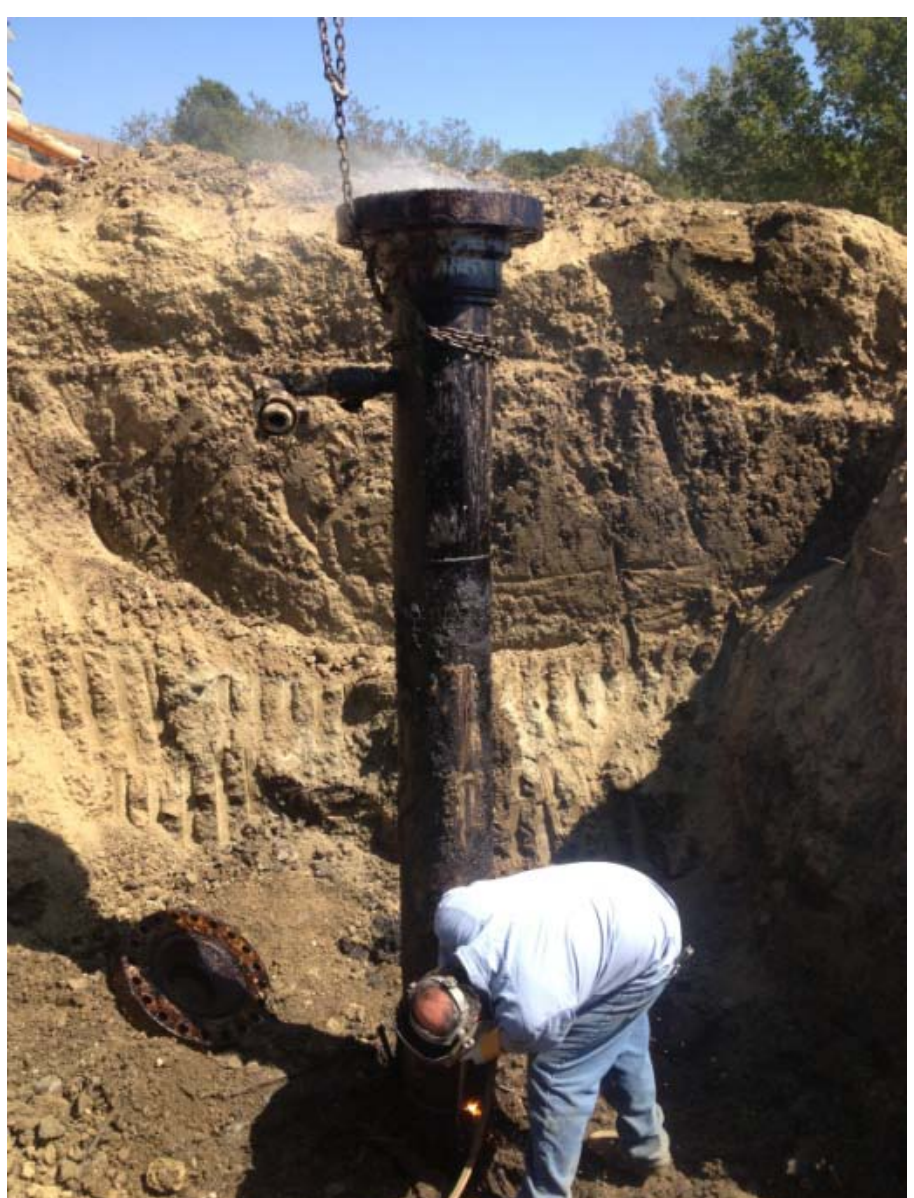
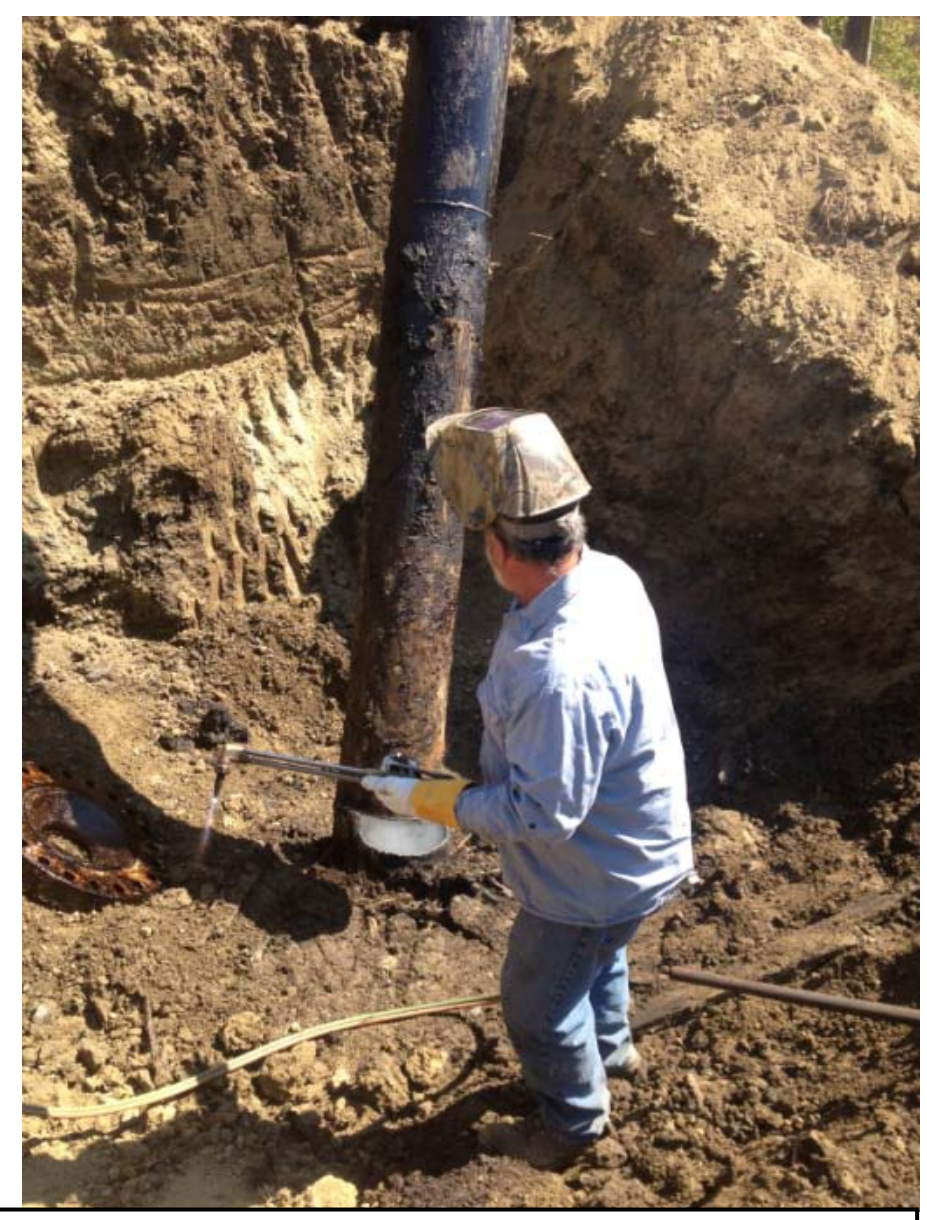

Photos 47-48. Cutting off the Wellhead

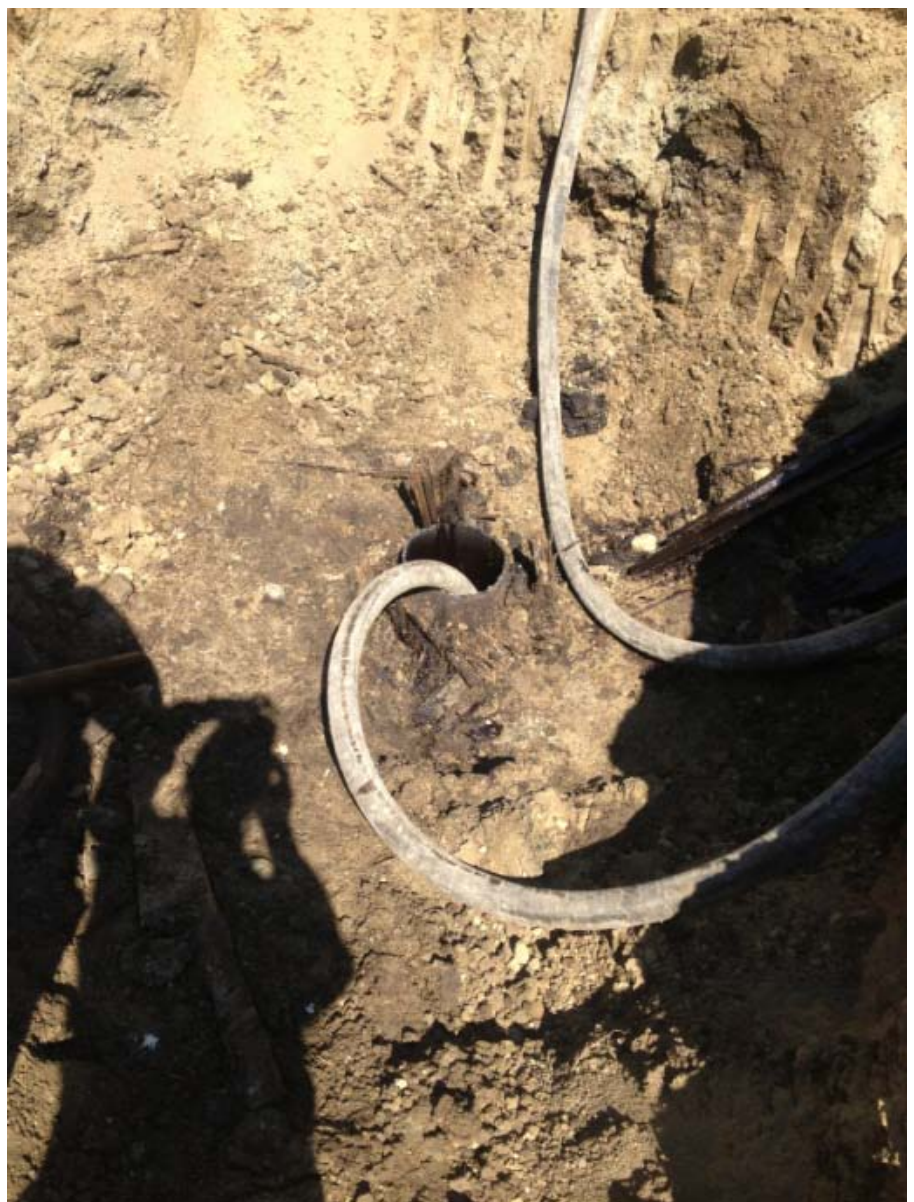

Photo 49. Final fluid evacuation from the wellbore

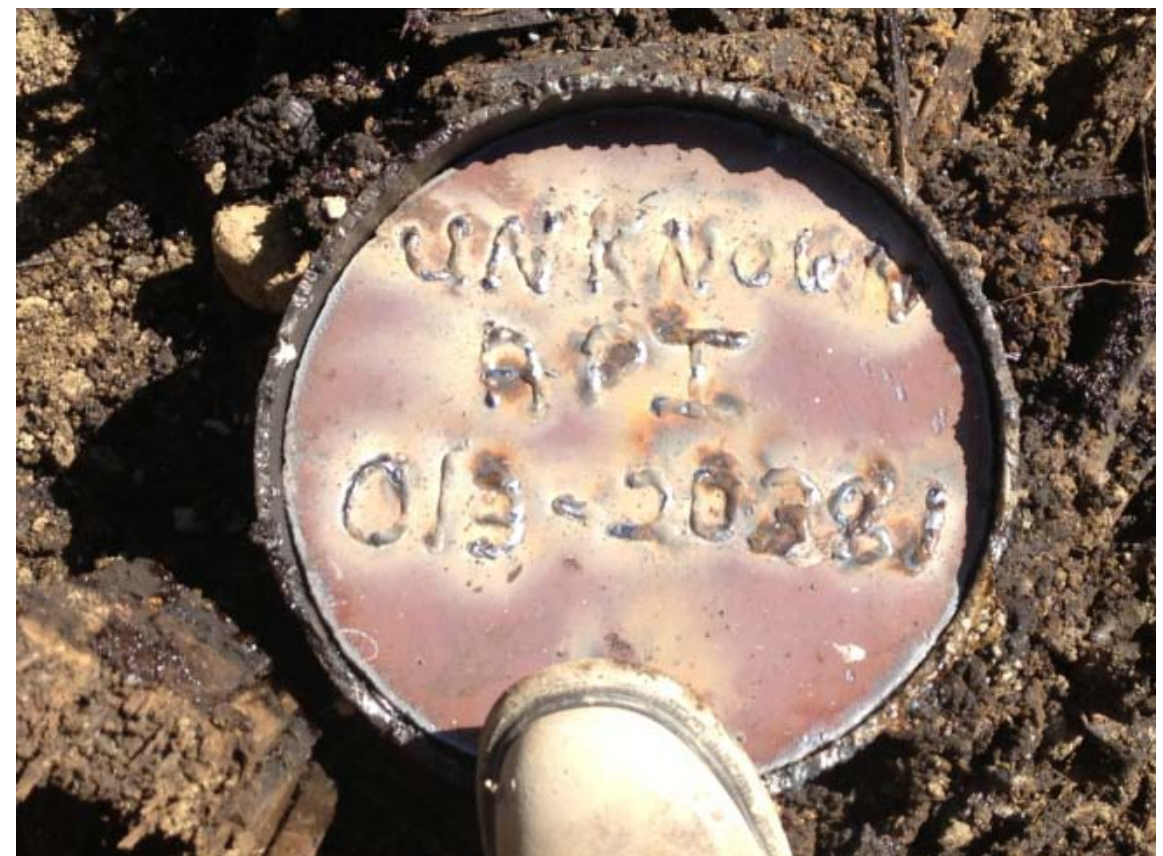

Photo 50. "Unknown" 1, API 013-20381 plugged and abandoned

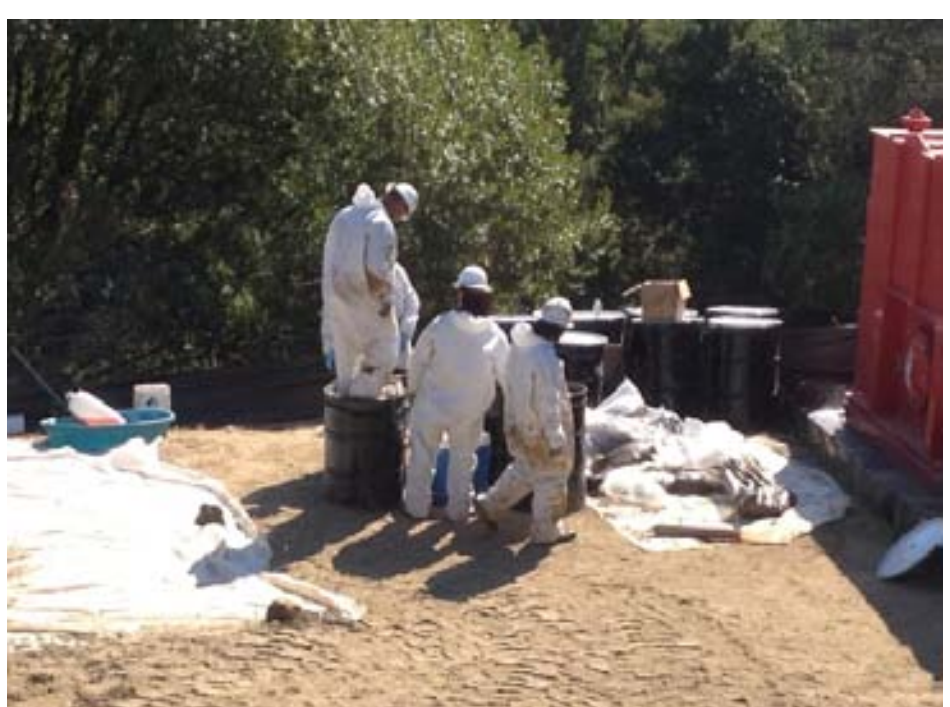

Photo 51. Cleanup crew packing barrels of oil residue

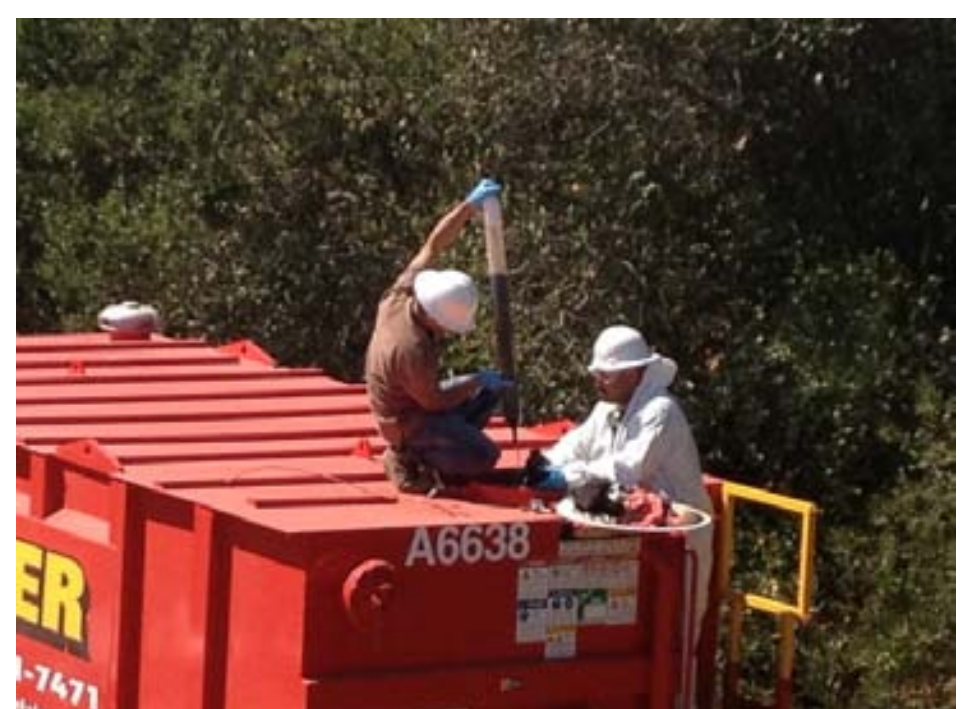

Photo 52. Cleanup crew sampling waste tank for oil

11/18/2013 – Monday

A post work inspection was performed by DOGGR staff and consulting wildlife biologist Amanda Murphy. The cleanup of the well site, access roads, and most importantly the ephemeral creek, was approved. Erosion control and hydro mulching was installed on the graded access roads, the well site was recontoured, and the quiet was once again restored to Wildcat Canyon Regional Preserve, Photos 53-59.

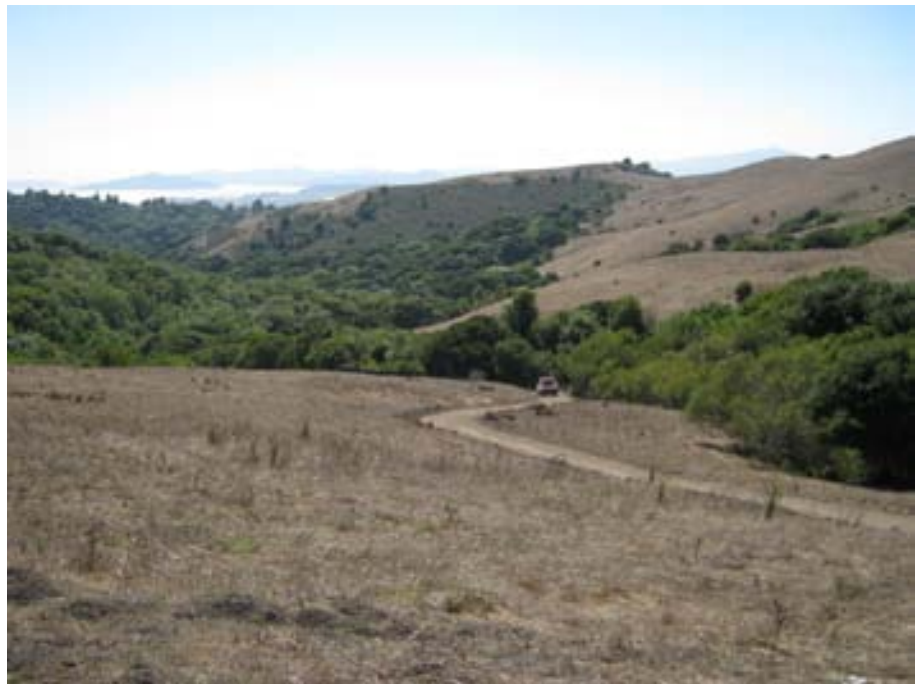

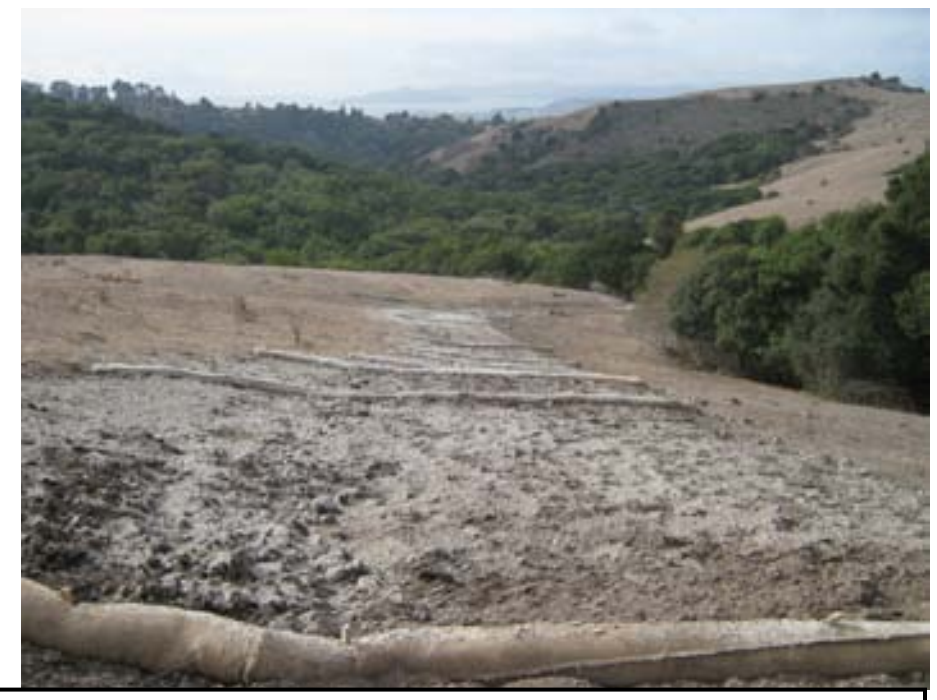

Photos 53-54, Access road, graded and restored.

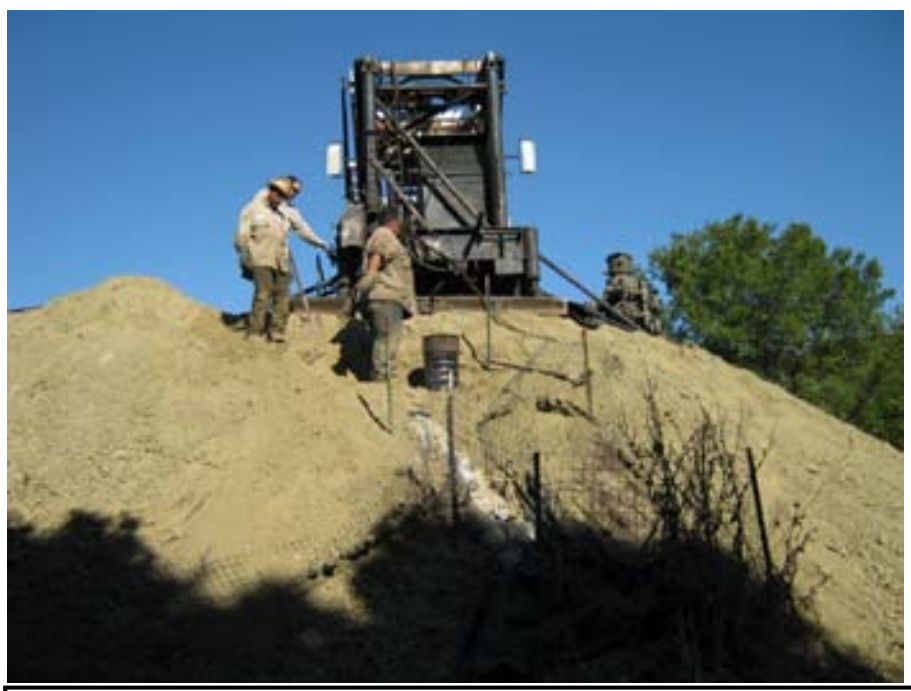

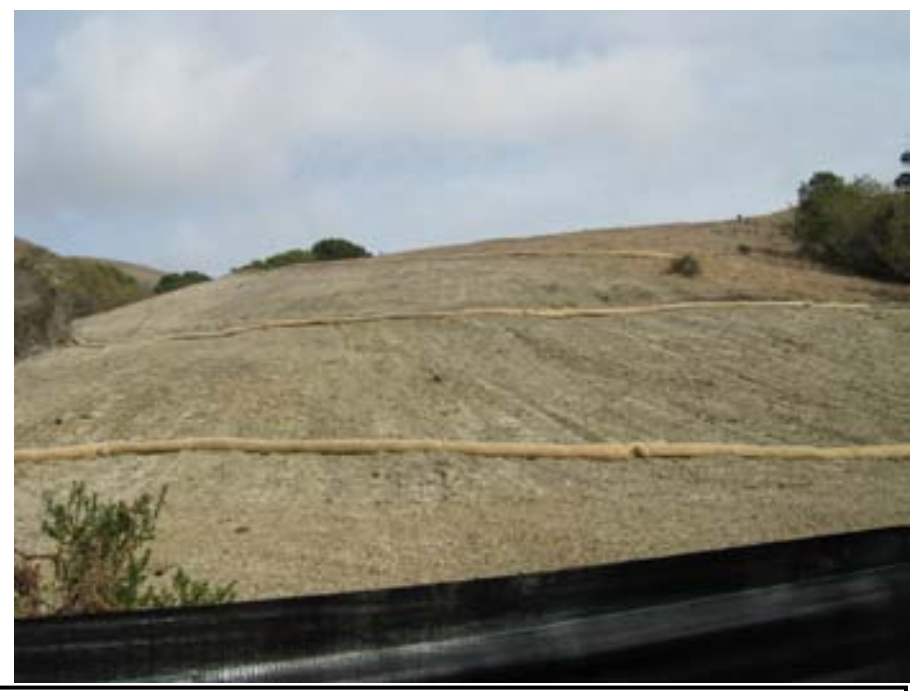

Photos 55-56. Wellsite before and after

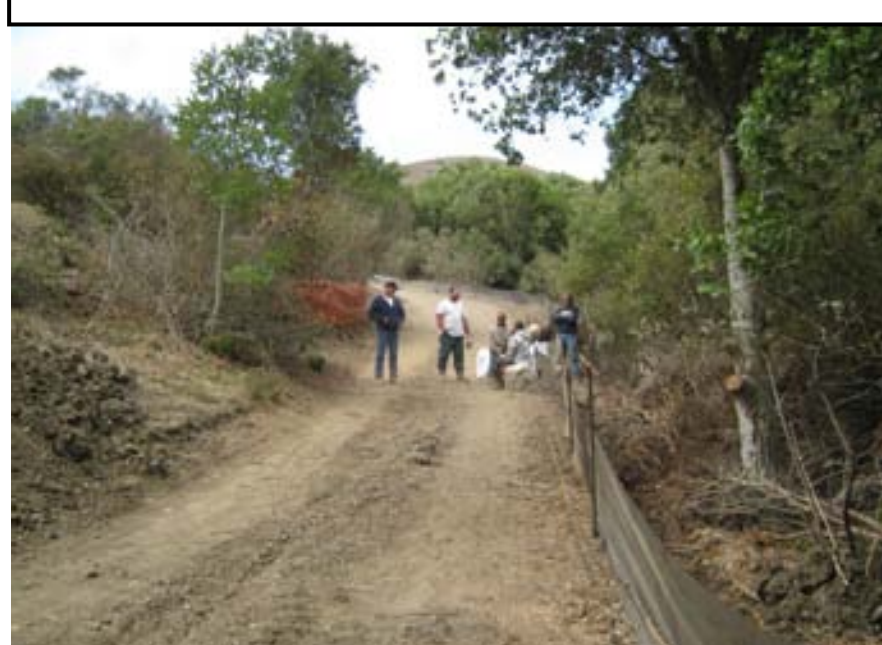

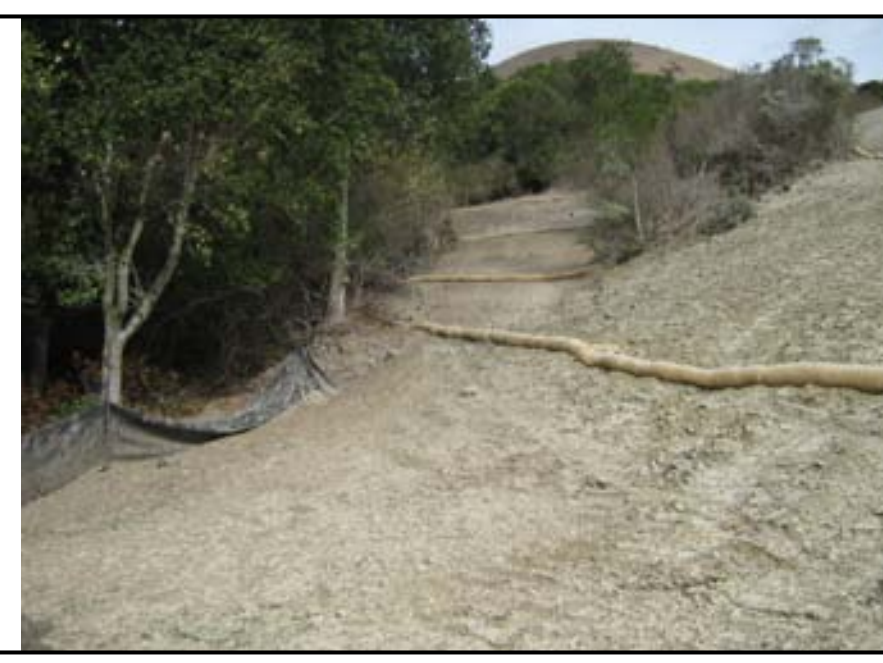

Photos 57-58. Access road to the lower pad

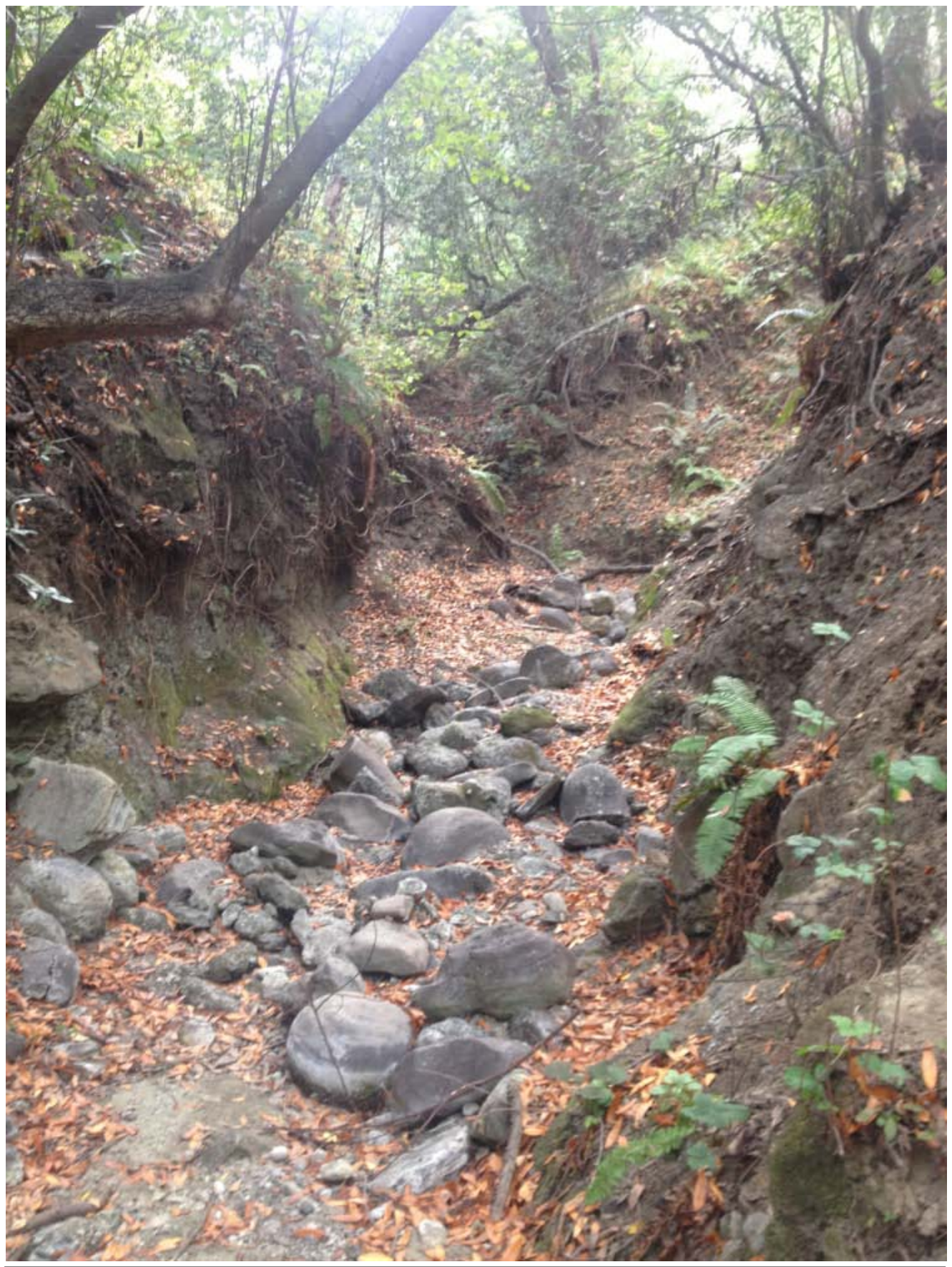

Photo 59. A cleaned ephemeral tributary to Wildcat Canyon Creek waiting winter rains

EXPENDED FIELD TIME

**Date**	**Start Time**	**End Time**	**Total Hours**	**OT Hours**
8/22/2013	0900	2030	11.5	3.0
8/23/2013	0430	2130	17	8.5
8/24/2013	0500	1430	9.5	1.0
8/26/201	0730	2000	12.5	4.0
8/27/2013	0500	2030	15.5	7.0
8/28/2013	0900	1930	10.5	2.0
8/29/2013	0800	1400	6	0.0
8/30/2013	0830	1200	3.5	0.0
9/3/2013	0900	2030	11.5	3.0
9/4/2013	0530	1975	15	6.5
9/5/2013	0445	1745	13	4.5
9/6/2013	0445	1415	9.5	1.0
9/9/2013	0745	1945	12	3.5
9/10/2013	0430	2000	15.5	7.0
9/11/2013	0430	2000	15.5	7.0
9/12/2013	0430	2030	16	7
9/13/2013	0430	2130	17	7
9/18/2013	0700	1500	8	0.0
**TOTAL**			219	58

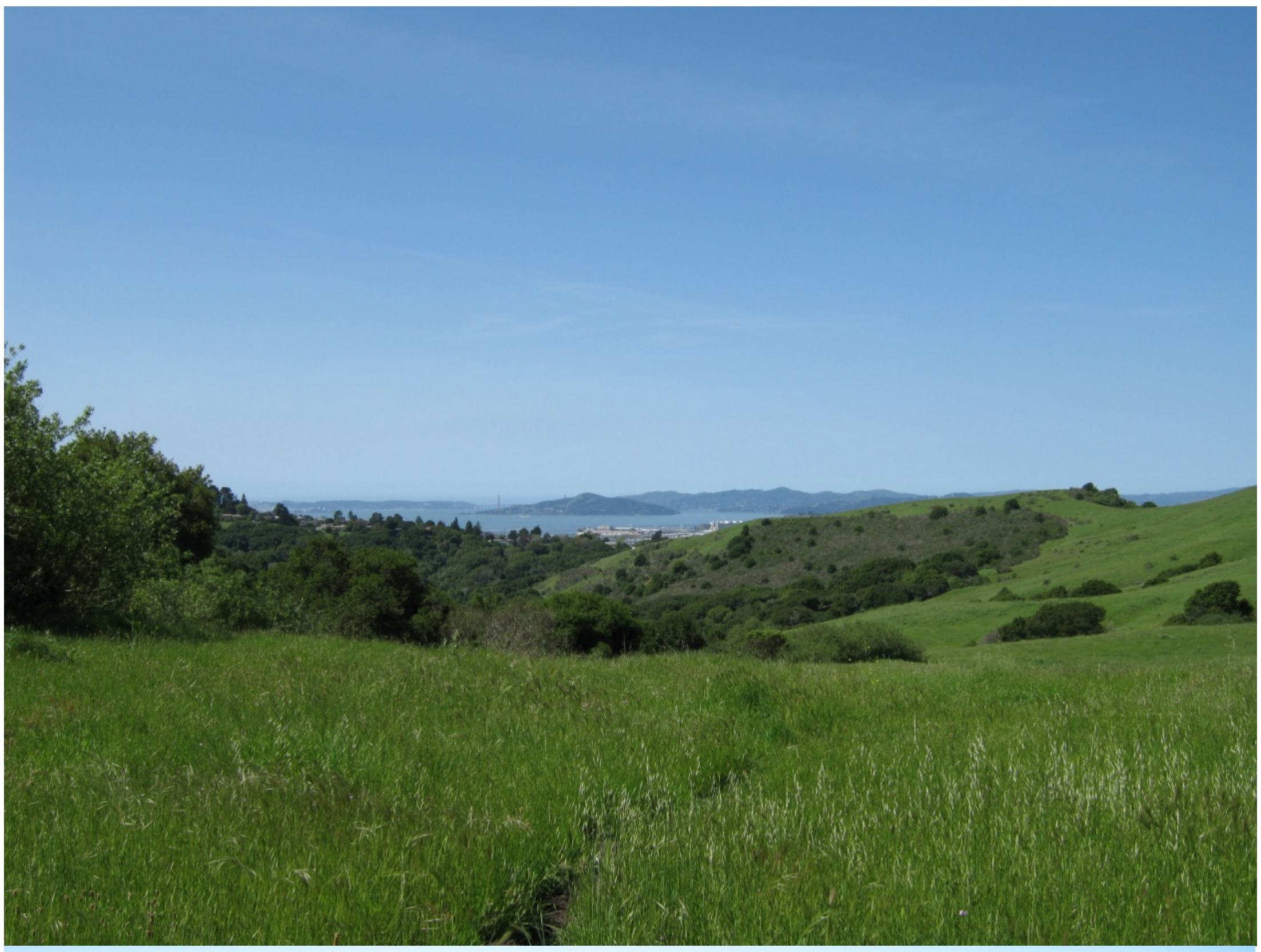

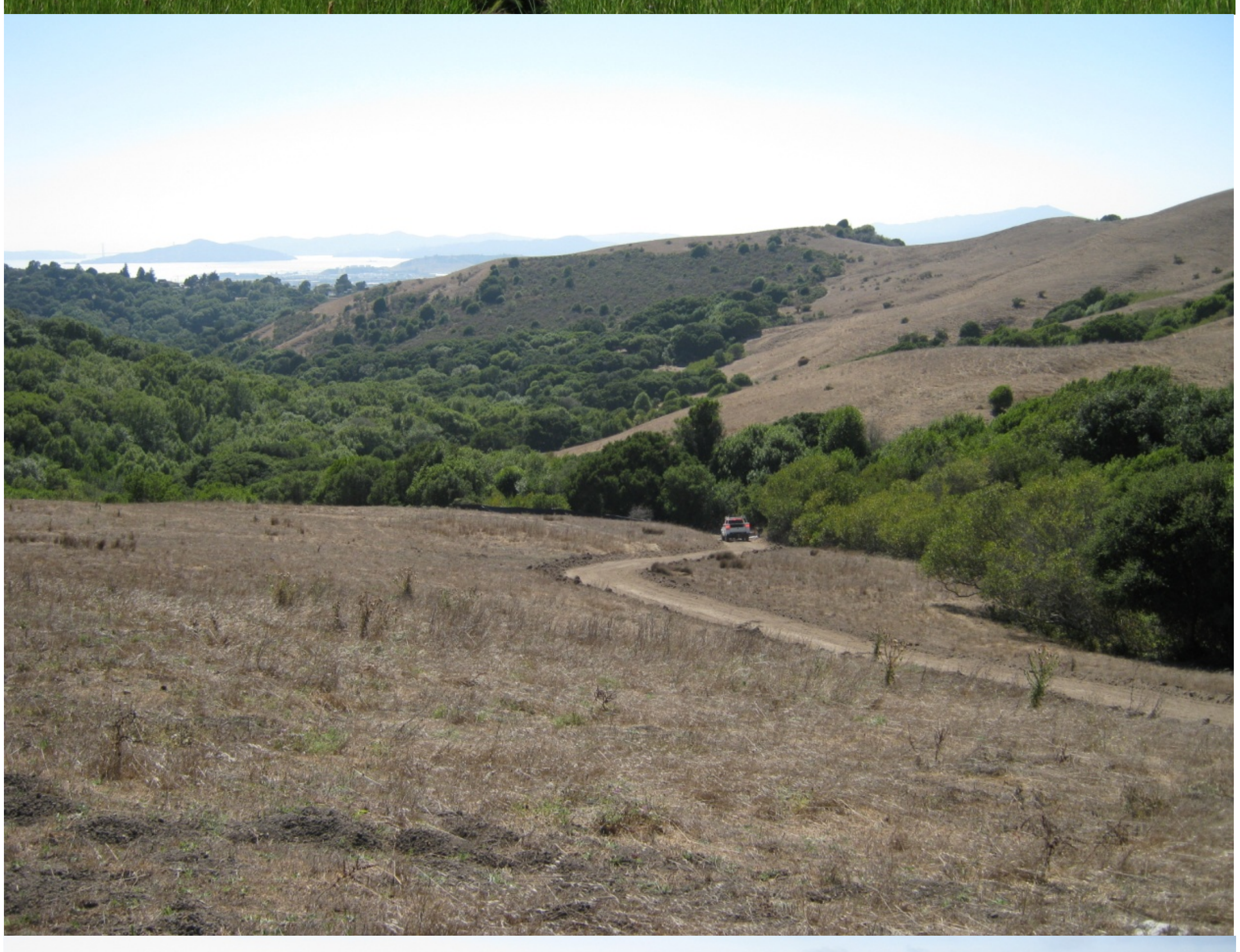

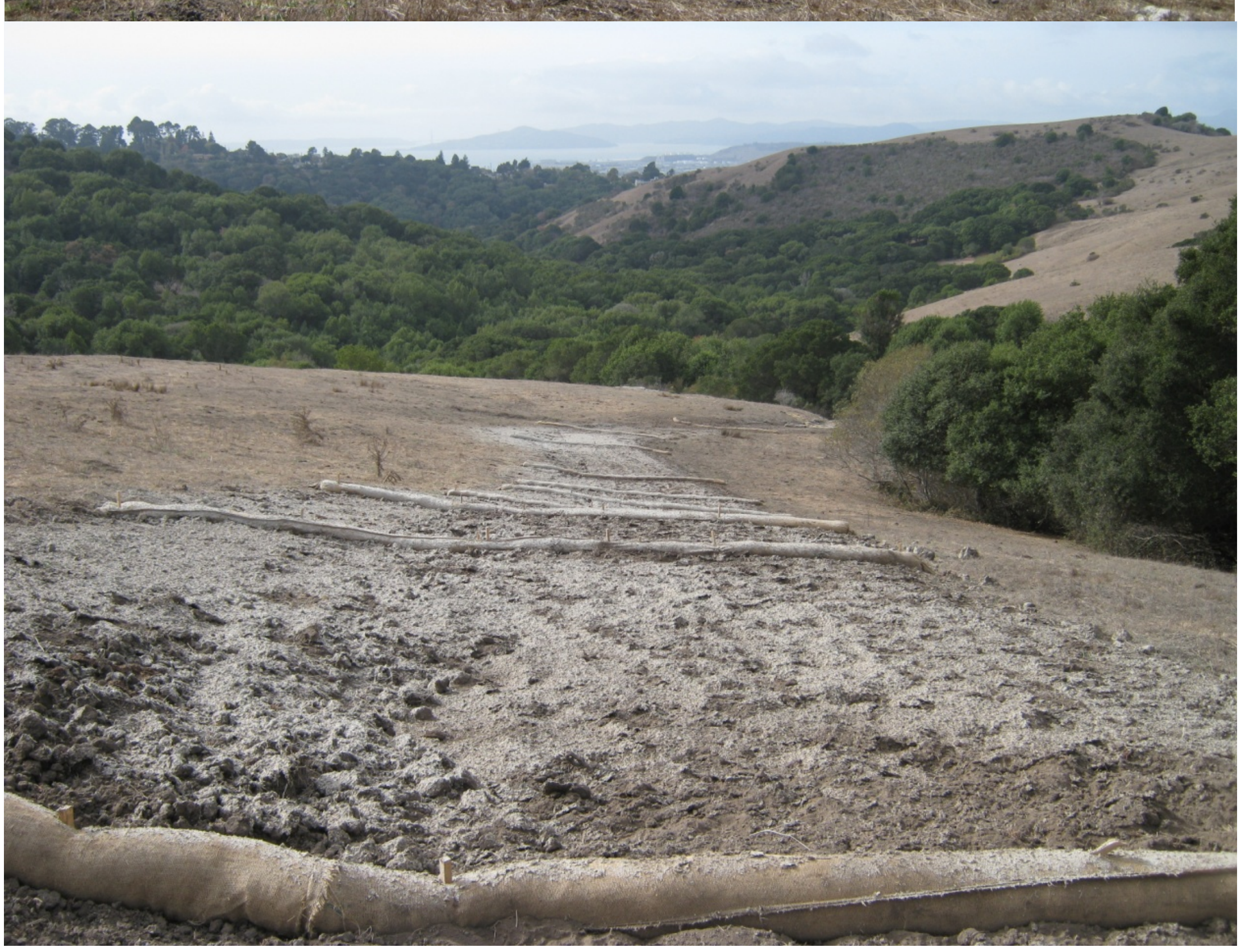

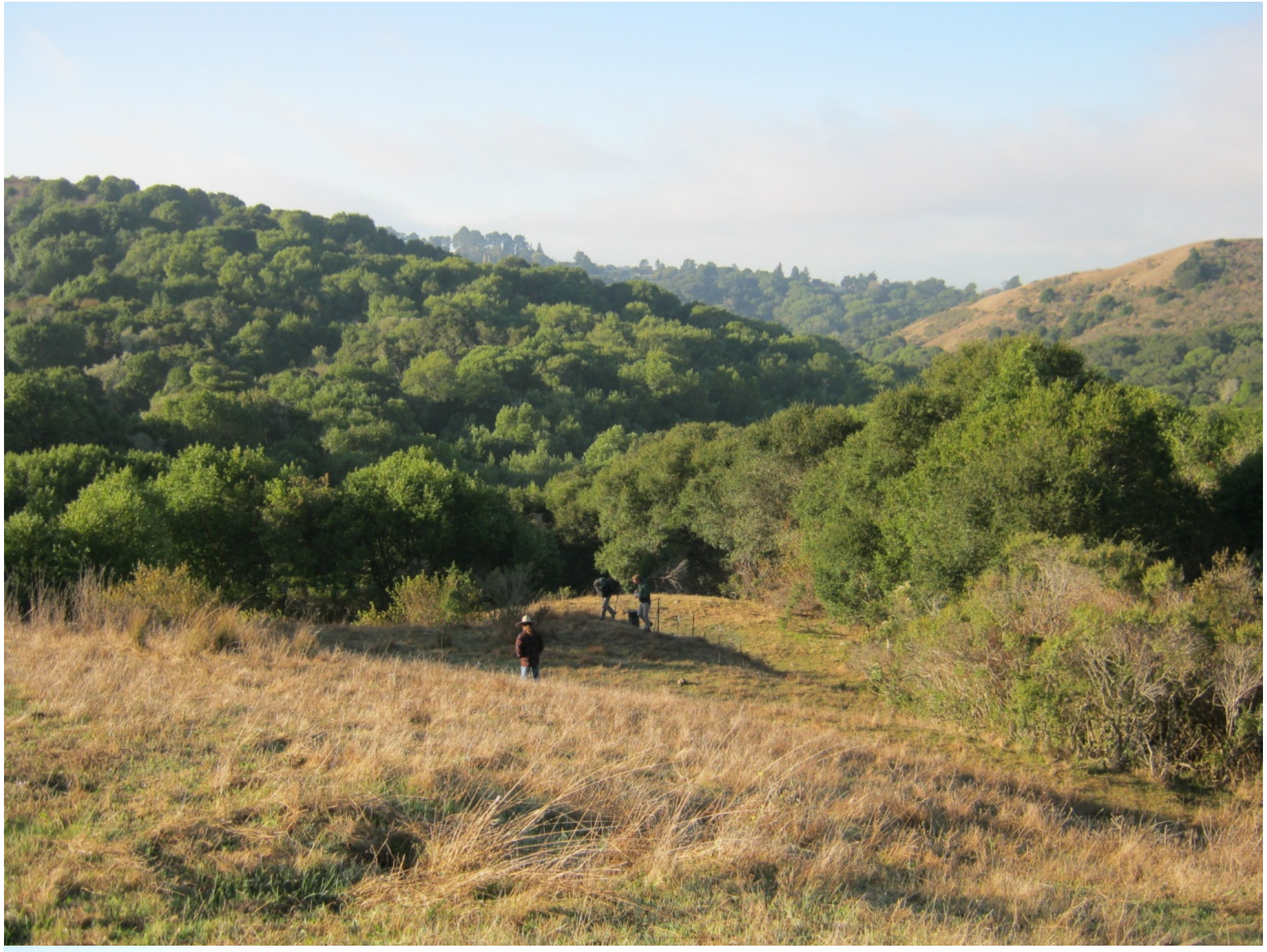

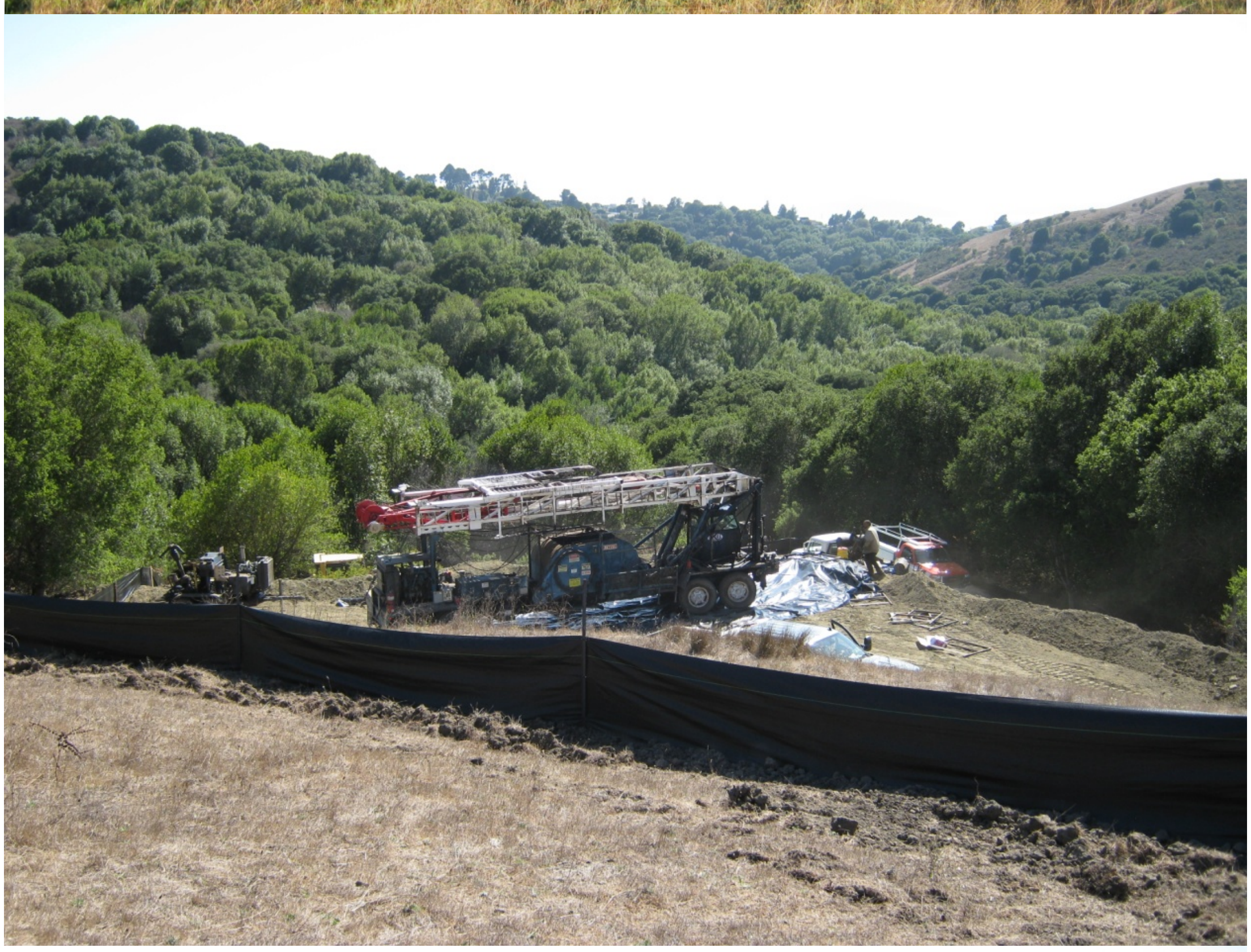

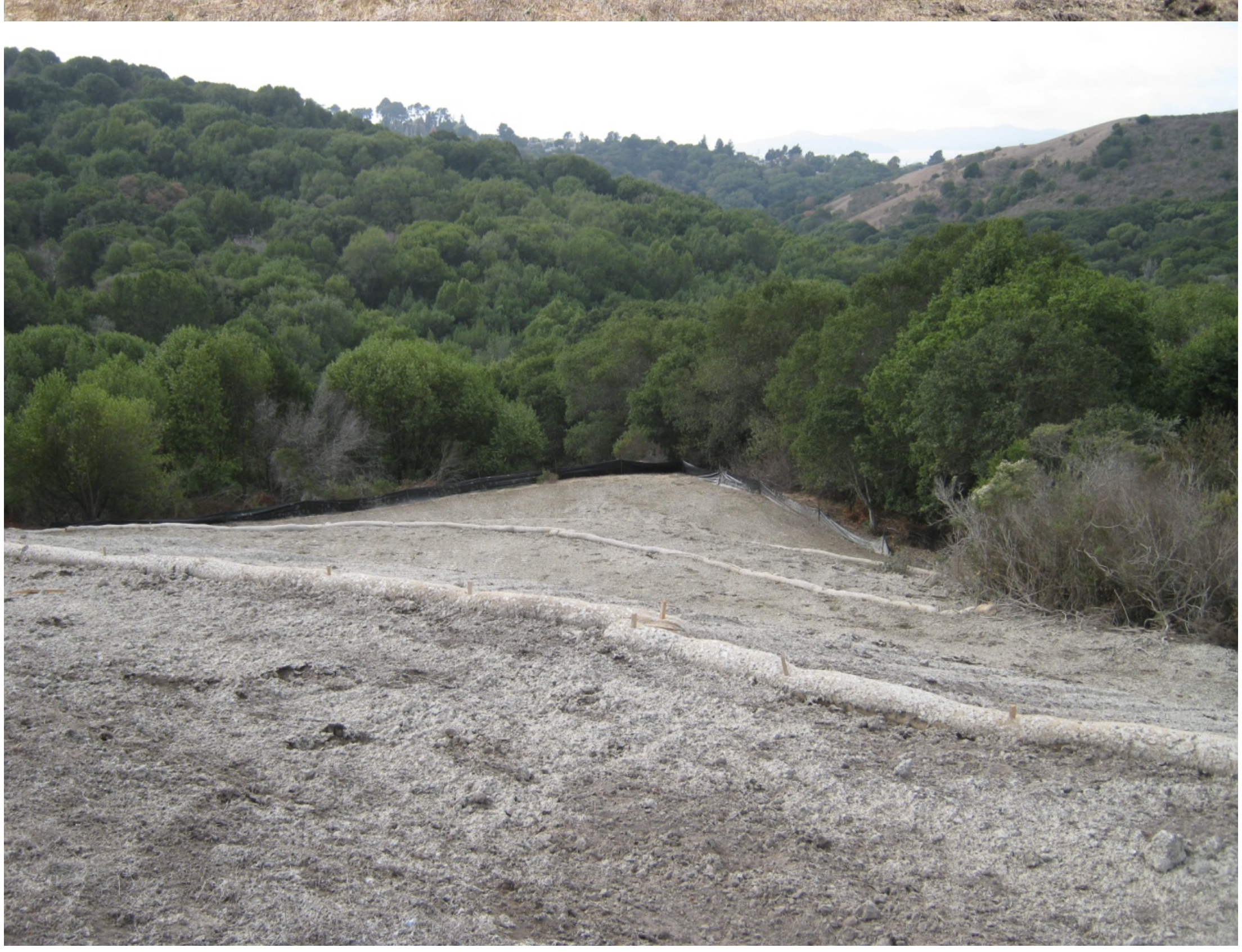

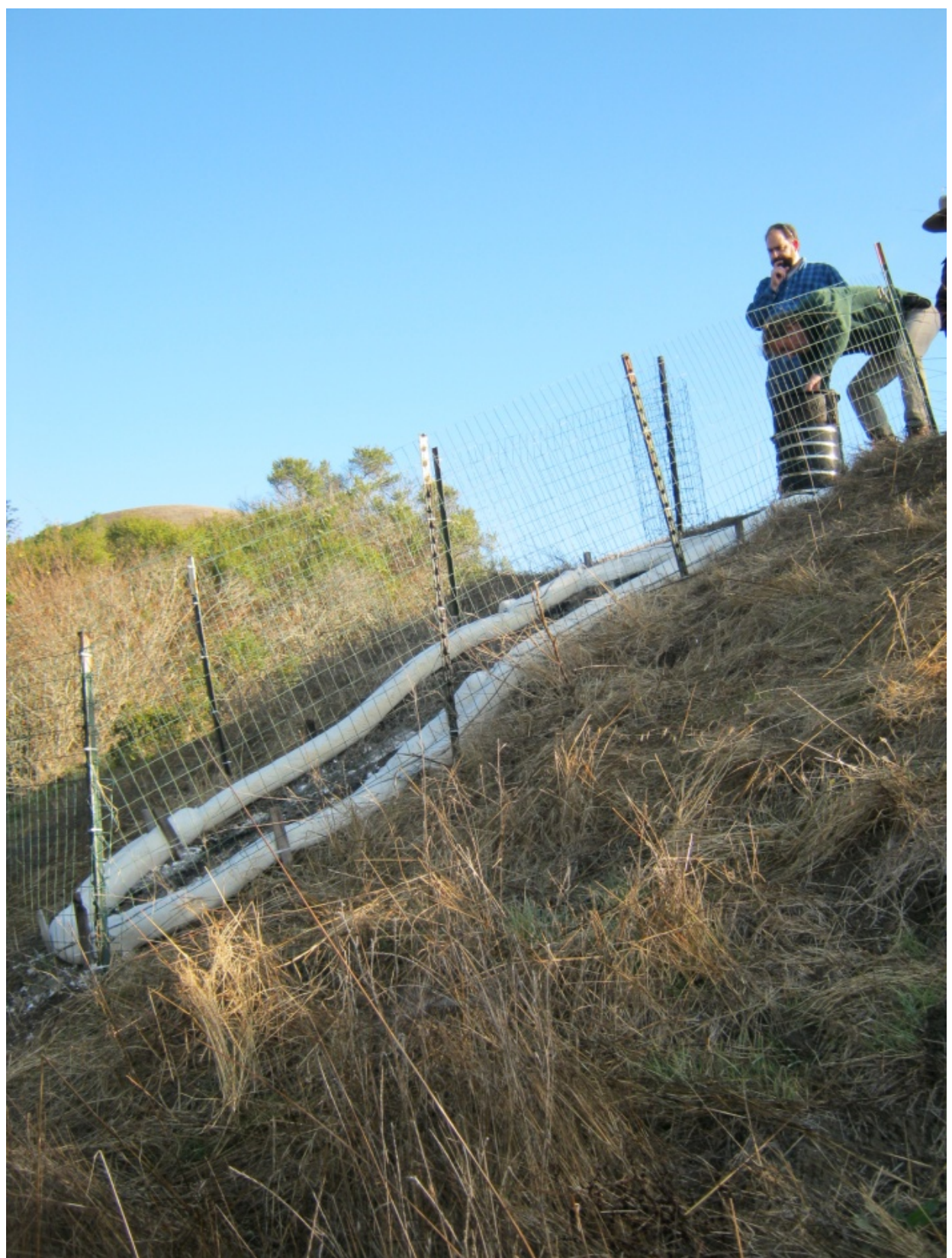

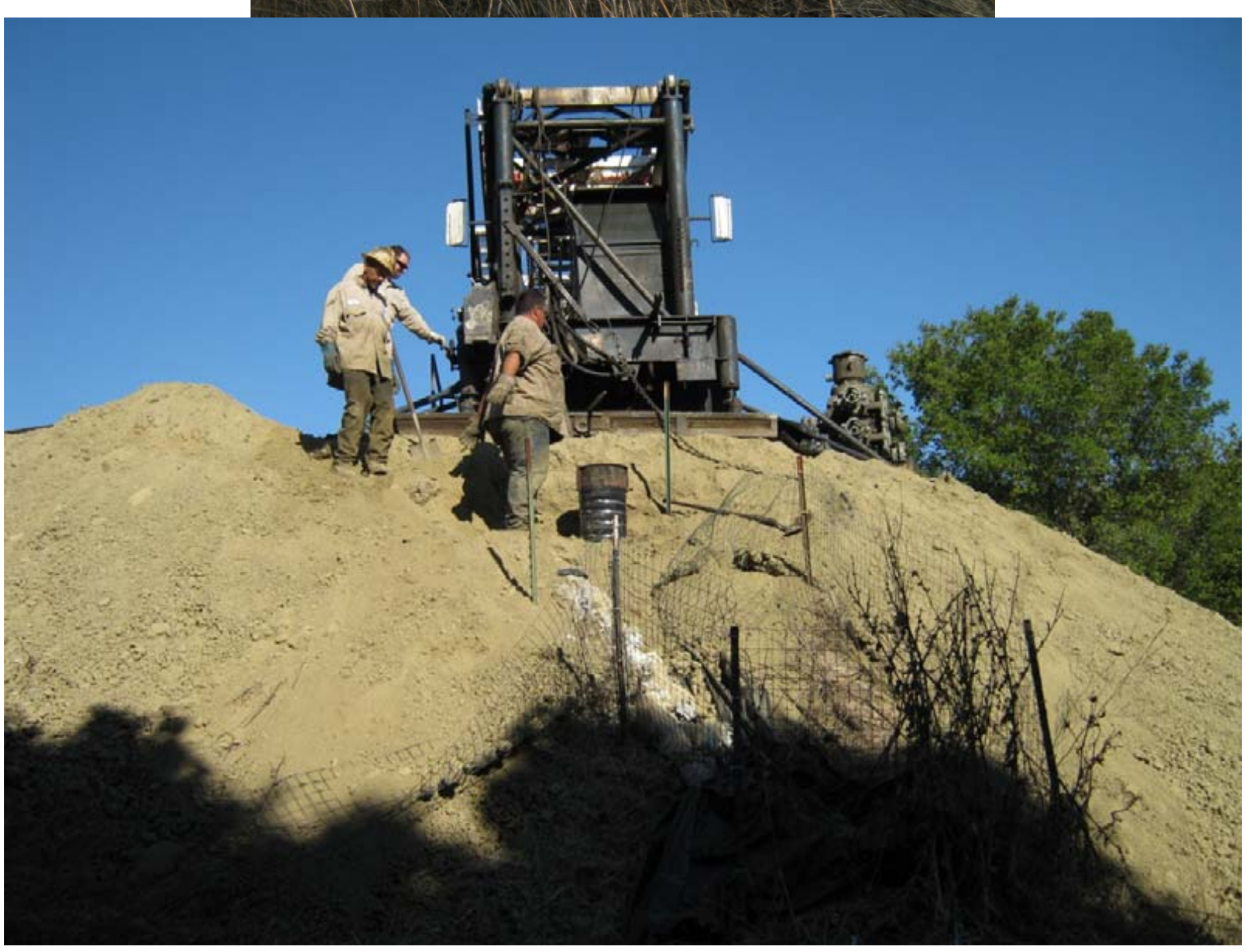

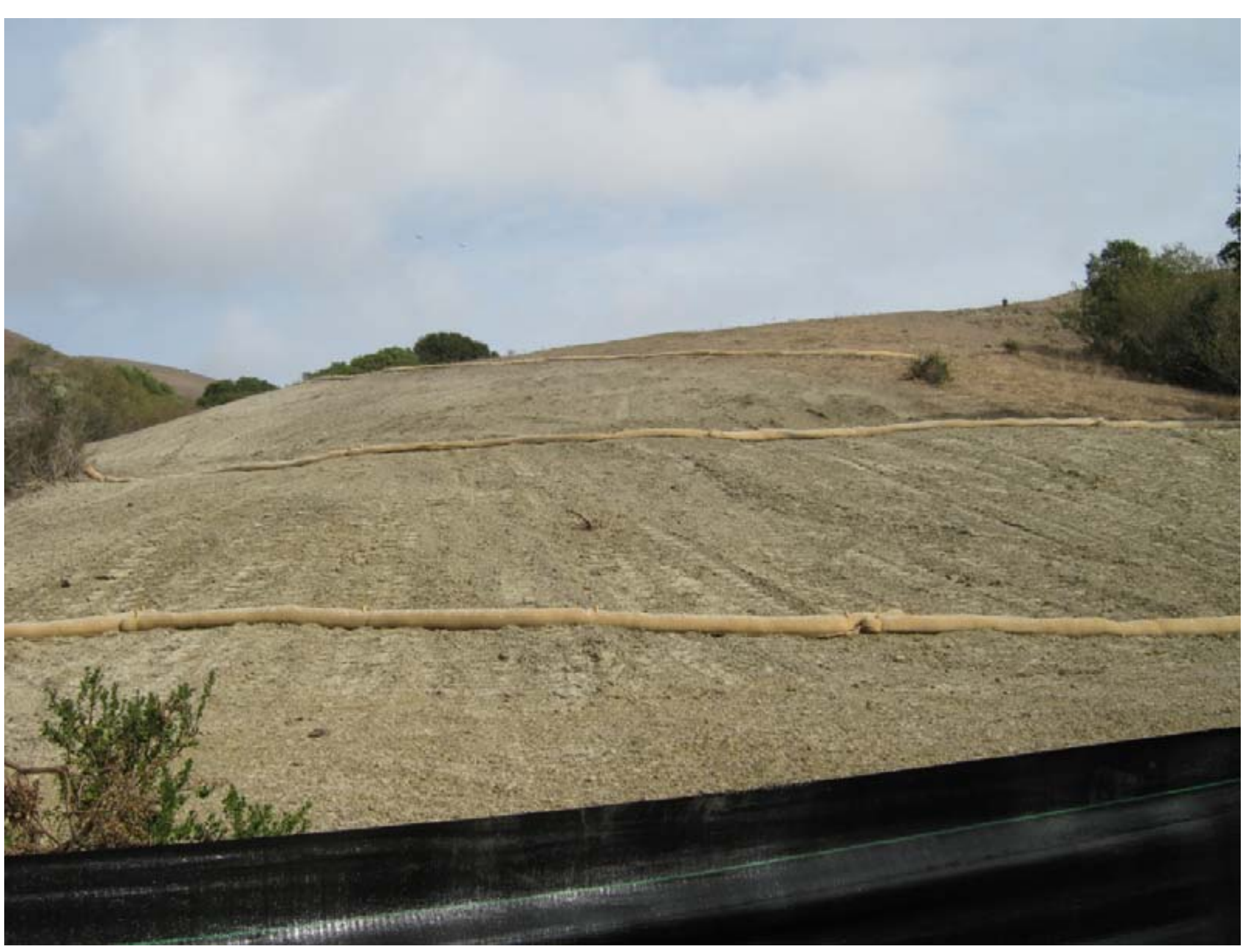

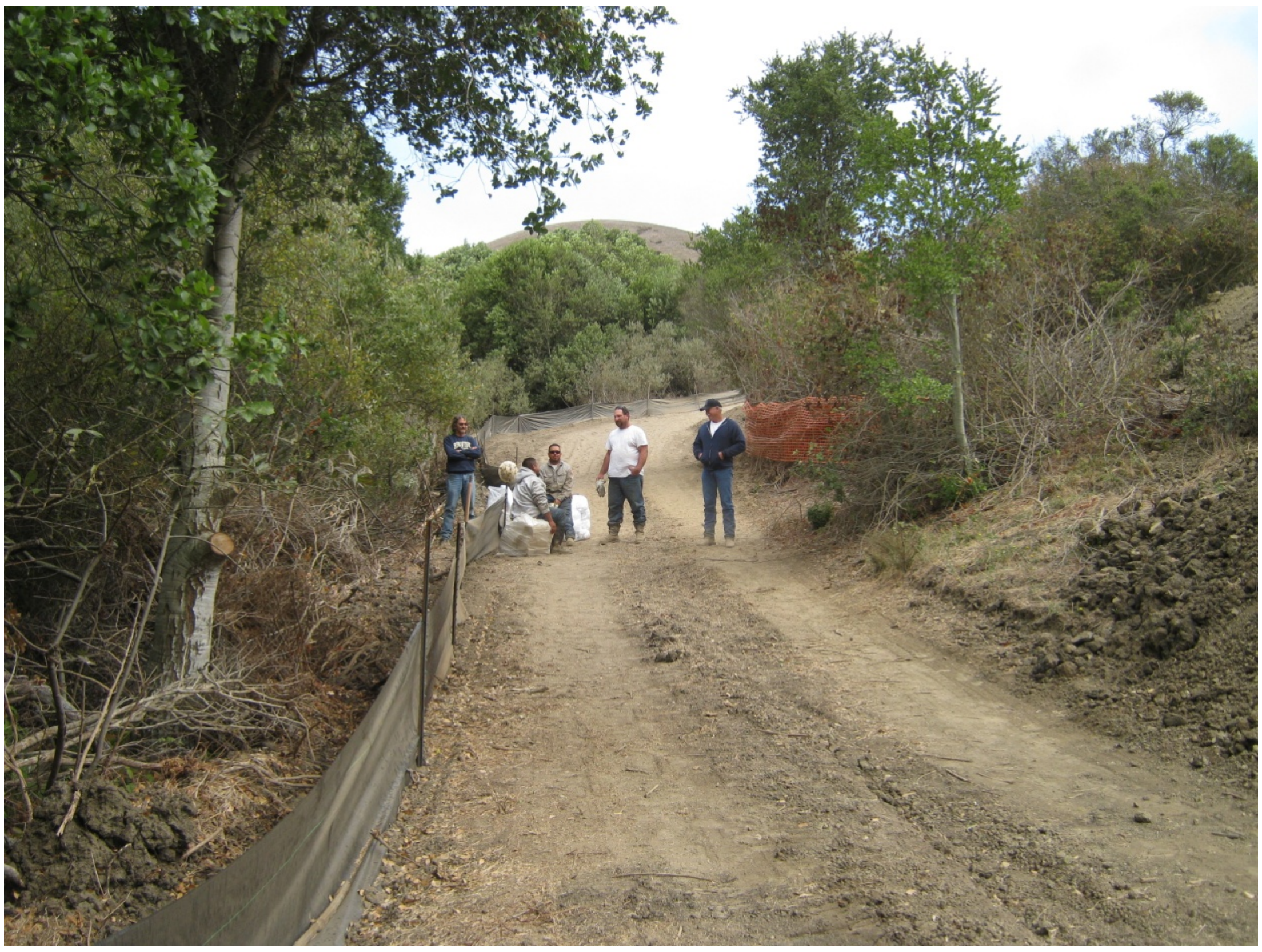

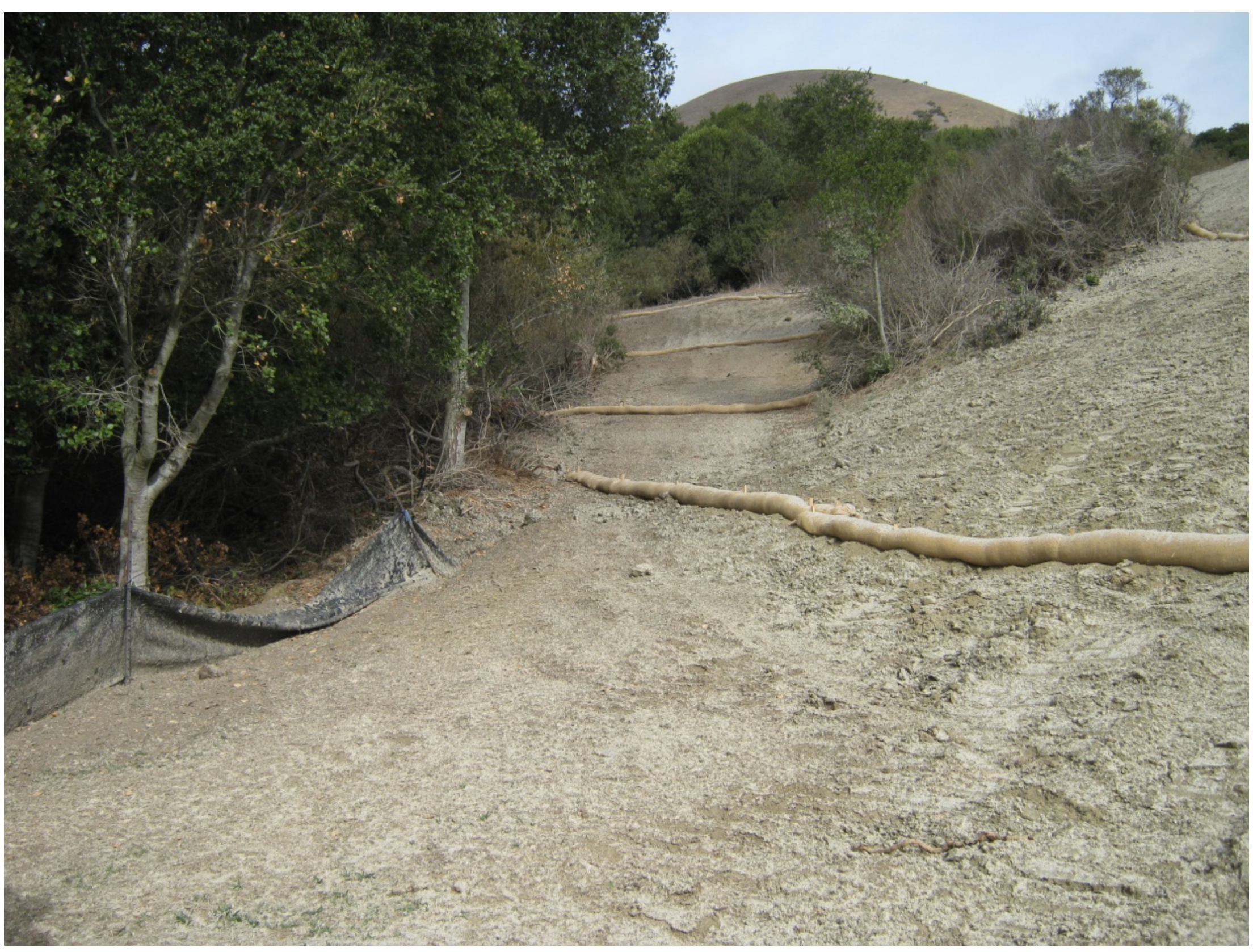

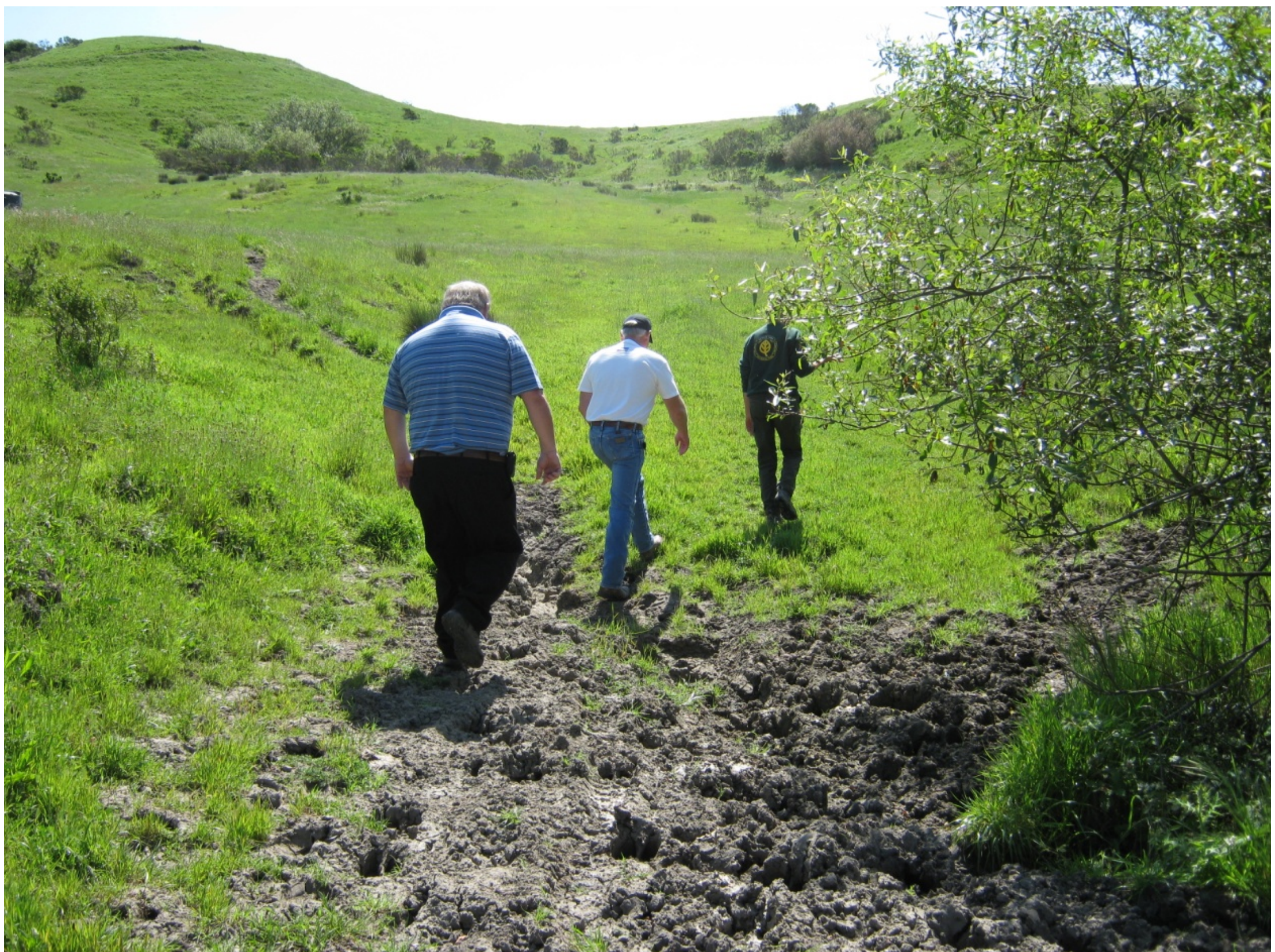

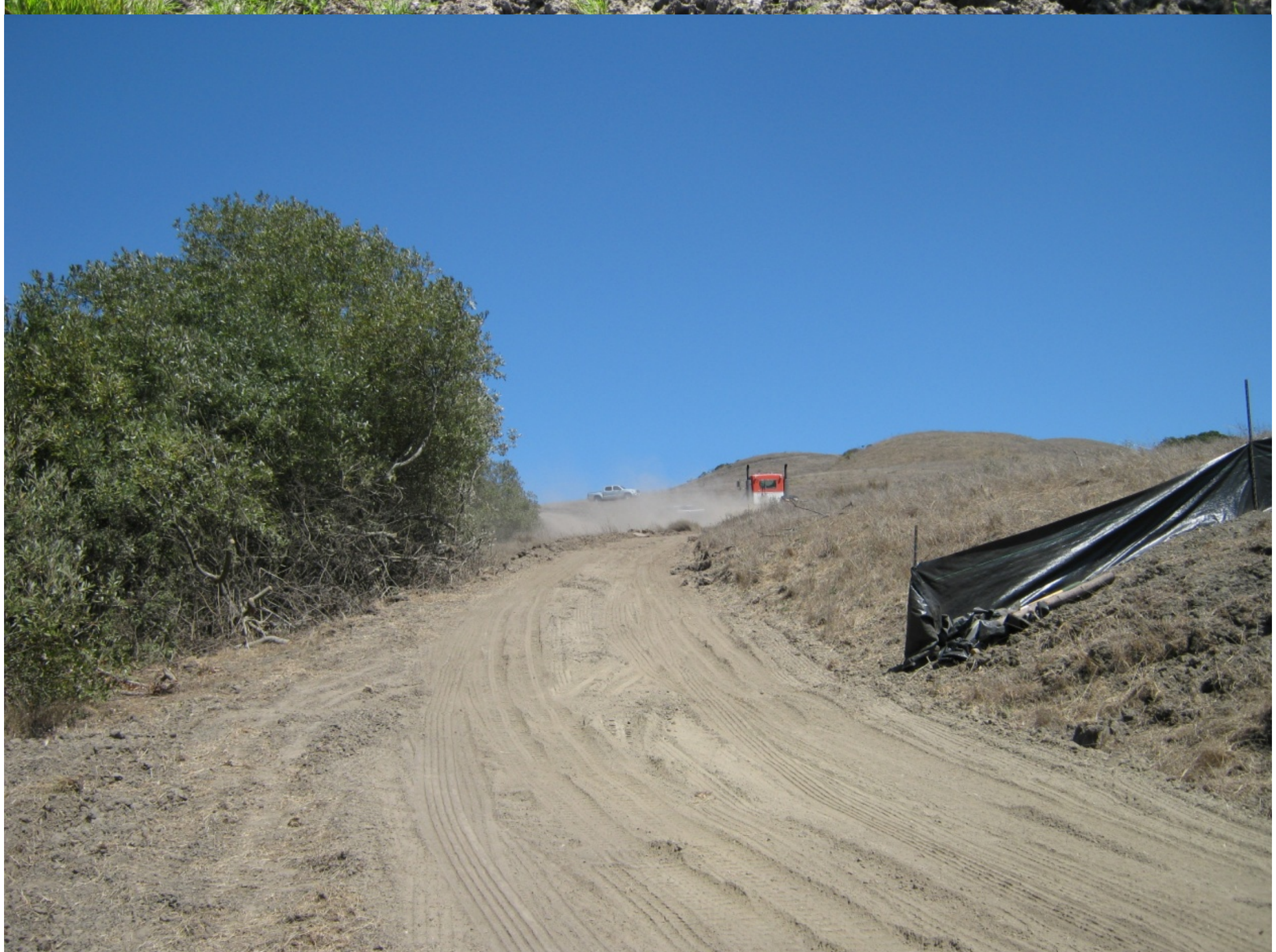

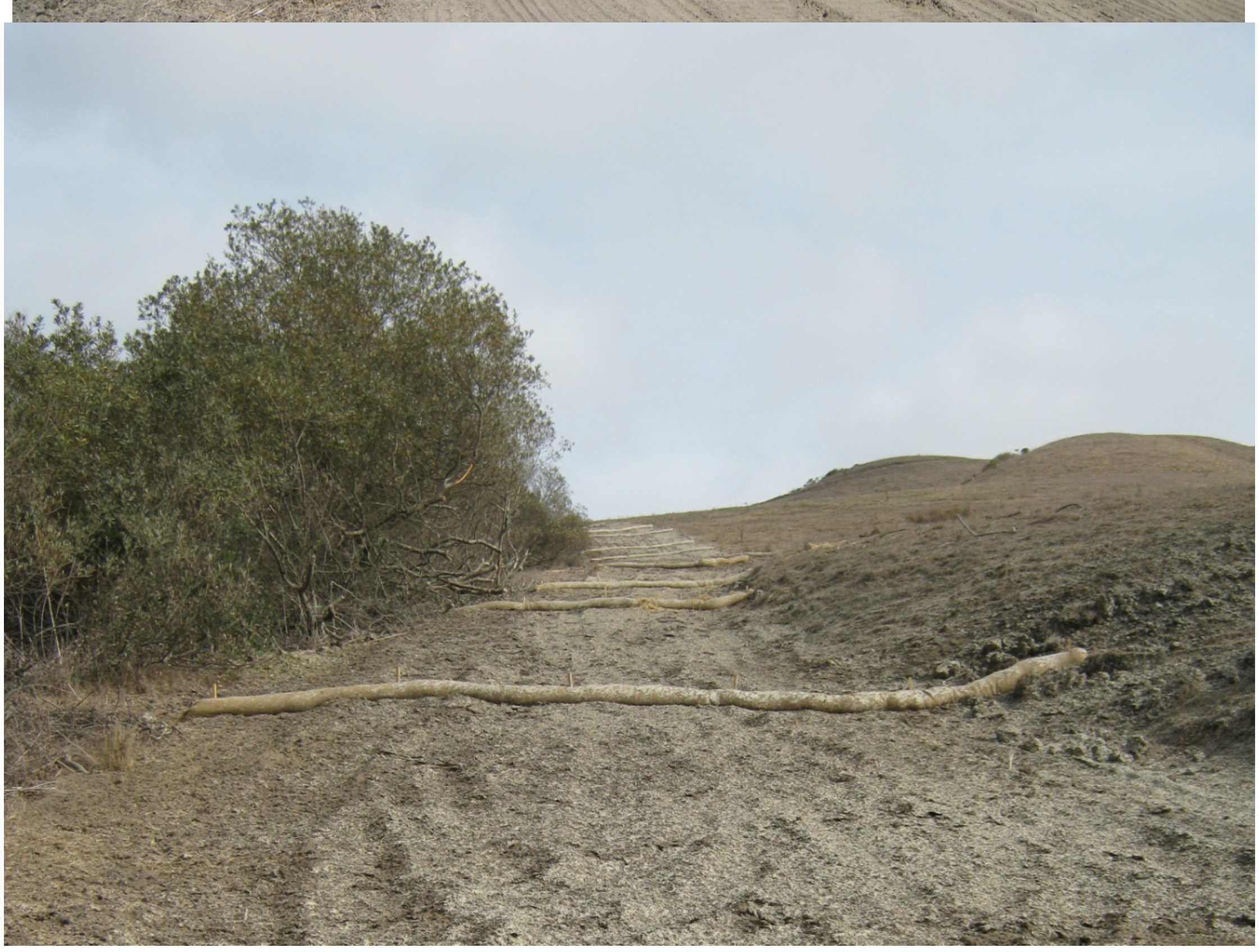

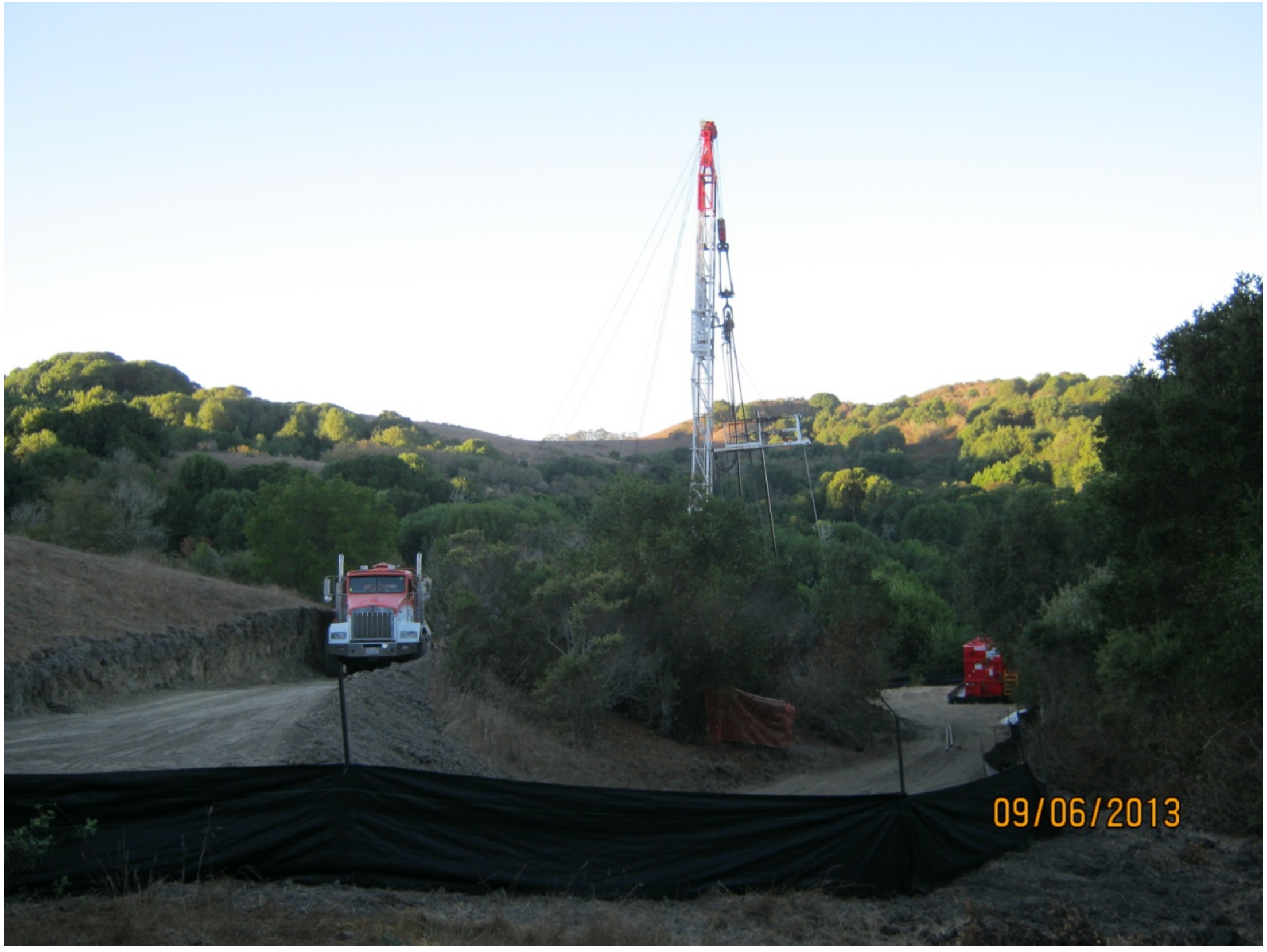
09/06/2013

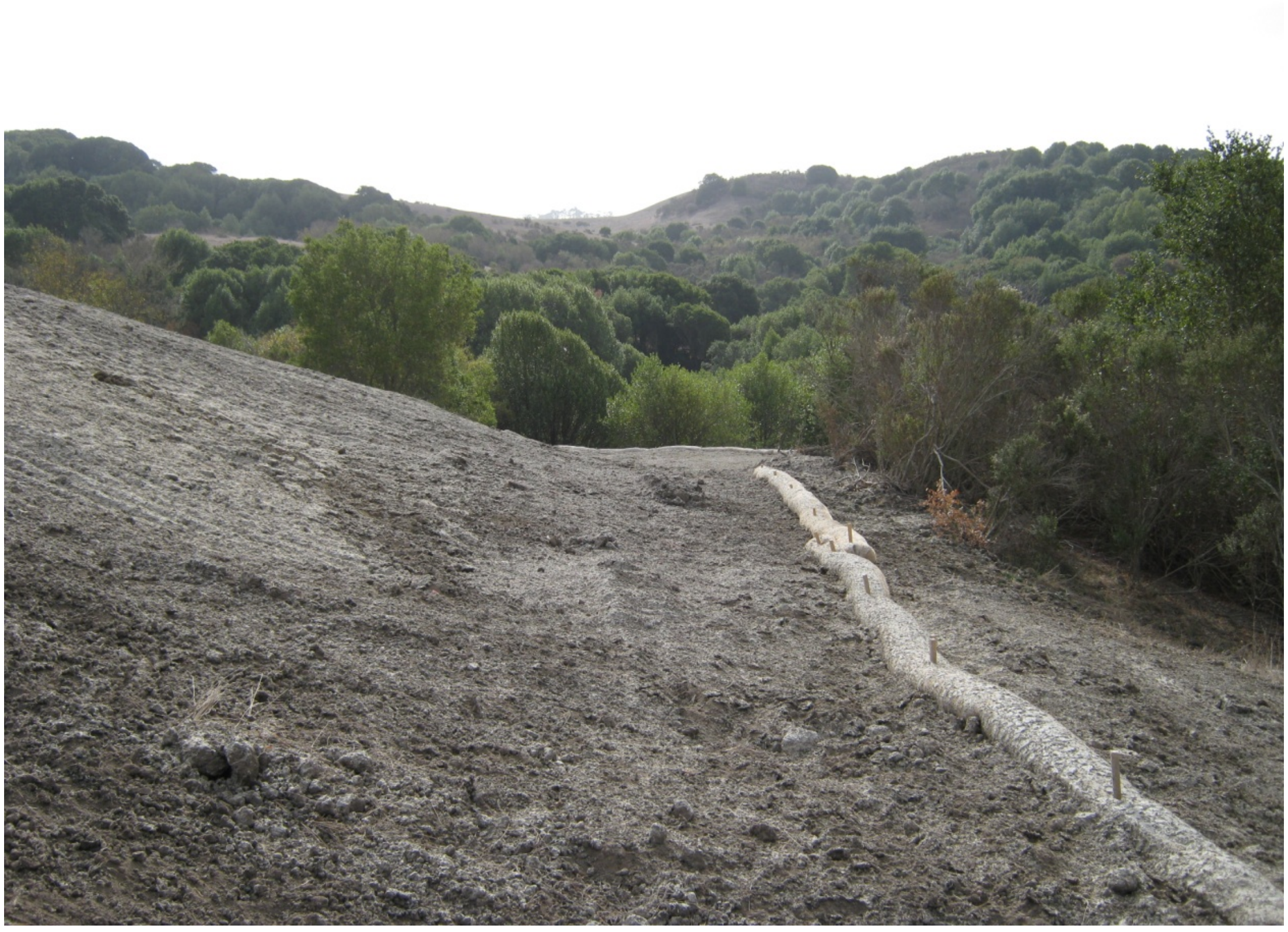

DIVISION OF OIL, GAS, AND GEOTHERMAL RESOURCES

CEMENTING/PLUGGING MEMO

Operator Unknown /Rival Well Services Well No. Unknown 1

API No. 01320381 Sec. 10 T. 1N R. 4W , MD B&M

Field ______ , County Contra Costa . On 9/13/2013 ,

Mr. BG Tackett representative of the supervisor, was present from 0430 to 2130 .

There were also present Mr. Bob Grayson, abandonment contractor

Casing record of well: No record exists for this well.

The operations were performed for the purpose of:

Abandonment

☒ The plugging/~~cementing~~ operations as witnessed and reported are approved.

☐ The location and hardness of the cement plug @ ______ ' are approved.

Hole size: ______ " fr. ______ ' to ______ ', ______ " to ______ ' & ______ " to ______ '

Casing				Cemented			Top of Fill		Squeezed Away	Final Pressure	Perforations
Size	Wt.	Top	Bottom	Date	MO-Depth	Volume	Annulus	Casing			
				9/11/2013	Dp @ 350	92	Unknown	316	82	0	360-362

Casing/tubing recovered: ______ " Shot/cut at ______ ', ______ ', ______ ', Pulled fr. ______ ';

______ " Shot/cut at ______ ', ______ ', ______ ', Pulled fr. ______ '.

Junk (in hole): ______

Hole fluid (bailed to) at ______ '. Witnessed by ______

Mudding	Date	bbls	Displaced	Poured	Fill	Engineer
**9/13/2013	23 cf	Dp @ 30'	Cem to sfc	vis		bgt

C/O depth 538' on 9/5/2013

Cement Plugs		Placing	Placing Witnessed		Top Witnessed			
Date	Sx./cf	MO & Depth	Time	Engineer	Depth	Wt/Sample	Date & Time	Engineer
9/10/2013	82 cf	dp 500	0430-2000	JAS	365	Tbg total	9/11/2013	JAS
					346	wireline	9/11/2013-1600	JAS
9/11/2013	92cf	Dp 318	1700	JAS	157	Dp total	9/12/2013	bgt
9/12/2013	26 cf	Dp 140	1400	bgt	30	Dp total	9/13/2013	Bgt **

RESOURCES AGENCY OF CALIFORNIA
DEPARTMENT OF CONSERVATION
DIVISION OF OIL, GAS, AND GEOTHERMAL RESOURCES

HISTORY OF OIL OR GAS WELL

Operator Rival Well Services, Inc. Field Wildcat Canyon County Contra Costa
Well "Unknown" #1 Sec. 10 T. 1N R. 4W M.D.B.&M.
A.P.I. No. ------ Name Bob Grayson Jr. (Person submitting report) Title President (President, Secretary, or Agent)
Date 11-6-13 (Month, day, year)
Signature [signature]
Address P.O. Box 12620, Bakersfield, CA 93389 Telephone Number 661 392-1700

History must be complete in all detail. Use this form to report all operations during drilling and testing of the well or during redrilling or altering the casing, plugging, or abandonment, with the dates thereof. Include such items as hole size, formation test details, amounts of cement used, top and bottom of plugs, perforation details, sidetracked junk, bailing tests, and initial production data.

Date 2013	
8-13	Prepared road and location for abandonment equipment.
8-22	Moved in equipment. MIRU.
8-23	Prepared well casing for welder to install API wellhead. Found casing full of bullet holes. Cut off casing 3 feet below grade. Welded on 3 ½' of 12" casing to extend casing to surface. SDFN.
8-24	Welder installed 12" API wellhead. Installed BOPE. RU workfloor. MU bit and bit sub on drill pipe. RIH and tagged down @ 25'. Secured well. SDFN.
8-26	LD bit and drill pipe. RD work floor. Rig down to install shoring around well casing. Backfilled around shoring. SDFN.
8-27	MIRU. RU work floor. MU bit and bit sub on drill pipe. RIH to 25'. RU power swivel and pump. Cleaned out from 25' to 90'. Hard drilling from 90' to 98'. Fell through from 98' to 101'. Circulated clean. Secured well. SDFN.
8-28	MU 4 ¾" flat bottom mill. RIH to 101'. Milled out wood from 101' to 118'. Secured well. SDFN.
8-29	Continued to mill out wood from 118' to 130'. Kept plugging circulating system with wood splinters. Fell past wood plug @ 131'. Secured well. SDFN.
8-30	RD power swivel. Pushed wood down hole from 131' to 300'. Unable to work past 300'. RU power swivel. Drilled out wood from 300' to 315'. Secured well. SDFN.
9-3	Cleaned out oil and wood from pump pit. Drilled out wood from 315' to 380'. Secured well. SDFN.

2013 NOV -4 AM 11:40

HISTORY OF OIL OR GAS WELL

9-4	Continued to drill out wood from 380' to 445'. Unable to make hole @ 445'. POOH and found mill was worn out. Replaced mill. RIH with mill and stacked out @ 367'. Drilled out wood from 367' to 380'. Secured well. SDFN.
9-5	Continued to drill out wood from 380' to 538'. Mill getting stuck @ 538' Worked mill free and POOH to 412'. Circulated clean. Cleaned wood chips out of valves on pump. Secured well. SDFN.
9-6	Resumed drilling out wood from 412' to 480'. Harder drilling from 480' to 522'. Mill became stuck @ 522'. Worked stuck mill free. POOH to 412. Secured well. SDFN.
9-9	RIH from 412' to 476'. Drilled out from 476' to 507'. Circulated clean. POOH to 412'. Found oil and water had surfaced below location and had run into creek bed. Moved in cementing equipment. Secured well. SDFN.
9-10	Met with DFG on spill response. Contracted spill clean up. RIH with O/E drill pipe to 500'. Mixed and pumped 82 cu ft neat cement. Displaced with water. POOH with drill pipe. Secured well. SDFN.
9-11	RIH and tagged TOC @ 365'. POOH with drill pipe. RU wireline and perforated 4 jhpf from 360' to 362'. RD wireline. RIH with drill pipe to 350'. RU cementing equipment. Mixed and pumped 92 cu ft neat cement. Displaced with water. POOH. WOC for 4 hrs. RIH with sandline and tagged wet cement @ 346'. RIH with drill pipe to 318'. Mixed and pumped 92 cu ft neat cement. Displaced with water. POOH. Secured well. SDFN.
9-12	RIH and tagged TOC @ 157'. Hung tubing @ 140'. RU cementing equipment. Mixed and pumped 92 cu ft neat cement. Mixed and pumped an additional 26 cu ft neat cement to surface. Removed BOPE. Loaded out tools. SDFN.
9-13	RIH and tagged TOC @ 31'. Mixed and pumped 23 cu ft neat cement from 31' to surface. RDMO. Removed shoring. Cut off casing 5' below grade. Welded on steel plate. Moved out equipment to staging area. SDFN.
9-16	Backfill and clean up location. Stockpile contaminated soil.
10-10	Hauled out contaminated soil and fluids.
10-16	Finished re-contouring location. Moved out equipment. Complete.

NATURAL RESOURCES AGENCY OF CALIFORNIA
DEPARTMENT OF CONSERVATION
DIVISION OF OIL, GAS & GEOTHERMAL RESOURCES
801 K Street, MS 20-22 Sacramento, CA 95814 - 3530

PERMIT TO CONDUCT WELL OPERATIONS

No. P 613-0081

**Old**	**New**	
00	--	FIELD CODE
00	--	AREA CODE
00	--	POOL CODE

Sacramento, California
May 23, 2013

Mr Bob Grayson, Jr, Contractor
Rival Well Services, Inc.
P.O. Box 12620
Bakersfield, CA 93389

Your proposal to **Abandon** well **"Unknown" 1**, A.P.I. No. **--------**, Section **10**, T. **1N**, R. **4W**, **MD** B. & M., **Wildcat Canyon** field, **--** area, **--** pool, **Contra Costa** County, dated **05/16/2013**, received **05/20/2013** has been examined in conjunction with records filed in this office.

THE PROPOSAL IS APPROVED PROVIDED:

1. All portions of the well not plugged with cement are filled with inert mud fluid having a minimum density of **72** lbs/cu.ft and a minimum gel shear strength of **20** lbs./100 sq. ft.
2. The well is cleaned out as deep as possible.
3. The well is plugged with cement from **cleanout depth to 5**', plus any remaining annular space.
4. No program changes are made without prior Division approval.
5. The Division is promptly notified of any blowouts, fires, serious accidents, or any significant gas or water leaks.

THIS DIVISION SHALL BE NOTIFIED TO:

1. Witness the cleanout of the well.
2. Witness the placement of cement from cleanout depth to surface.
3. If mudding of the well is required, witness the mudding operations.
4. Witness the location and hardness of cement at surface, including all annular spaces.
5. Inspect the restored well site.

NOTE:

1. All casing must be removed from between 5' and 10' below ground level.
2. Well site restoration shall be completed within 60 days following the completion of plugging operations.
3. The cleanup of the well site must be approved by the Division before the Report of Final Well Abandonment (OG159) can be issued. Please contact this office for site inspection after the well site cleanup work has been completed. **PURSUANT TO THE CALIFORNIA CODE OF REGULATIONS, WELL SITE RESTORATION MUST BE COMPLETED WITHIN 60 DAYS FOLLOWING PLUGGING AND ABANDONMENT OF THE WELL.**

Blanket Bond
Engineer Baxter Tackett

Tim Kustic, State Oil and Gas Supervisor

X *Michael L. Woods*

Michael L. Woods
District Deputy

Office (916) 322-1110
BT/sh

A copy of this permit and the proposal must be posted at the well site prior to commencing operations. Records for work done under this permit are due within 60 days after the work has been completed or the operations have been suspended. Issuance of this permit does not affect the Operator's responsibility to comply with other applicable state, federal, and local laws, regulations, and ordinances.

FOR DIVISION USE ONLY			
	Forms		
Bond	OGD114	OGD121	

NATURAL RESOURCES AGENCY OF CALIFORNIA
DEPARTMENT OF CONSERVATION
DIVISION OF OIL, GAS, AND GEOTHERMAL RESOURCES

NOTICE OF INTENTION TO ABANDON / RE-ABANDON WELL

Detailed instructions can be found at: www.conservation.ca.gov/dog

In compliance with Section 3229, Division 3, Public Resources Code, notice is hereby given that it is our intention to abandon ☒ / re-abandon ☐ (Check one) well "Unknown" 1, API No. N/A,

Sec.____, T.____, R.____, M.D. B.&M., Wildcat Canyon Field, Contra Costa County.

The complete casing record of the well (present hole), including plugs and perforations, is as follows: (Attach wellbore schematics diagram also.)

12" @ Unknown depth

Obstruction @ 24'

The total depth is: N/A feet. The effective depth is: N/A feet.

Present completion zone(s): N/A (Name). Present zone pressure: N/A psi.

Oil or gas shows: N/A (Name and depth) feet. Depth to base of fresh water: 320 feet.

Top of uppermost hydrocarbon zone (which may be behind unperforated casing): N/A (Depth of interval) feet.

Is this a critical well as defined in the California Code of Regulations, Title 14, Section 1720(a) (see next page)? Yes☐ No☒

The proposed work is as follows: (A complete program is preferred and may be attached.)

1. MIRU
2. Install wellhead and cellar ring.
3. Clean out as deep as possible.
4. Plug with cement from cleanout depth to 5'.
5. RDMO. Cut off casing 5feet below grade. Weld on steel plate.
6. Backfill location.

The Division must be notified immediately of changes to the proposed operations. Failure to provide a true and accurate representation of the well and proposed operations may cause rescission of the permit.

Name of Operator			
Rival Well Services Inc.			
Address<br>P.O. Box 12620		City/State<br>Bakersfield	Zip Code<br>93389
Name of Person Filing Notice<br>Bob Grayson Jr.	Telephone Number:<br>661 392-1700	Signature<br>[signature]	Date<br>May 16, 2013
Individual to contact for technical questions:<br>Bob Grayson Jr.	Telephone Number:<br>661 392-1700	E-Mail Address:<br>bobgrayson@rivalwellservices.com	

This notice must be filed, and approval given, before plugging and abandonment operations begin. If operations have not commenced within one year of the Division's receipt of the notice, this notice will be considered cancelled.
