# Supplementary material for: Locating Undocumented Wells Using Historical Oil and Gas Exploration Maps: A Case Study in Osage County, Oklahoma
Source: Environ Sci Technol. 2026 Jul 16;60(30):20913–24. doi: 10.1021/acs.est.6c03981 (PMC13449896; doi:10.1021/acs.est.6c03981)
Supplement: Supplementary file 3 [file es6c03981_si_004.pdf]

RESOURCES AGENCY OF CALIFORNIA
DEPARTMENT OF CONSERVATION
DIVISION OF OIL AND GAS

REPORT OF WELL ABANDONMENT

Long Beach, California

October 13, 1988

Fred T. Hifumi
KAISER FOUNDATION HEALTH PLAN
765 College Street
Los Angeles, CA 90026

Your report of abandonment of well "Unknown" 2 (Name and number),

A.P.I. No. None, Section 21, T. 1S, R. 13W, S.B. B. & M.,

Los Angeles City field, Los Angeles County,

dated June 2, 1988, received June 3, 1988, has been examined in conjunction with records filed in this office, and we have determined that all of the requirements of this Division have been fulfilled.

MJK:est

cc: Update
Conservation Committee
EDP
Environmental Management Agency Regulation - Mike Matkosky
Dept. of Oilfield Control

NO BOND REQUIRED

M. G. MEFFERD
State Oil and Gas Supervisor

By [signature]
Deputy Supervisor

for: R. K. Baker

OG159 (11/82/GSR1/8M)

DIVISION OF OIL AND GAS
CHECK LIST - RECORDS RECEIVED AND WELL STATUS

Company KAISER FOUNDATION HEALTH PL Well No. "UNKNOWN" 2
API No. NONE Sec. 21, T. 1S, R. 13W, S.B. B.&M.
County LOS ANGELES Field LOS ANGELES CITY

RECORDS RECEIVED	DATE	
Well Summary (Form OG100)		
History (Form OG103)	6-3-88	2
Core Record (Form OG101)		
Directional Survey		
Sidewall Samples		
Other		
Date final records received		
Electric logs:		

STATUS		STATUS	
Producing - Oil		Water Disposal	
Idle - Oil		Waterflood	
Abandoned - Oil	X	Steamflood	
Drilling - Idle		Fire Flood	
Abandoned - Dry Hole		Air Injection	
Producing - Gas		Gas Injection	
Idle - Gas		CO_2 Injection	
Abandoned - Gas		LPG Injection	
Gas-Open to Oil Zone		Observation	
Gas Storage		Waterflood Source	

DATE ____ "E" well Yes ☐ No ☐
RECOMPLETED 6-1-88
REMARKS

ENGINEER'S CHECK LIST

1. Summary, History, & Core record (dupl.) ✓✓
2. Electric Log ✓
3. Operator's Name ?
4. Signature ?
5. Well Designation ✓
6. Location ✓
7. Elevation —
8. Notices ✓
9. "T" Reports ✓✓
10. Casing Record ✓
11. Plugs ✓
12. Surface Inspection NEED
13. Production
14. E Well on Prod., enter EDP
15. Directional Survey

CLERICAL CHECK LIST

1. Location change (F-OGD165)
2. Elevation change (F-OGD165)
3. Form OGD121 10-13-88 et
4. Form OG159 (Final Letter) 10-13-88 et
5. Form OGD150b (Release of Bond)
6. Duplicate logs to archives
7. Notice of Records due (F-OGD170)
8. EDP (F-OGD42A-1, 2)

FINAL LETTER

SURFACE INSPECTION 10/13/88 TR
Surf. inspection approved

MAP 10/13/88 TR
Map #116

RECORDS NOT APPROVED
Reason:

RECORDS APPROVED [signature]
RELEASE BOND N/A
Date Eligible
(Use date last needed records were received.)
MAP AND MAP BOOK Map # 116
T. Ramsey 10/13/88

LONG BEACH OFFICE - USE REVERSE SIDE

OGD2 (8/87/DWRR/5M)

FILE

SUBMIT IN DUPLICATE

RESOURCES AGENCY OF CALIFORNIA
DEPARTMENT OF CONSERVATION

DIVISION OF OIL AND GAS

History of Oil or Gas Well

Operator Kaiser Foundation Health Plan Field Los Angeles City County Los Angeles
Well "Unknown" 2, Sec. 21, T 1S, R 13W, SB B. & M.
A.P.I. No. ------------ Name Gary F. Cook (Person submitting report) Title Vice President (President, Secretary or Agent)
Date 6/2, 19 88

Signature [signature]

Alco Well Service, Inc.
15117 Minnesota Ave. Paramount, Calif. 90723 (Address) 213 - 630-0901 (Telephone Number)

History must be complete in all detail. Use this form to report all operations during drilling and testing of the well or during redrilling or altering the casing, plugging, or abandonment with the dates thereof. Include such items as hole size, formation test details, amounts of cement used, top and bottom of plugs, perforation details, sidetracked junk, bailing tests and initial production data.

Date

ABANDONMENT

5/20/88 Excavated well area, installed portable cellar & welded on flange. Rigged up & installed B.O.P. R.I.H. with 6-1/8" bit to 246'.

5/23/88 Continued to clean out to 616' - lost circulation - fluid returns outside casing @ surface; P.O.H. R.I.H. with 9-5/8" cup on tubing & tested casing - found hole @ approximately 35'; P.O.H. Note: well casing measured 10" O.D. & 9-5/8" I.D. Mixed & pumped 17 sacks cement from surface - no cement returns outside casing @ surface.

5/24/88 R.I.H. with tubing - did not tag cement; P.O.H. Pressure test casing - no good - returns outside casing @ surface. Mixed & pumped 35 sacks cement from surface. W.O.C. Test casing - good.

5/25/88 R.I.H. with 6-1/8" bit to 610' - cleaned out to 826' - stopped hard; OK per D.O.G. P.O.H. Run collar survey - indicated last casing collar @ 320'; OK per D.O.G. R.I.H. with tubing to 824' - mixed & pumped 111 sacks cement with 4% gel - squeezed with 50 cu.ft.; OK per D.O.G.

5/26/88 Tagged plug @ 651'; OK per D.O.G. Set tubing @ 649' - mixed & pumped 105 sacks cement with 4% gel - squeezed with 50 cu.ft.; OK per D.O.G. Tagged plug @ 419'; OK per D.O.G. Set tubing @ 417' - mixed & pumped 70 sacks cement with 4% gel & 4% CaCl - squeezed with 60 cu.ft. W.O.C. Tagged plug @ 281'; OK per D.O.G. Shot cavity @ 275'. Set tubing @ 279' - mixed & pumped 70 sacks cement with 4% gel & 4% CaCl - squeezed with 60 cu.ft.; OK per D.O.G.

5/27/88 Tagged plug @ 133' - too high; witnessed by D.O.G. Drilled out cement to 160'; OK per D.O.G. Shot cavity @ 150'. Set tubing @ 158' - mixed & pumped total of 75 sacks cement - cement returns inside casing after pumping 72 sacks cement - squeezed 3 sacks @ 250 p.s.i.; OK per D.O.G.

6/ 1/88 Cut off casing 18' below surface & welded on labeled top plate.

RECEIVED
88 JUN -3 AM 8:09
DIVISION OF OIL & GAS
LONG BEACH, CA

ABANDONMENT COMPLETE

RECEIVED
88 JUN -3 PM 2:09
DIVISION OF OIL & GAS
LONG BEACH, CA

RESOURCES AGENCY OF CALIFORNIA
DEPARTMENT OF CONSERVATION

No. T 188-944

DIVISION OF OIL AND GAS

Report on Operations

Fred T. Hifumi
KAISER FOUNDATION HEALTH PLAN
765 College Street
Los Angeles, CA 90026

Long Beach, Calif.
September 23, 1988

Your operations at well "Unknown" 2, API No. None,
Sec. 21, T. 1S R. 13W, S.B. B.& M. Los Angeles City Field, in Los Angeles County,
were witnessed on 5-27-88. M. Saw, Engineer, representative of
the supervisor, was present from 1400 to 1630. There were also present R. Branch, Driller

Present condition of well: 9-5/8" cem 322'? Hole 35' (cem off), cavity shot 270' & 150'. TD unknown. Plugged w/ cem 826-6'.

The operations were performed for the purpose of witnessing the plugging operations in the process of abandonment.

DECISION: APPROVED.

NOTE: DEFICIENCIES CORRECTED
NONE

DEFICIENCIES TO BE CORRECTED
NONE

UNCORRECTABLE DEFICIENCIES
NONE

CONTRACTOR: Alco Well Service, Inc.

surface inspection approved T. Ramsey 10/13/88

MS:mh

cc: Update
EDP

M. G. MEFFERD
State Oil and Gas Supervisor
By [signature]
Deputy Supervisor
for R. K. Baker

OG109 (3/83/GSR1/15M)

#O 6/27/88 188-944

DIVISION OF OIL AND GAS
Cementing/Plugging Memo

Operator Kaiser Foundation Health Pl. Well No. "Unknown" 2
API No. None Sec. 21, T. 1S, R. 13W, S.B. B&M
Field L.A. City, County L.A. On 5/27/88,
Mr. M. Saw, representative of the supervisor was present from 1400 to 1630.
There were also present Mr. R. Branch, Driller

Casing record of well: 9 5/8" cem 322?, Hole 35' (cem off), Cavity shot 270' & 150'. TD ~~826'~~ unknown. Plugged w/ cem 826'-6'.

The operations were performed for the purpose of (L)

[x] The plugging/~~cementing~~ operations as witnessed and reported are approved.

[ ] The location and hardness of the cement plug @ ______' is approved.

Hole size: ____" fr. ____' to ____', ____" to ____' & ____" to ____'

Casing Size	Wt.	Top	Bottom (see below) (+)	Cemented Date	MO-Depth	Volume	Top of Fill Annulus	Top of Fill Casing	Squeezed Away	Final Press.	Perfs.
9 5/8"	?	0	322'	5/24	Down squeezed fr the surface	111 SX	—	651'	50 cf	0	—
"	?	0	322'	5/26	" "	70 SX	—	133'	65 cf	0	Cav shot 270'
"	?	0	322'	5/27	" "	75 SX	—	6'	5 cf	250	Cav. shot 150'

hole @ 35' ±, cav. shot 270' & 150'.

Casing/tubing recovered: ____" shot/cut at ____', ____', ____' pulled fr. ____';
____" shot/cut at ____', ____', ____' pulled fr. ____'.

Junk (in hole):

Hole fluid (bailed to) at ____'. Witnessed by

Mudding	Date	Bbls.	Displaced	Poured	Fill	Engr.
— Cement from c/o to surface —						

Cement Plugs Date	Sx./cf	Placing MO & Depth	Placing Witnessed Time	Engr.	Top Witnessed Depth	Wt/Sample	Date & Time	Engr.
5/25	111 SX	tbg @ 826'	1700	Apvd by J. Swanson	651'	Low	5/26 0740	M. Saw
5/26	105 SX	" " 650'	1100	" "	419'	Low	5/26 1310	M. Saw
5/26	70 SX	" " 419	1330	" "	281'	all tbg wt	5/26 1710	M. Saw
5/26	70 SX	" " 281'	2000	" "	133*	High	5/27 0800	M. Saw
5/27	70 SX	" " 160'	1600	M. Saw	6'	visual	5/27 1630	M. Saw

* drilled down to 160'.

all the cement was mixed with 4% gel + 4% $CaCl_2$.

OGD10 (10/85/DWRR/5M)

DHH - C/O 826' (w/ 6 1/4" bit); (+) 9 5/8" csg shoe @ 322' (Tiger Wireline)

DEFICIENCIES NOTED AND CORRECTED:

NONE

DEFICIENCIES NOTED AND TO BE CORRECTED:

NONE

UNCORRECTABLE DEFICIENCIES:

NONE

CONTRACTOR:

ALCO WELL SERVICE, INC.

RESOURCES AGENCY OF CALIFORNIA
DEPARTMENT OF CONSERVATION

No. T 188-943

DIVISION OF OIL AND GAS

Report on Operations

Fred T. Hifumi
KAISER FOUNDATION HEALTH PLAN
765 College Street
Los Angeles, CA 90026

Long Beach, Calif.
September 23, 1988

Your operations at well "Unknown" 2, API No. None, Sec. 21, T1S, R.13W, S.B. B.& M. Los Angeles City Field, in Los Angeles County, were witnessed on 5-25-88. B. Hesson, Engineer, representative of the supervisor, was present from 1430 to 1730. There were also present R. Branch, Drilling Foreman

Present condition of well: 9-5/8" cem 322'? TD unknown.

The operations were performed for the purpose of inspecting the blowout prevention equipment and installation.

DECISION: APPROVED.

NOTE: DEFICIENCIES CORRECTED
NONE

DEFICIENCIES TO BE CORRECTED
NONE

UNCORRECTABLE DEFICIENCIES
NONE

CONTRACTOR: Alco Well Service, Inc.

surface inspection approved 10/13/88 TRamsey

BH:mh

cc: Update

M. G. MEFFERD
State Oil and Gas Supervisor
By [signature]
Deputy Supervisor
for R. K. Baker

OG109 (3/83/GSR1/15M)

#0 6/27/88

DIVISION OF OIL AND GAS

BLOWOUT PREVENTION EQUIPMENT MEMO

T 188-943

Operator KAISER FOUNDATION HEALTH PL. Well "UNKNOWN" 2 Field L.A. CITY County L.A.

VISITS:	Date	Engineer	Time	Operator's Rep.	Title
1st	5/25/88	B. HESSON	1430 to 1730	R. BRANCH	D.F.
2nd			to		

Casing record of well: 9 5/8" CEM 322? TD ~~826~~' unknown.

OPERATION: ~~Testing~~ (inspecting) the blowout prevention equipment and installation.
DECISION: The blowout prevention equipment and its installation on the 9 5/8 "casing are approved.

REQUIRED BOPE CLASS: II 2M

Proposed Well Opns: ABANDON MPSP: ______ psi

Hole size: ____" fr. ____' to ____', ____" to ____' & ____" to ____

CASING RECORD (BOPE ANCHOR STRING ONLY)					Cement Details	Top of Cement	
Size	Weight(s)	Grade(s)	Shoe at	CP at		Casing	Annulus

BOP STACK							a	b	a/b	TEST DATA			
API Symb.	Ram Sz.	Mfr.	Model or Type	Size (in.)	Press. Rtg.	Date Last Overhaul	Gal. to Close	Rec.Time (Min.)	Calc. GPM Output	psi Drop to Close	Secs. to Close	Test Date	Test Press.
Rd	2 7/8	SHAFFER	MECH.	12	2000								
Rd	CSO	"	"	"	"								

ACTUATING SYSTEM

Accum. Unit(s) Wkg. Press. ______ psi
Total Rated Pump Output ______ gpm
Distance From Well Bore ______ ft.

	Mfr.	Accum. Cap.	Precharge
1		gal.	psi
2		gal.	psi

CONTROL STATIONS	Elec.	Hyd.
Manif. at accum. unit		
Remote at Drlr's. stn.		
✓ Other: HAND WHEEL		

EMERG. BACKUP SYST.		Press.	Wkg. Fl.
N2 Cyl No: Type:		1	gal.
Other:		2	gal.
		3	gal.
		4	gal.
		5	gal.
		6	gal.

AUXILIARY EQUIPMENT		No.	Sz. (in.)	Rated Press.	Weld	Flan.	Thrd.
	Fill-Up Line						
✓	Kill Line		2"	2000			✓
✓	Control Valve(s)	1		2000			✓
	Check Valve(s)						
	Auxil. Pump Connec.						
	Choke Line						
	Control Valve(s)						
	Pressure Gauge						
	Adjustable Choke(s)						
	Bleed Line						
	Upper Kelly Cock						
	Lower Kelly Cock						
	Standpipe Valve						
	Standpipe Pressure Ga.						
✓	Pipe Safety Valve		2 7/8"				
	Internal Preventer						

HOLE FLUID MONITORING EQUIPMENT	Alarm Aud.	Alarm Vis.	Class
Calibrated Mud Pit			A
Pit Level Indicator			B
Pump Stroke Counter			B
Pit Level Recorder			C
Flow Sensor			C
Mud Totalizer			C
Calibrated Trip Tank			C
Other:			

REMARKS:

Hole Fluid Type	Weight	Storage Pits
CB MUD	72 PCF	200± BBLS

OGD9 (10/85/DWRR/3M)

DEFICIENCIES NOTED AND CORRECTED:

NONE

DEFICIENCIES NOTED AND TO BE CORRECTED:

NONE

UNCORRECTABLE DEFICIENCIES:

NONE

CONTRACTOR:

ALCO WELL SERVICE, INC.

RESOURCES AGENCY OF CALIFORNIA
DEPARTMENT OF CONSERVATION
DIVISION OF OIL AND GAS

No. P 188-420

422
(field code)
00
(area code)
--
(new pool code)
--
(old pool code)

PERMIT TO CONDUCT WELL OPERATIONS

Fred T. Hifumi
KAISER FOUNDATION HEALTH PLAN, INC.
765 College Street
Los Angeles, CA 90026

Long Beach, California
May 4, 1988

Your proposal to abandon well Kaiser Foundation Health Plan/ "Unknown" 2,
A.P.I. No. --, Section 21, T. 1S, R. 13W, S.B. B. & M.,
Los Angeles City field, -- area, -- pool,
Los Angeles County, dated 4-25-88, received 4-25-88 has been examined in conjunction with records filed in this office.

THE PROPOSAL IS APPROVED PROVIDED:

1. The hole must be cleaned out by circulating as deep as possible to determine the actual depth of the well.
2. This division shall be consulted if well casing is detached during clean-out.
c. THIS DIVISION SHALL BE NOTIFIED TO:
   a. Witness the cleaned out depth.
   b. Determine plug requirements according to the approved cleaned out depth.

NOTE:

1. Cement plugs shall not contain rock or gravel.
2. The base of the freshwater sands is at 150'±.
3. This division does not pass upon your right to enter the property, but merely approves the proposal as conforming to our requirements.
4. T-Reports (OG 109) and Report of Well Abandonment ((OG 159) (Final Letter) will NOT be released until all records and histories are on file with this division.

HRO:mh

cc: Update

NO BOND REQUIRED

Engineer Herman R. Olilang

Phone (213) 590-5311

M. G. MEFFERD, State Oil and Gas Supervisor

By R. C. Manuel
for R. K. Baker, *Deputy Supervisor*

A copy of this permit and the proposal must be posted at the well site prior to commencing operations.

Records for work done under this permit are due within 60 days after the work has been completed or the operations have been suspended.

OG111 (12/87/DWRR/5M)

STATE OF CALIFORNIA
DEPARTMENT OF CONSERVATION

DIVISION OF OIL AND GAS

APR 20 1988

Notice of Intention to Abandon Well

FOR DIVISION USE ONLY			
CARDS	BOND	FORMS	
		OGD114	OGD121
8B 4-26-88	NONE	8B 4-26-88	8B 4-26-88

DIVISION OF OIL AND GAS

In compliance with Section 3229, Division 3, Public Resources Code, notice is hereby given that it is our intention to abandon well "Unknown" 2, API No. ~~Unknown~~, Sec. 21, T. 1S, R. 13W, SB B. & M., Los Angeles City Field, Los Angeles County.

commencing work on ____________, 19 88.

The present condition of the well is:

1. Total depth ?
2. Complete casing record, including plugs and perforations (present hole) Unknown
3. Last produced Unknown (Date) (Oil, B/D) (Gas, Mcf/D) (Water, B/D)

or

4. Last injected Unknown (Date) (Water, B/D) (Gas, Mcf/D) (Surface pressure)

Additional data for dry hole (show depths):

5. Oil or gas shows
6. Stratigraphic markers
7. Formation and age at total depth
8. Base of fresh water sands 150'±

9. Is this a critical well according to the definition on the reverse side of this form? [X] Yes [ ] No

The proposed work is as follows: See attached report

Location: Fr. C/L College St & Figueroa Terrace
223' E & 233' N.

It is understood that if changes in this plan become necessary, we are to notify you immediately.

Address 765 College St. (Street)
Los Angeles, Calif. (City) (State) 90026 (Zip)
Telephone Number (213) 250-8609 (Area Code) (Number)

Kaiser Foundation Health Plan, Inc. (Name of Operator)
By Fred T. Hifumi (Print Name)
[signature] (Signature) Apr. 25, 1988 (Date)

CRITICAL WELL

As defined in the California Administrative Code, Title 14, Section 1720(a), "Critical well" means a well within:

(1) 300 feet of the following:
  (A) Any building intended for human occupancy that is not necessary to the operation of the well; or
  (B) Any airport runway.

(2) 100 feet of the following:
  (A) Any dedicated public street, highway, or nearest rail of an operating railway that is in general use;
  (B) Any navigable body of water or watercourse perennially covered by water;
  (C) Any public recreational facility such as a golf course, amusement park, picnic ground, campground, or any other area of periodic high-density population; or
  (D) Any officially recognized wildlife preserve.

Exceptions or additions to this definition may be established by the supervisor upon his own judgment or upon written request of an operator. This written request shall contain justification for such an exception.

RECEIVED
APR 27 9 17 AM '88
DIV OF OIL AND GAS
LONG BEACH, CA.

LeROY CRANDALL AND ASSOCI geotechnical consultants □ p.o. box 25088 □ 9 ind central ave. □ glendale, ca. 91201-3009
a subsidiary of Law Engineering facsimile (818) 24[illegible] telephone (818) 243-4140

RECEIVED BY
APR 0 7 1988
FAC. DESIGN. & CONST.

April 1, 1988

Kaiser Permanente
Facilities Design & Construction
393 Walnut Street, 3rd Floor
Pasadena, California 91188

Kaiser Project. No. MLF 483
(Our Job No. B-87284)

Attention: Mr. Fred Hifumi

Gentlemen:

Oil Well Abandonment Recommendations
Kaiser Mental Health Facility
765 College Street
Los Angeles, California

Our office is currently providing inspection services during grading of the subject site. In the course of grading operations, two open holes, approximately ten inches in diameter, were discovered below buried debris at the site. The open holes appear to be abandoned oil wells. The California Division of Oil and Gas (DOG) was contacted regarding the oil wells. The wells were measured to be approximately 400 and 750 feet deep. We have been asked to provide recommendations for abandonment of the wells.

The professional opinions presented in this letter have been developed using that degree of care and skill ordinarily exercised, under similar circumstances, by reputable geologists practicing in this or similar localities. No other warranty, expressed or implied, is made as to the professional advice included in this letter.

APR 2 9 17 AM '88
DIV O[illegible] AND GAS
[illegible] CH, CA

BACKGROUND

The site is located in the Elysian Hills, at the east end of the Los Angeles City Oil Field. The site is underlain by non-water bearing Puente Formation bedrock and is outside the designated ground water basin. The Los Angeles City Oil Field was developed near the turn of the century and there are many unrecorded oil wells within the area. It is our understanding that the DOG has no records regarding the encountered wells.

On March 24, 1988, we met Mr. Fred Hifumi of Kaiser and Ms. Trish Ramsey, oil and gas technician with the DOG at the site to inspect the exposed wells. The open holes were monitored with an explosimeter and a Foxboro organic vapor analyzer (OVA). The explosimeter indicated explosive levels of gas were present (greater than 100% LEL) and the OVA indicated that greater than 1,000 ppm methane (maximum reading) were also present within the wells near the ground surface.

The well locations were subsequently surveyed by a licensed surveyor to determine their locations in relation to the proposed buildings. Their locations are shown on the attached Site Plan.

RECOMMENDATIONS

We recommend that the exposed wells be abandoned by filling with concrete and providing a vapor vent above them. We have attached a diagramatic vent design provided by the DOG. Abandonment procedures should be performed as follows:

RECEIVED
APR 25 9 17 AM '88
DIV. OF OIL AND GAS
LONG BEACH, CA.

1. An area, a minimum of four feet by four feet, should be excavated around the well to a depth of eight to ten feet below planned finished grade.
2. The entire open oil well hole should be filled with either five or six sack concrete up to the bottom of the excavation or top of well casing to act as a surface plug.
3. If well casing is exposed in the excavation, a steel plate (minimum thickness equal to the casing) should be welded as an air-tight top to the well casing, two feet above the bottom of the excavation.
4. The excavation should be backfilled with five feet of 3/4" crushed rock.
5. A minimum of 6-mil plastic sheeting should be placed over the gravel and lapped downward, six inches, to act as a partial vapor trap and prevent the gravel from being clogged with fine grained soil.
6. A three-inch-diameter vent pipe constructed of Schedule 80 PVC or steel should be placed to protrude through the plastic covering, six to eight inches into the gravel, and extend up and over the adjacent planned buildings.
7. A minimum of three feet of cohesive (clayey or silty) compacted fill should be placed over the gravel, to the finished ground surface.

-oOo-

RECEIVED
APR 25 9 17 AM '88
DIV. OF OIL AND GAS
LONG BEACH, CA.

If you have any questions regarding information contained in this report, please do not hesitate to contact our office.

Respectfully submitted,

LeROY CRANDALL AND ASSOCIATES

by
Keith Farrell, C.E.G. 1314
Project Engineering Geologist

by
Mervin E. Johnson, C.E.G. 26
Vice President
Director of Geological Services

YC18/sle
Attachments (2)
(5 copies submitted)

RECEIVED
APR 25 9 17 AM '88
DIV. OF OIL AND GAS
LONG BEACH, CA.

DIAGRAMATIC VENT DESIGN

FOR OIL AND GAS WELLS LOCATED UNDER STRUCTURES

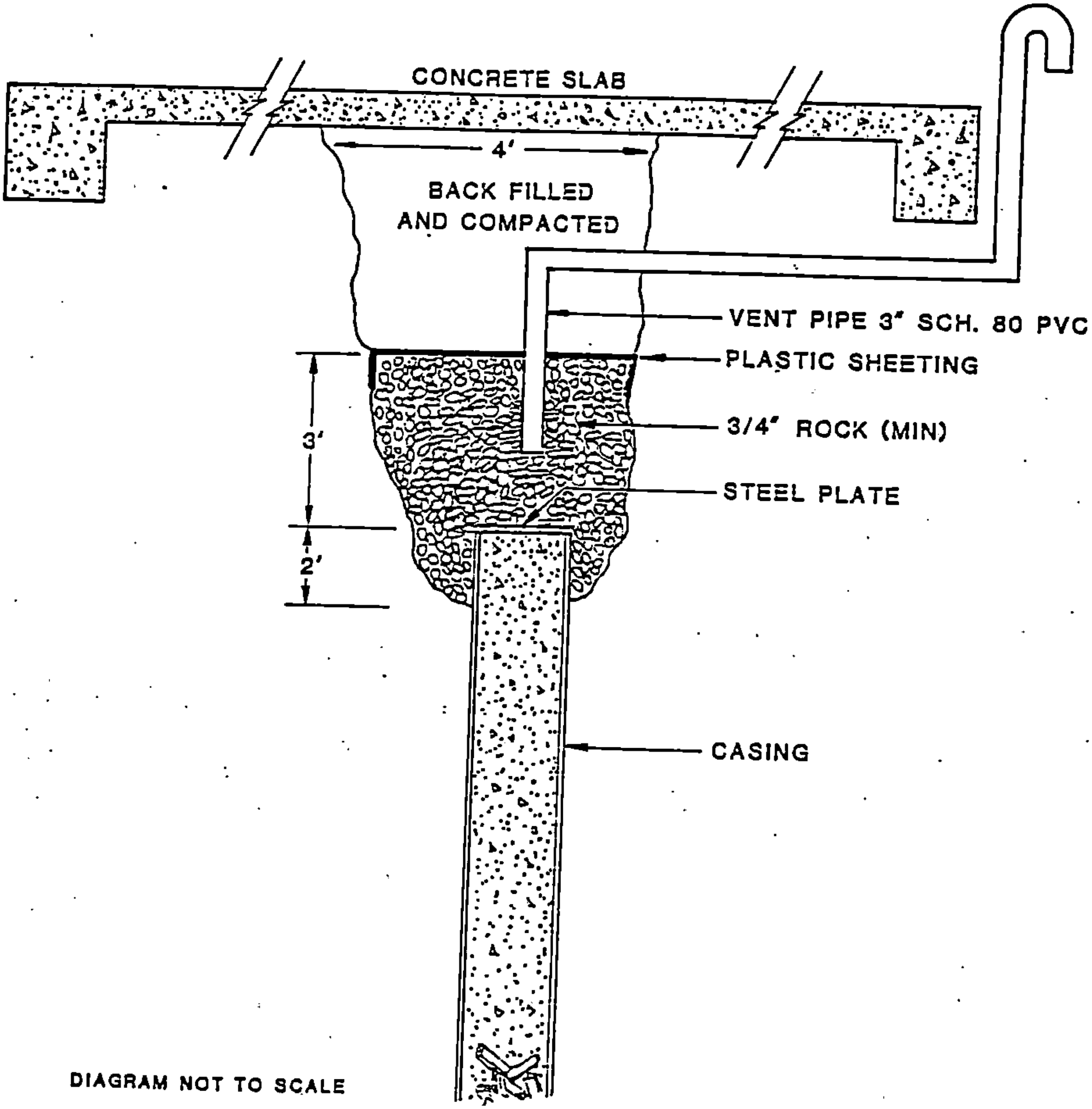

DIAGRAM NOT TO SCALE

RECEIVED
APR 25 9 17 AM '88
DIV. OF OIL AND GAS
LONG BEACH, CA.

FORM 137 JOB B-87284 DATE 4-4-88 DR. GARY T. O.E. ______ CHKD. ______

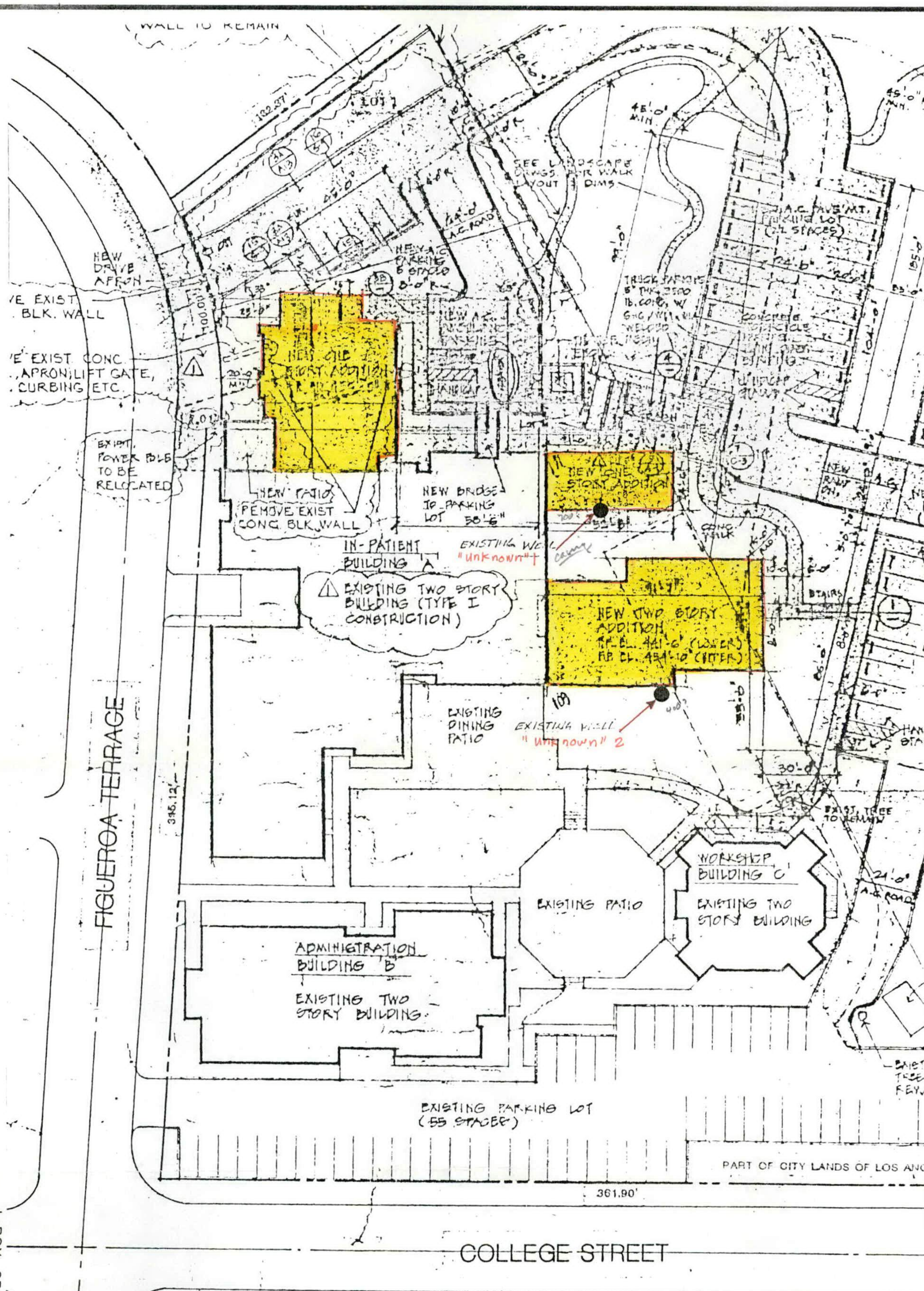

REFERENCE:

SITE PLAN - KAISER MENTAL HEALTH FACILITY BUILDING "A" BY A.P.&T. ARCHITECTS, REVISED 3 - 29 - 88

KEY:

● OIL WELL LOCATION (ABANDONED)

OIL WELL LOCATION PLAN

KAISER MENTAL HEALTH FACILITY - LOS ANGELES

SCALE 1" = 40'

LeROY CRANDALL AND ASSOCIATES

PLATE 1

KAISER PERMANENTE

DEPARTMENT OF FACILITIES PLANNING
1515 NORTH VERMONT AVENUE - SUITE 503
LOS ANGELES, CALIFORNIA 90027 (213) 667-6835

LETTER OF TRANSMITTAL

DATE April 26, 1988	JOB NO.
ATTENTION Herman Olilang	
PROJECT: Kaiser Mental Health Center	
RE: Abandonment of Existing wells	

TO: State of California
Department of Conservation
Division Of Oil and Gas
245 West Broadway, Suite 475
Long Beach, CA 90802-4455

GENTLEMEN:

WE ARE SENDING YOU [X] Attached [ ] Under separate cover via ______ the following items:

[ ] Shop drawings [ ] Prints [ ] Plans [ ] Samples [ ] Specifications

[ ] Copy of letter [ ] Change order [ ] ______

COPIES	DATE	NO.	DESCRIPTION
3	4/26/88		Site Plan indicating locations of existing wells per your request of this date. Also noted are approximate depths of the two wells.

THESE ARE TRANSMITTED as checked below:

[ ] For approval [ ] Approved as submitted [ ] Resubmit ____ copies for approval

[ ] For your use [ ] Approved as noted [ ] Submit ____ copies for distribution

[X] As requested [ ] Returned for corrections [ ] Return ____ corrected prints

[ ] For review and comment [ ] ______

[ ] FOR BIDS DUE ______ 19____ [ ] PRINTS RETURNED AFTER LOAN TO US

REMARKS ______

COPY TO ______

If enclosures are not as noted, kindly notify us at once.

SIGNED: Fred T. Hifumi

NS-5275 (5-83)

RECEIVED
APR 29 1 51 PM '88
DIV. OF OIL AND GAS
LONG BEACH, CA.

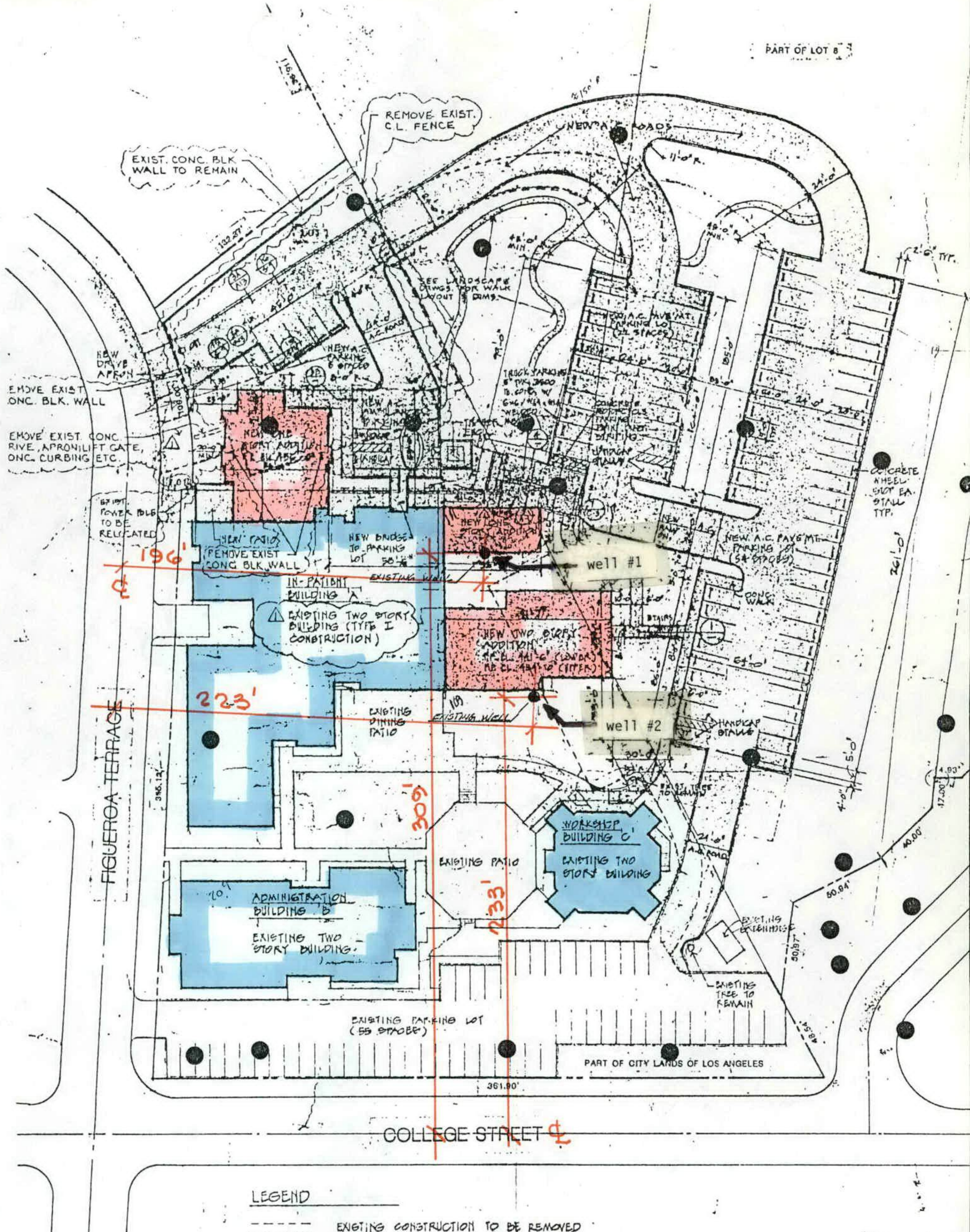

LEGEND

- - - - - EXISTING CONSTRUCTION TO BE REMOVED

EXISTING CONSTRUCTION TO REMAIN

NEW CONSTRUCTION

⊕ EXISTING WELL

REFERENCE NORTH

SITE PLAN

SCALE 1" = [illegible]

WELL # 1 = 750' ± DEEP
WELL # 2 = 400' ± DEEP

4/24/88

RECEIVED
APR 29 1 51 PM '88
DIV. OF OIL AND GAS
LONG BEACH, CA.

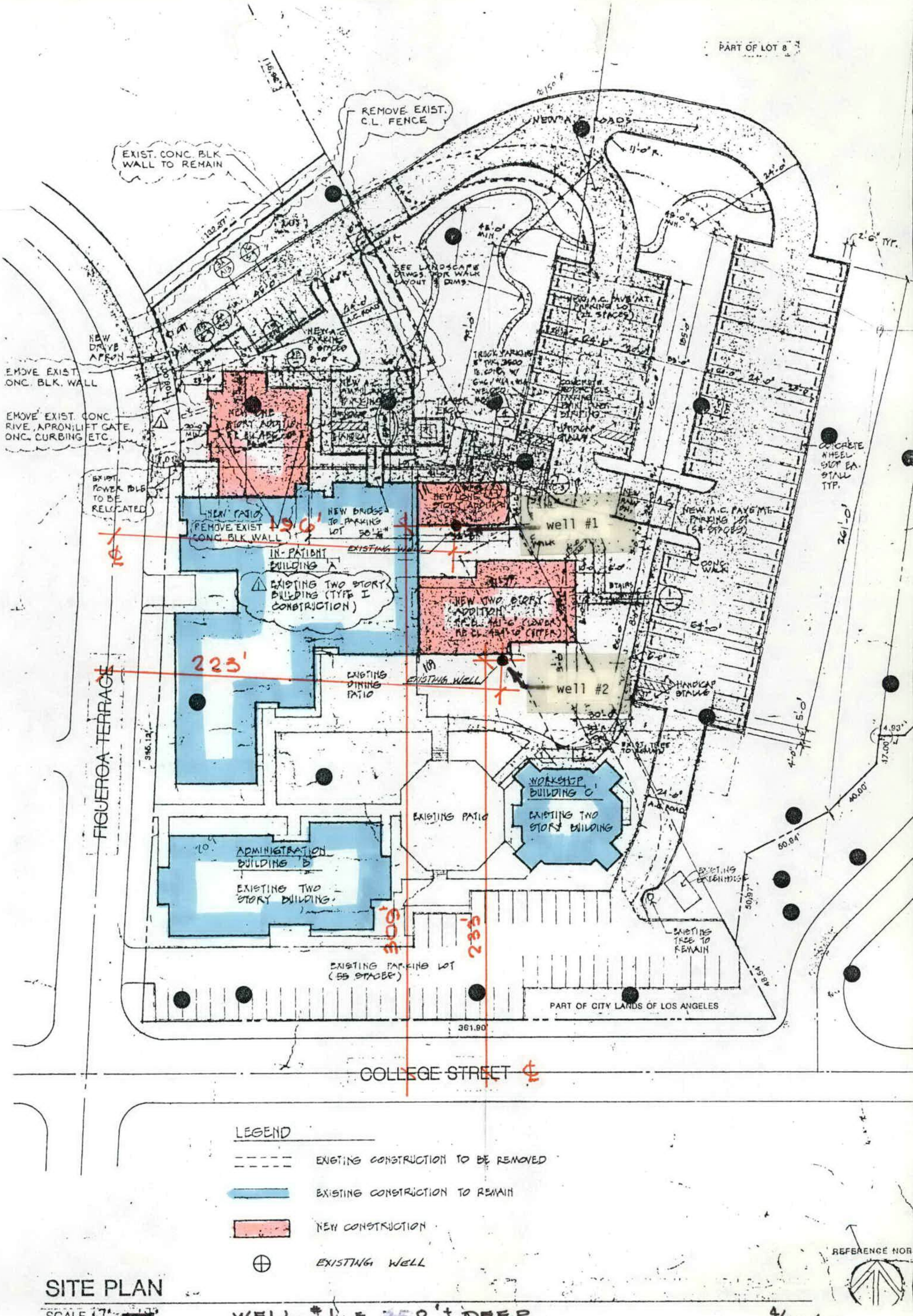

SITE PLAN

SCALE

WELL #1 = 150'± DEEP

WELL #2 = 400'± DEEP

4/26/88

RECEIVED
APR 29 1 51 PM '88
DIV. OF OIL AND GAS
LONG BEACH, CA.

EIVED
3 17 AM '88
IL AND GAS
EACH, CA.
