# Supplementary material for: Locating Undocumented Wells Using Historical Oil and Gas Exploration Maps: A Case Study in Osage County, Oklahoma
Source: Environ Sci Technol. 2026 Jul 16;60(30):20913–24. doi: 10.1021/acs.est.6c03981 (PMC13449896; doi:10.1021/acs.est.6c03981)
Supplement: Supplementary file 4 [file es6c03981_si_005.pdf]

5-425b **134423**

MISCELLANEOUS RECEIVED APR 20 1934 11451 OSAGE AGENCY

DEPARTMENT OF THE INTERIOR

UNITED STATES INDIAN SERVICE

Osage Indian Agency, Pawhuska, Okla.

PLUGGING RECORD

NOTICE—Permission must be obtained before any well is plugged.

Date of letter granting permission to plug: ✓ (Did not get letter - by phone)

LESSEE Edward C. Lawson, OSAGE NATION, LESSOR

Address Exchange Bank Bldg., Tulsa, Okla.

Well No. 1 ¼ NW Sec. 30 T. 27 R. 12 Osage County

Commenced Plugging Work April 3, 1934 Finished Work April 7, 1934

Character of Well (Whether Oil, Gas or Dry) Dry Total Depth 365 Feet

Name of producing sand Depth top 297 Ft. Bottom 365 Ft.

Show depth and thickness of all fresh water, oil and gas formations (as Big Lime, Oswego, Bartlesville, etc.)

RECORD SANDS — CASING RECORDS

Formation	From	To	Size	Put In	Pulled Out
Shale	17'	40'	8 ¼	62'	All pulled
Water	40'	50'	6¼	225'	
Shale	50'	255'			
Dry sand	255'	270'			
Shale	270'	290'			
Lime	290'	297'			
Sand	297'	365'			

Was well filled with mud fluid according to Regulations, approved March 7, 1923, governing operations of Osage leases in Oklahoma? Yes Describe the manner in which the well was plugged.

Well was filled with mud, then casing was pulled and hole filled with mud again.

Name of Inspector who supervised the plugging of this well: ✓ (Driller did not get name)

Signed: E. C. Trowbridge
Representative of Company.

REMARKS: Why plugged? Dry If abandoned oil or gas well, state amount of last production

Subscribed and sworn to before me this 18th day of April 1934

Chas Shaffer
Notary Public.

My Commission expires May 13, 1937

O. K. By O. M. Kelley Department Inspector.

Checked off on Book. W.

BIA G06 930000025646 -1
