# Supplementary material for: Locating Undocumented Wells Using Historical Oil and Gas Exploration Maps: A Case Study in Osage County, Oklahoma
Source: Environ Sci Technol. 2026 Jul 16;60(30):20913–24. doi: 10.1021/acs.est.6c03981 (PMC13449896; doi:10.1021/acs.est.6c03981)
Supplement: Supplementary file 5 [file es6c03981_si_006.pdf]

83311

5-425b

# DEPARTMENT OF THE INTERIOR

UNITED STATES INDIAN SERVICE

Osage Indian Agency, Pawhuska, Okla.

## PLUGGING RECORD

MISCELLANEOUS RECEIVED FEB 7 1931 No. 5342 OSAGE AGENCY

NOTICE—Permission must be obtained before any well is plugged.

Date of letter granting permission to plug: January 20, 1931

LESSEE INDIAN TERRITORY ILLUMINATING OIL COMPANY, OSAGE NATION, LESSOR

Address DRAWER L BARTLESVILLE, OKLAHOMA

Well No. 157 ¼ SW Sec. 34 T. 29 R. 10 Osage County

Commenced Plugging Work January 16, 1931 Finished Work January 30, 1931

Character of Well (Whether Oil, Gas or Dry) GAS Total Depth 1726

Name of producing sand Mississippi Lime Depth top 1651 Bottom 1726

Show depth and thickness of all fresh water, oil and gas formations (as Big Lime, Oswego, Bartlesville, etc.)

RECORD SANDS | CASING RECORDS

| Formation | From | To | Size | Put In | Pulled Out |
|---|---|---|---|---|---|
| Oswego Lime | 1248 | 1315 | 12½ | 29' | None |
| Mississippi Lime | 1651 | 1726 | 8¼ | 738'7" | 738'7" |
| | | | 6-5/8 | 1659'10" | 1659'10" |
| | | | 3" | 64'9" | 64'9" |
| | | | 2" | 1664'4" | 1664'4" |
| | | | 3/4" | 1706'7" | 1706'7" |

Was well filled with mud fluid according to Regulations, approved March 7, 1923, governing operations of Osage leases in Oklahoma? Yes Describe the manner in which the well was plugged.

Well was filled with heavy mud from bottom to top as casing was pulled.

Name of Inspector who supervised the plugging of this well: O. M. Kelly

Signed: H B Peterson
Representative of Company.

REMARKS: Why plugged? Exhausted If abandoned oil or gas well, state amount of last production 71,000

Subscribed and sworn to before me this 6 day of February 1931

J. C. Williamson
Notary Public.

My Commission expires January 20, 1934.

O. K. By O. M. Kelly Department Inspector.

BIA G06 930000053451 -1
