# Supplementary material for: Locating Undocumented Wells Using Historical Oil and Gas Exploration Maps: A Case Study in Osage County, Oklahoma
Source: Environ Sci Technol. 2026 Jul 16;60(30):20913–24. doi: 10.1021/acs.est.6c03981 (PMC13449896; doi:10.1021/acs.est.6c03981)
Supplement: Supplementary file 6 [file es6c03981_si_007.pdf]

12340 6

132795

DEPARTMENT OF THE INTERIOR

UNITED STATES INDIAN SERVICE
Osage Indian Agency, Pawhuska, Okla.

MISCELLANEOUS RECEIVED
JUN 23 1924
NO. 15443
OSAGE AGENCY

PLUGGING RECORD

NOTICE—Permission must be obtained before any well is plugged or abandoned.

LESSEE W. H. Foster, OSAGE NATION, LESSOR.

Well No. 3. SE ¼ SE Sec. 33 T. 25 R. 12. E. Osage County

Commenced Plugging Work June 18 th Finished Work June 20 th 1924

Character of Well (Whether Oil, Gas or Dry) Dry hole Total depth 1826

Name of producing sand .... Depth top .... Bottom ....

Show depth and thickness of all fresh water, oil and gas formations (as Big Lime, Oswego, Bartlesville, etc.

RECORD SANDS / CASING RECORDS.

Formation	From	To	Size	Put In	Pulled Out
Sand	500	545	8-1/4	551	551
Big lime	974	1060			
Perue sand	1084	1153			
Oswego lime	1230	1304			
Bartlesville Sand	1680	1757			
			5-3/16	1760	1760
burgess sand	1813	1816			
Top Missippi lime	1816	1826			

Was well filled with mud fluid according to Regulations, approved October 20, 1915, governing operations on Indian Lands in Oklahoma? Yes Describe the manner in which the well was plugged.

Seven hundred feet mud laden fluid put inside 5-3/16 casing and then casing pulled hole filled within ninty feet of top with mud fluid, and 8-1/4 pulled and hole filled to top with mud and capped according to regulationsof the Department Interior.

Signed: W H Foster
Representative of Company.

REMARKS: Why plugged? Dry hole If abandoned oil or gas well, state amount of last production

Subscribed and sworn to before me this 21 day of June 1924

My Commission expires My Commission Expires Apr. 23, 1927 W. W. Turner
Notary Public.

O. K. By W B Gaither Department Inspector.

BIA G06 930000070010 -1
